# Supplementary material for: Body size-dependent energy storage causes Kleiber’s law scaling of the metabolic rate in planarians
Source: eLife. 2019 Jan 4;8:e38187. doi: 10.7554/eLife.38187 (PMC6320072; doi:10.7554/eLife.38187)
Supplement: Figure 2—source data 3. [file elife-38187-fig2-data3.zip › Figure 2 - source data 3/Experiment 4/Info Experiment 4.rtf]

12 small animals in totalPlate A: animals 1 - 6Plate B: animals 7 - 12One animal per row (10 wells/animal)See Figure 2 - figure suppl 1 - source data 2 for CellProfiler pipeline.Quality control columns mark images with no cells or beads (FlagImage module).
