# Supplementary material for: Body size-dependent energy storage causes Kleiber’s law scaling of the metabolic rate in planarians
Source: eLife. 2019 Jan 4;8:e38187. doi: 10.7554/eLife.38187 (PMC6320072; doi:10.7554/eLife.38187)
Supplement: Figure 2—figure supplement 2—source data 1. [file elife-38187-fig2-figsupp2-data1.zip › Figure 2 - figure suppl 2 - source data/Comment CellProfiler pipeline.rtf]

Works with CellProfiler version 2, some modules may not work in version 3.Quality control columns mark images with no cells or beads (FlagImage module).
