# Supplementary figures and images for: Body size-dependent energy storage causes Kleiber’s law scaling of the metabolic rate in planarians (part 2 of 3)
Source: eLife. 2019 Jan 4;8:e38187. doi: 10.7554/eLife.38187 (PMC6320072; doi:10.7554/eLife.38187)

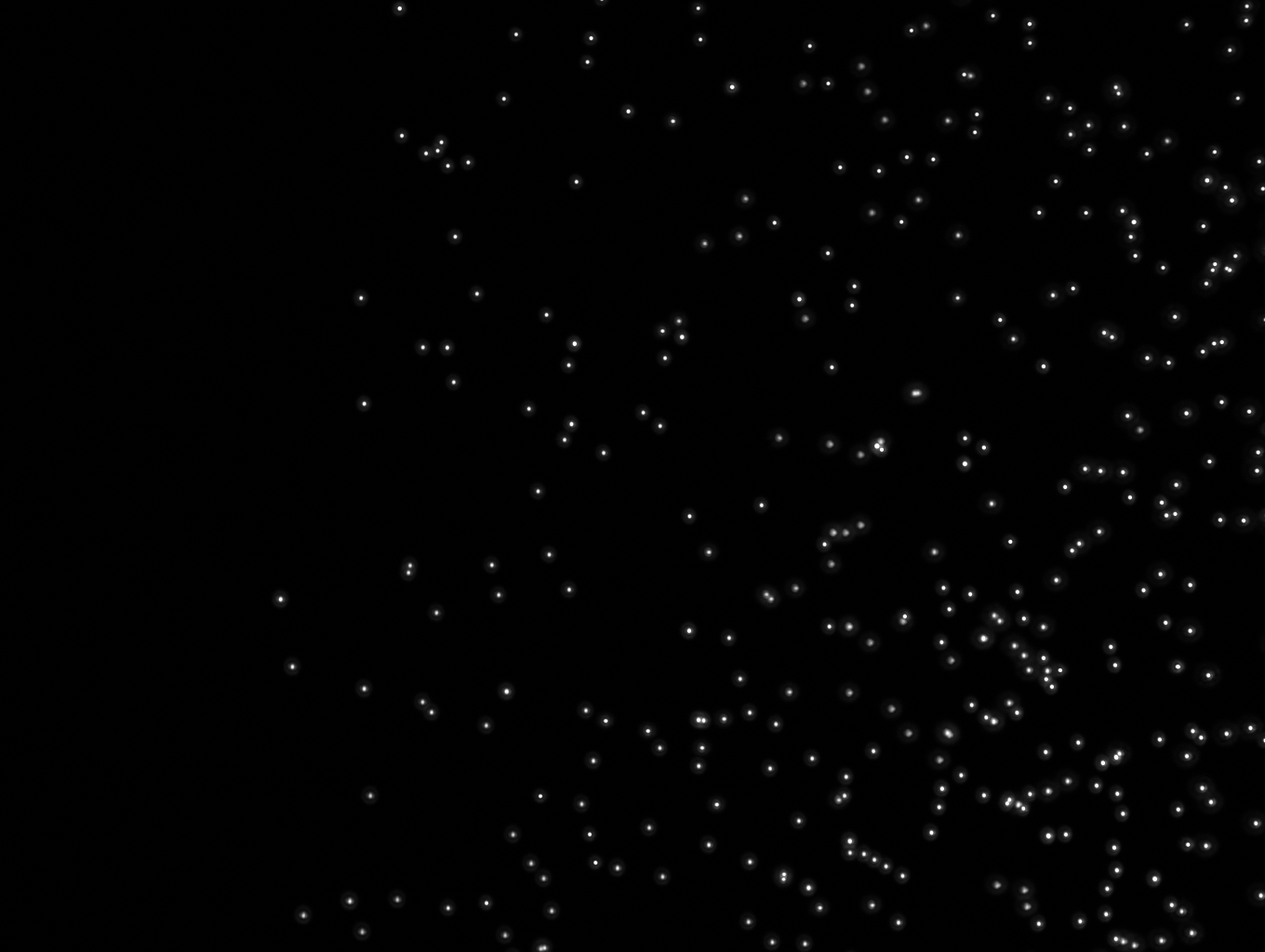

Supplement: Figure 2—figure supplement 2—source data 1. [file elife-38187-fig2-figsupp2-data1.zip › Figure 2 - figure suppl 2 - source data/Exp2/Plate B/r03c10f01p03-ch2sk1fk1fl1.tif]

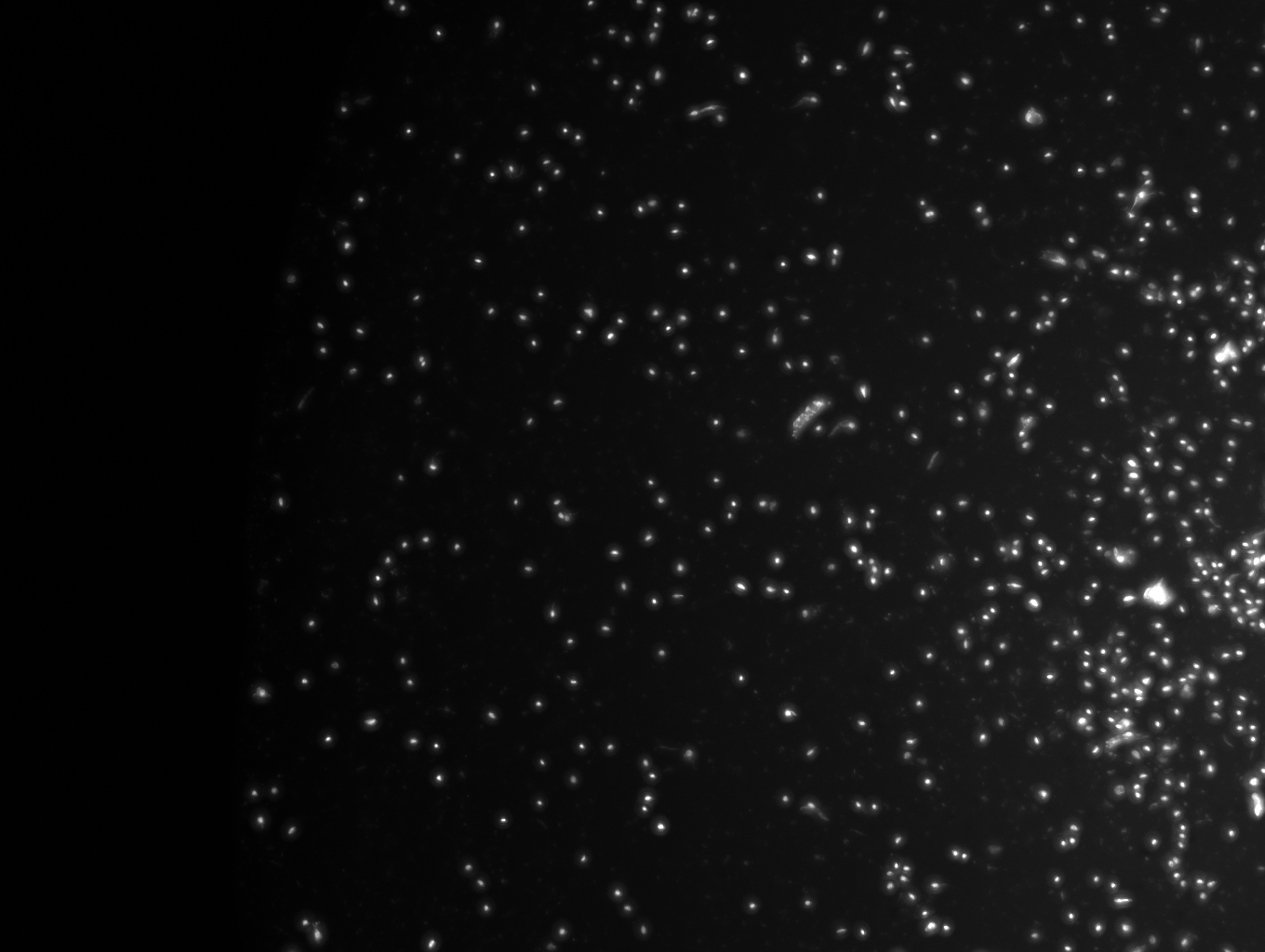

Supplement: Figure 2—figure supplement 2—source data 1. [file elife-38187-fig2-figsupp2-data1.zip › Figure 2 - figure suppl 2 - source data/Exp2/Plate B/r03c10f01p05-ch1sk1fk1fl1.tif]

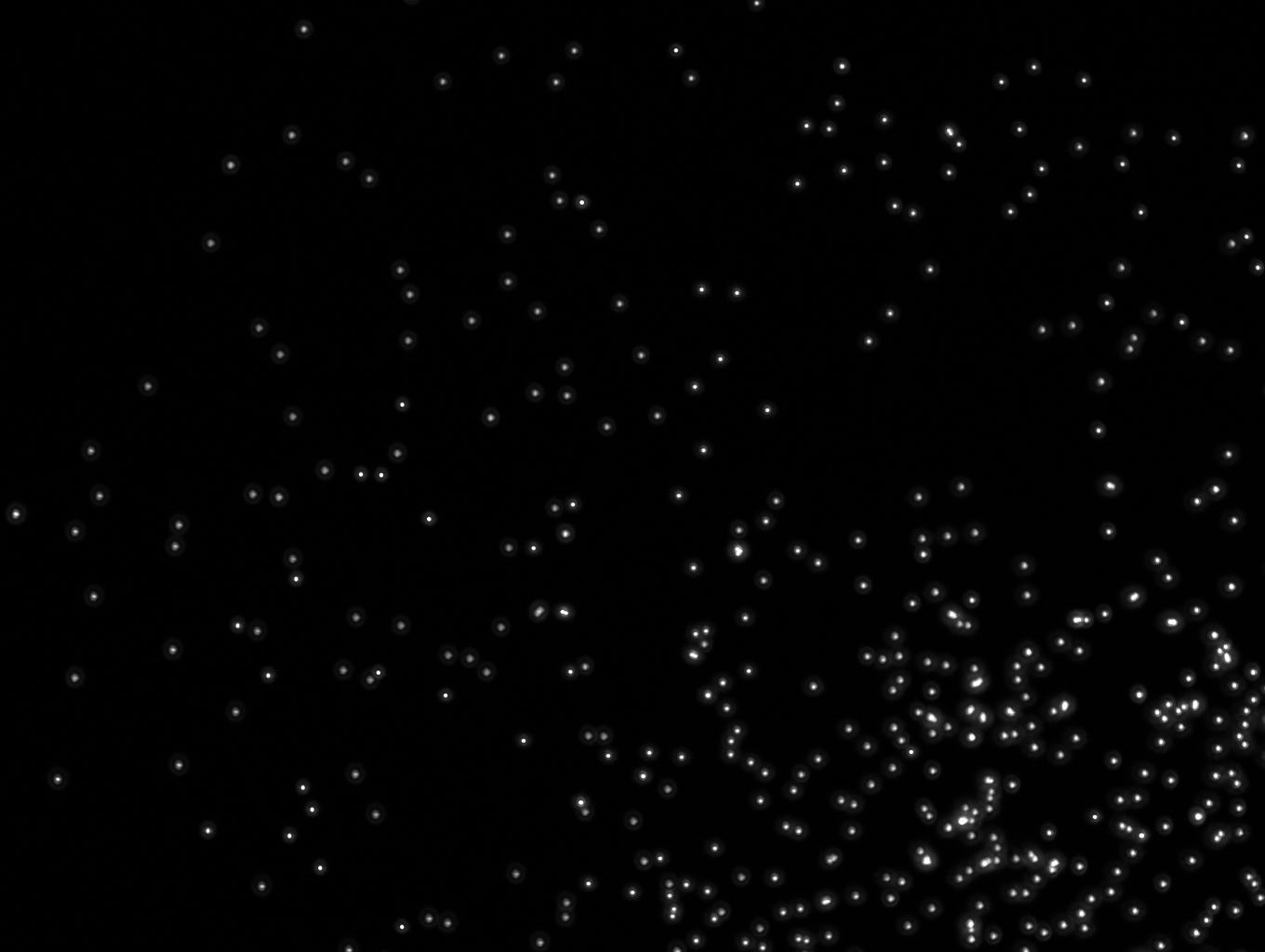

Supplement: Figure 2—figure supplement 2—source data 1. [file elife-38187-fig2-figsupp2-data1.zip › Figure 2 - figure suppl 2 - source data/Exp2/Plate B/r04c05f01p03-ch2sk1fk1fl1.tif]

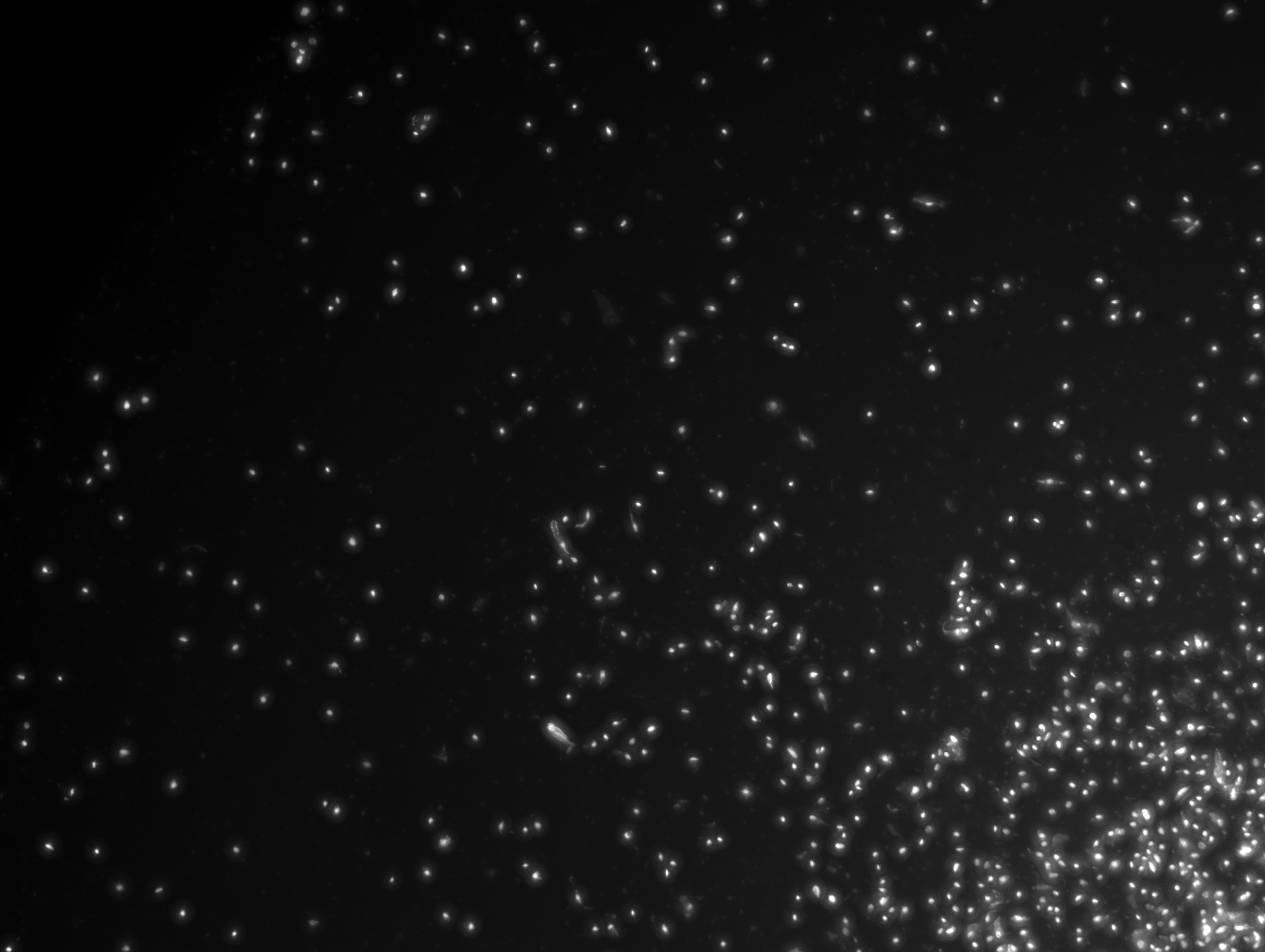

Supplement: Figure 2—figure supplement 2—source data 1. [file elife-38187-fig2-figsupp2-data1.zip › Figure 2 - figure suppl 2 - source data/Exp2/Plate B/r04c05f01p05-ch1sk1fk1fl1.tif]

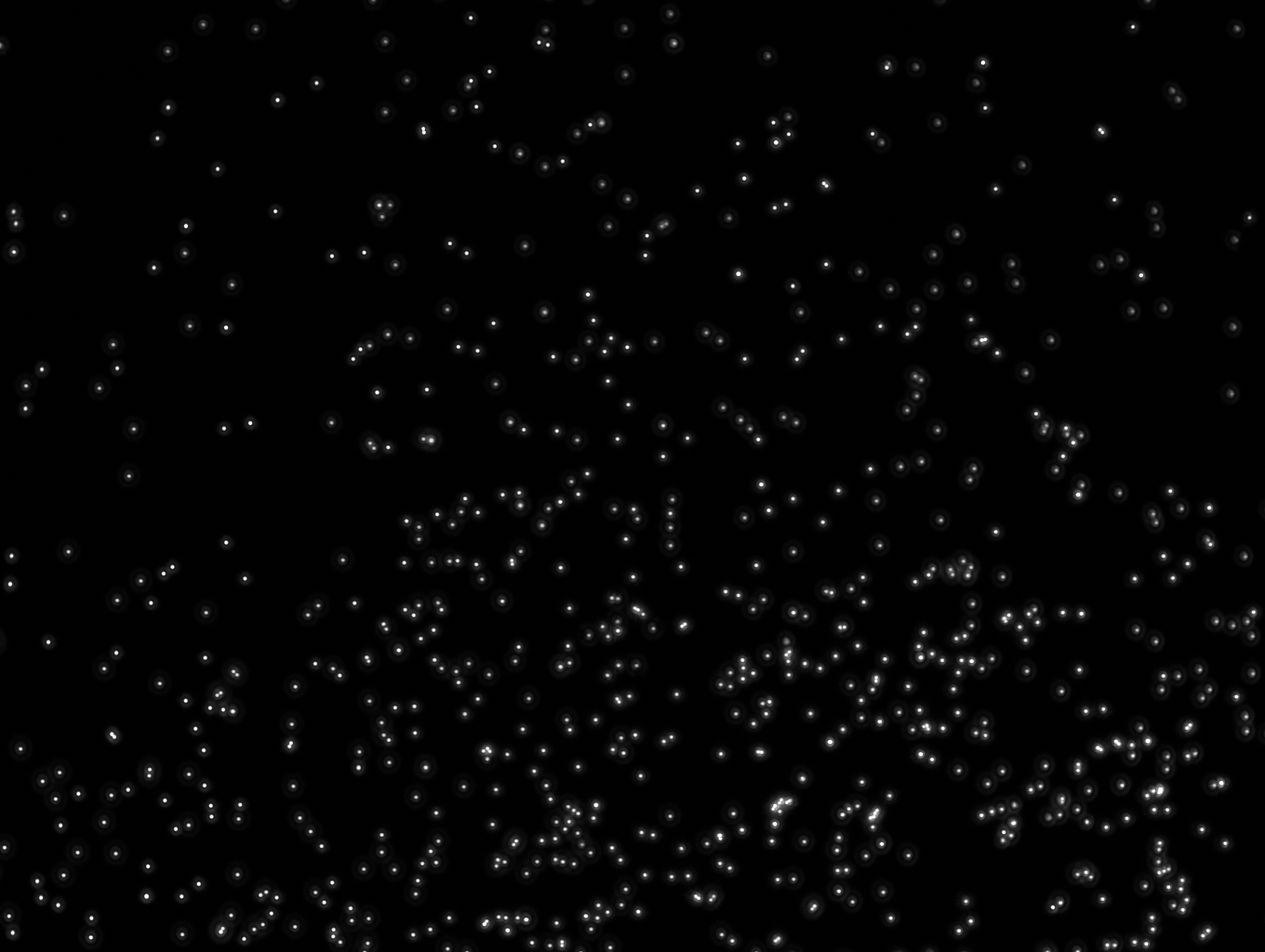

Supplement: Figure 2—figure supplement 2—source data 1. [file elife-38187-fig2-figsupp2-data1.zip › Figure 2 - figure suppl 2 - source data/Exp2/Plate B/r04c06f01p03-ch2sk1fk1fl1.tif]

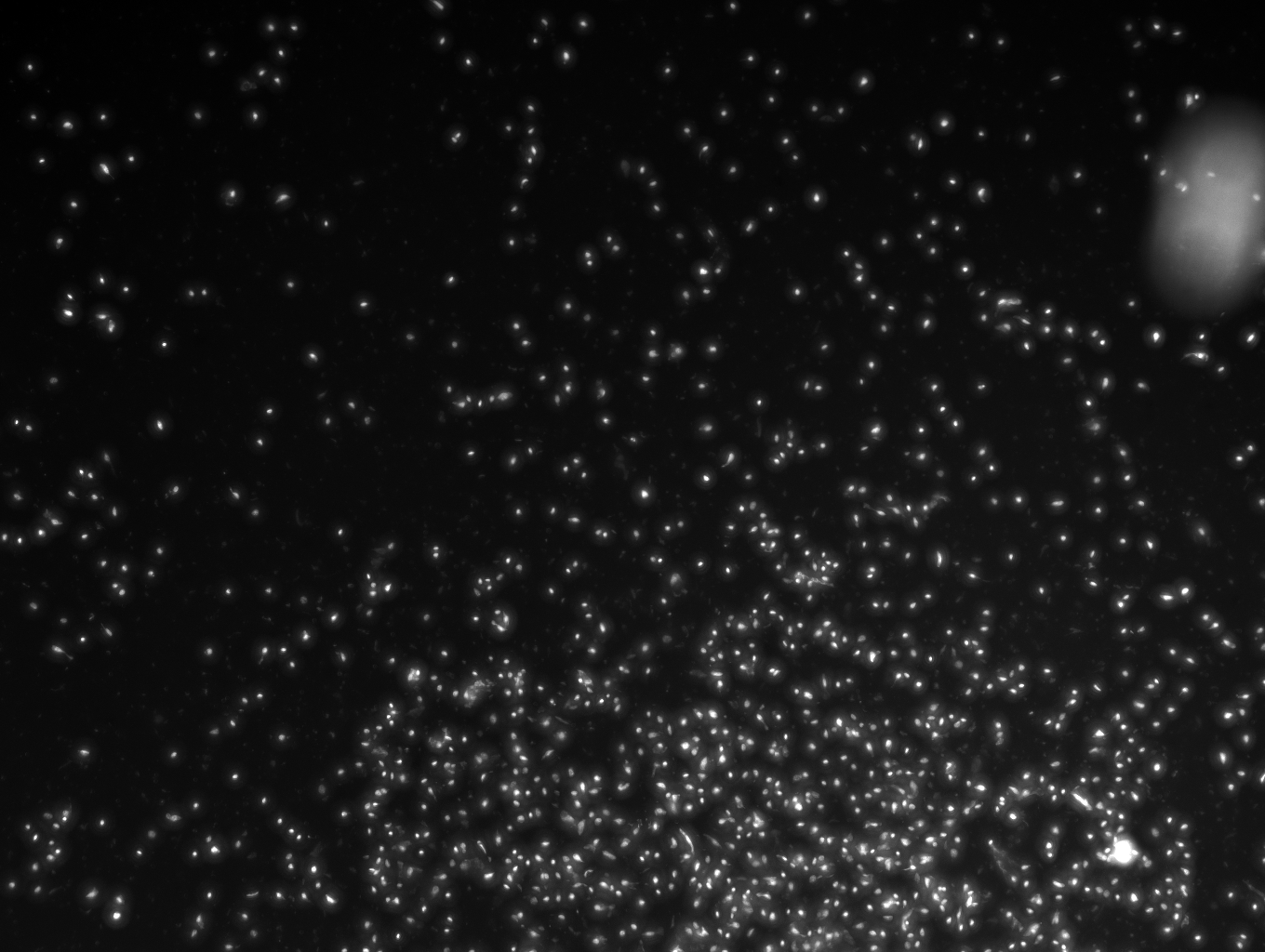

Supplement: Figure 2—figure supplement 2—source data 1. [file elife-38187-fig2-figsupp2-data1.zip › Figure 2 - figure suppl 2 - source data/Exp2/Plate B/r04c06f01p05-ch1sk1fk1fl1.tif]

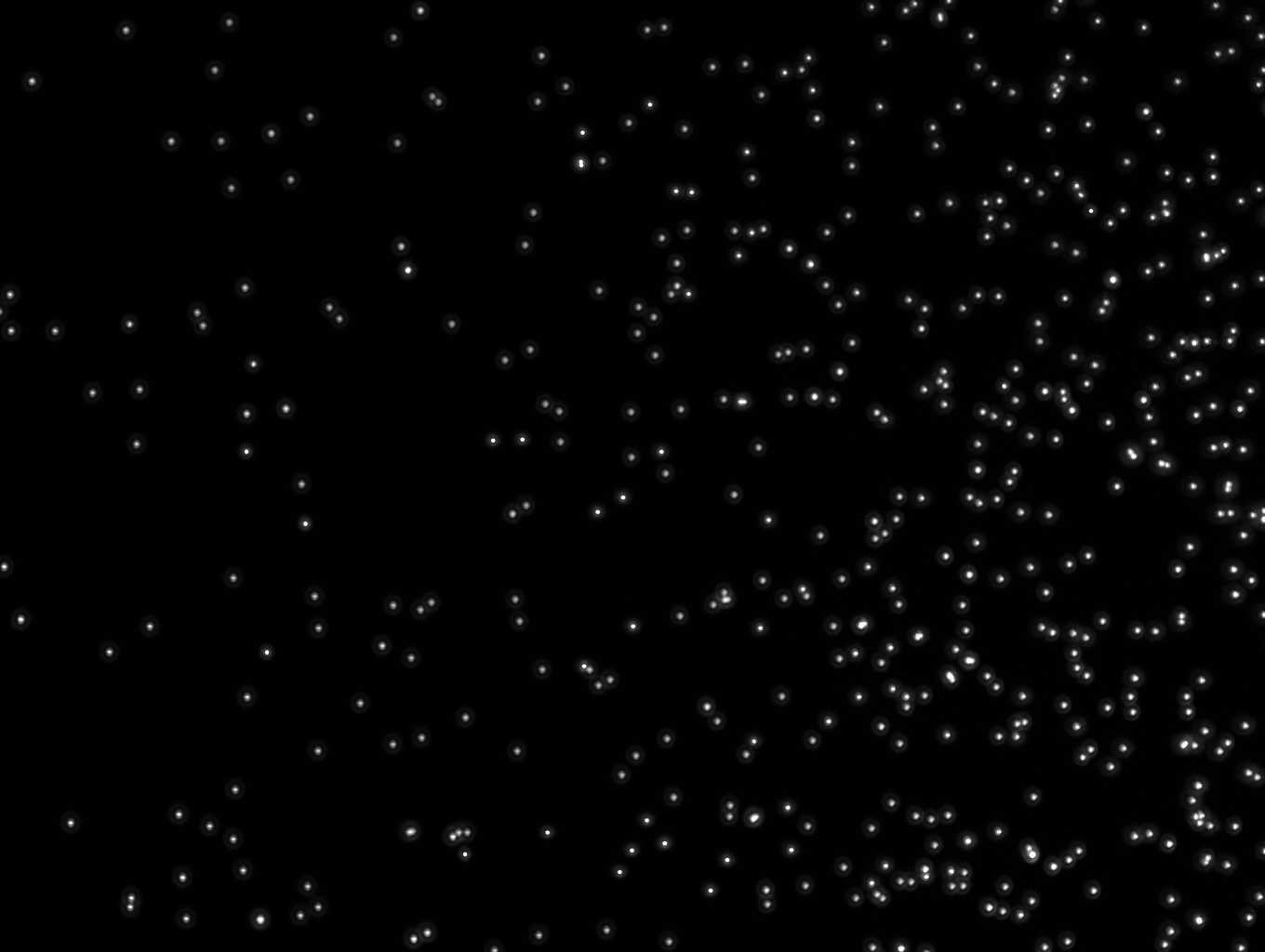

Supplement: Figure 2—figure supplement 2—source data 1. [file elife-38187-fig2-figsupp2-data1.zip › Figure 2 - figure suppl 2 - source data/Exp2/Plate B/r05c06f01p03-ch2sk1fk1fl1.tif]

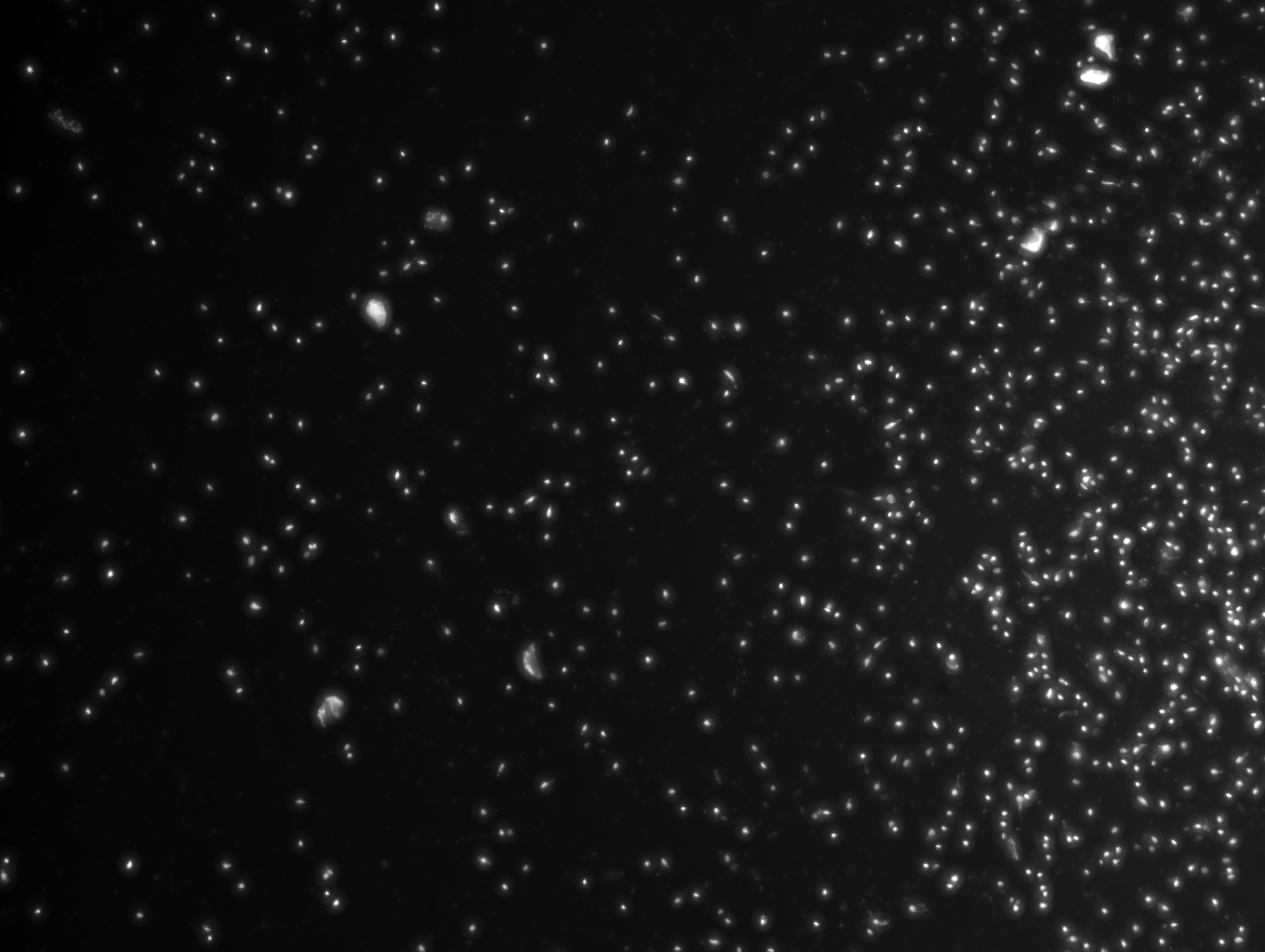

Supplement: Figure 2—figure supplement 2—source data 1. [file elife-38187-fig2-figsupp2-data1.zip › Figure 2 - figure suppl 2 - source data/Exp2/Plate B/r05c06f01p05-ch1sk1fk1fl1.tif]

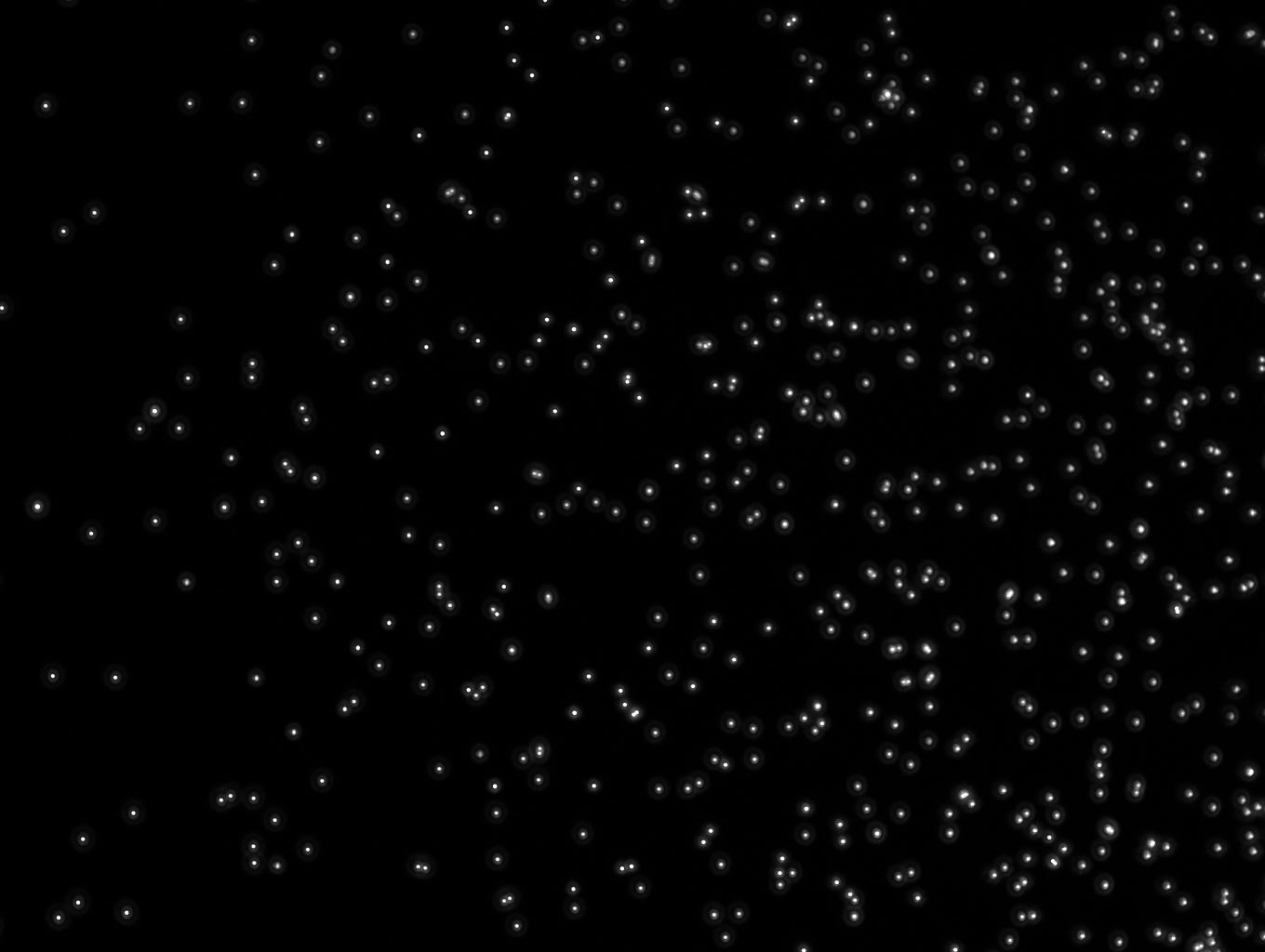

Supplement: Figure 2—figure supplement 2—source data 1. [file elife-38187-fig2-figsupp2-data1.zip › Figure 2 - figure suppl 2 - source data/Exp2/Plate B/r05c09f01p03-ch2sk1fk1fl1.tif]

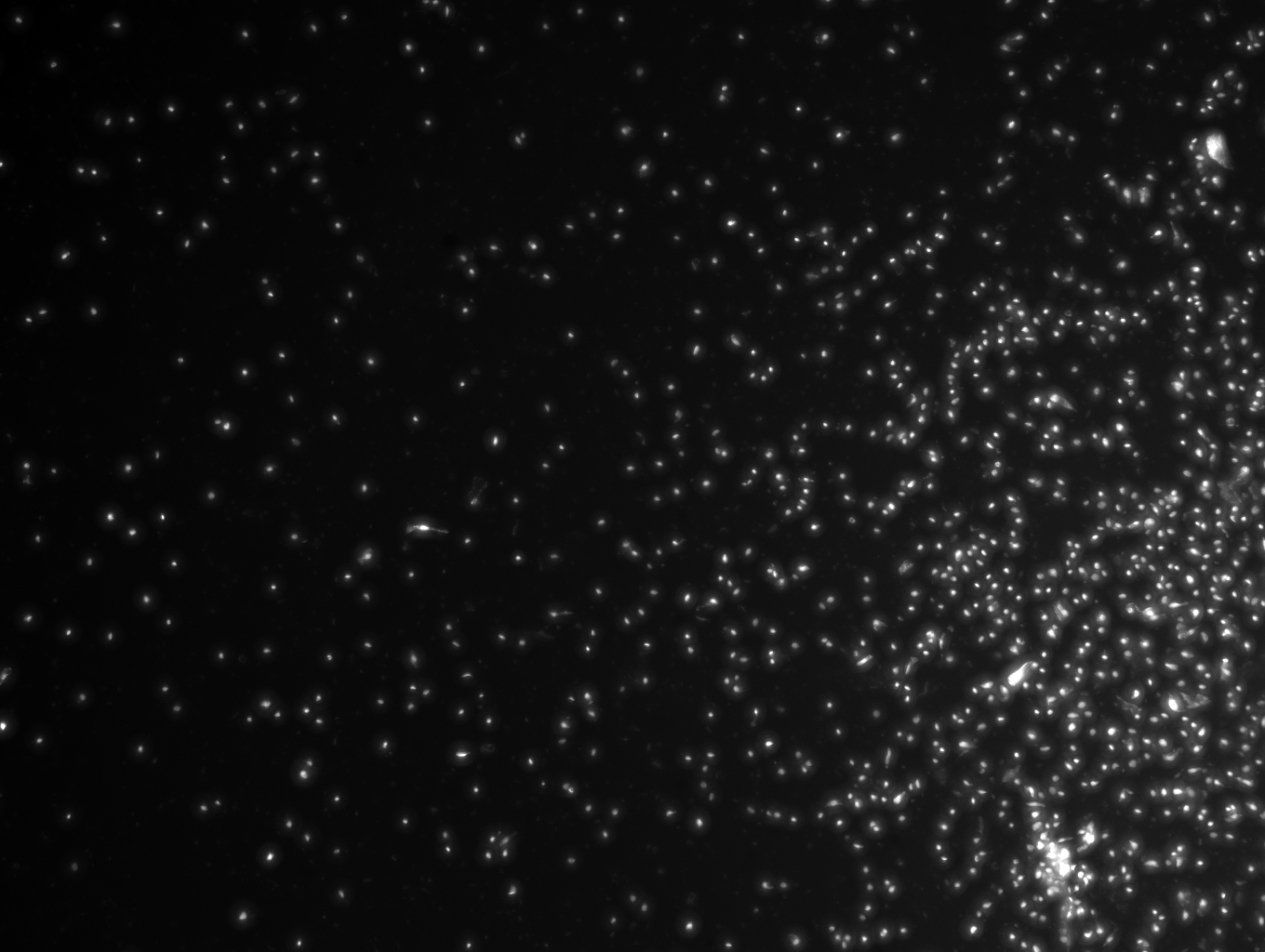

Supplement: Figure 2—figure supplement 2—source data 1. [file elife-38187-fig2-figsupp2-data1.zip › Figure 2 - figure suppl 2 - source data/Exp2/Plate B/r05c09f01p05-ch1sk1fk1fl1.tif]

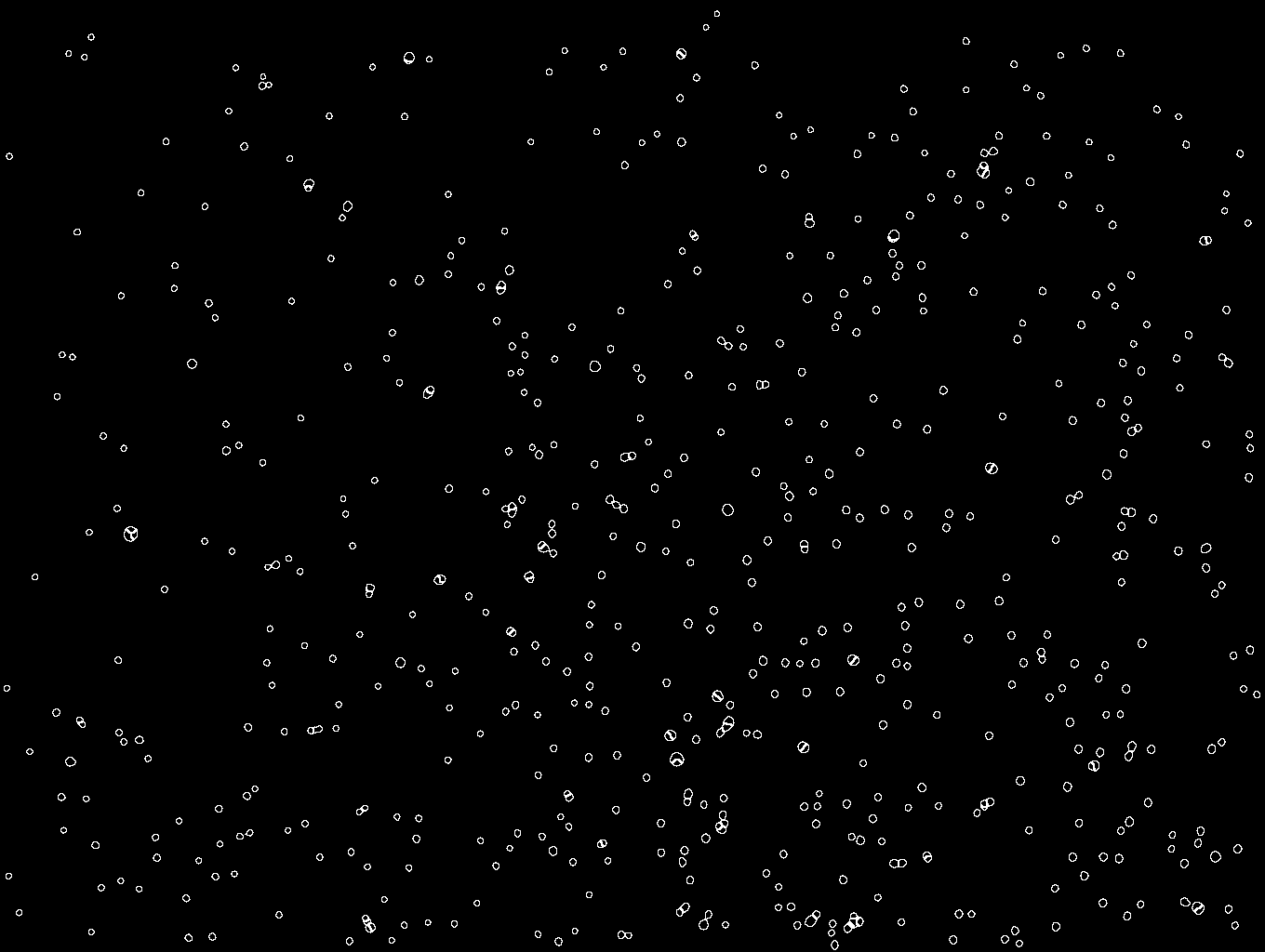

Supplement: Figure 2—figure supplement 2—source data 1. [file elife-38187-fig2-figsupp2-data1.zip › Figure 2 - figure suppl 2 - source data/Exp2/Plate B/Segmentation/r02c03f01p03-beads_outline.tif.tif]

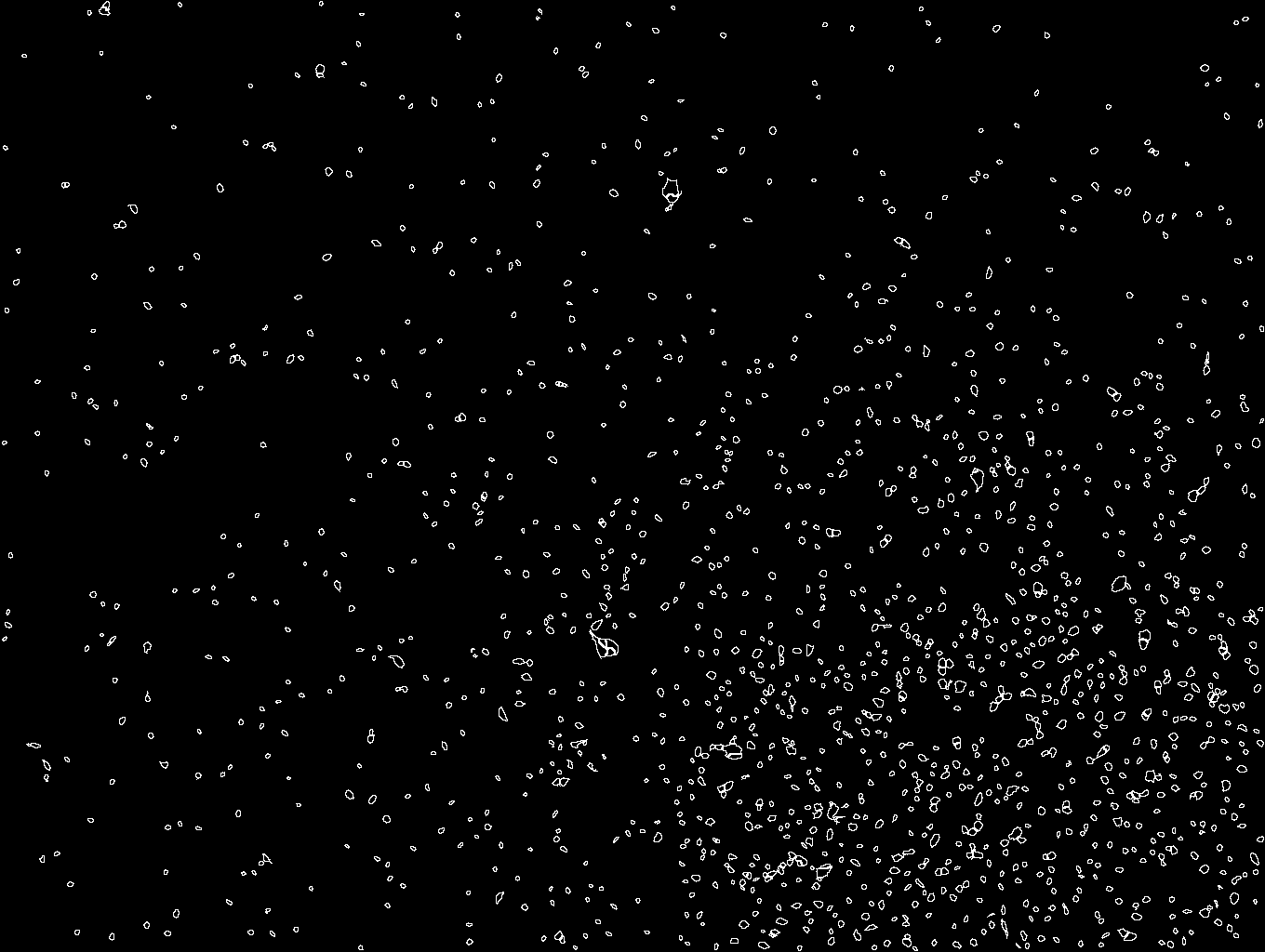

Supplement: Figure 2—figure supplement 2—source data 1. [file elife-38187-fig2-figsupp2-data1.zip › Figure 2 - figure suppl 2 - source data/Exp2/Plate B/Segmentation/r02c03f01p05-cell_outline.tif.tif]

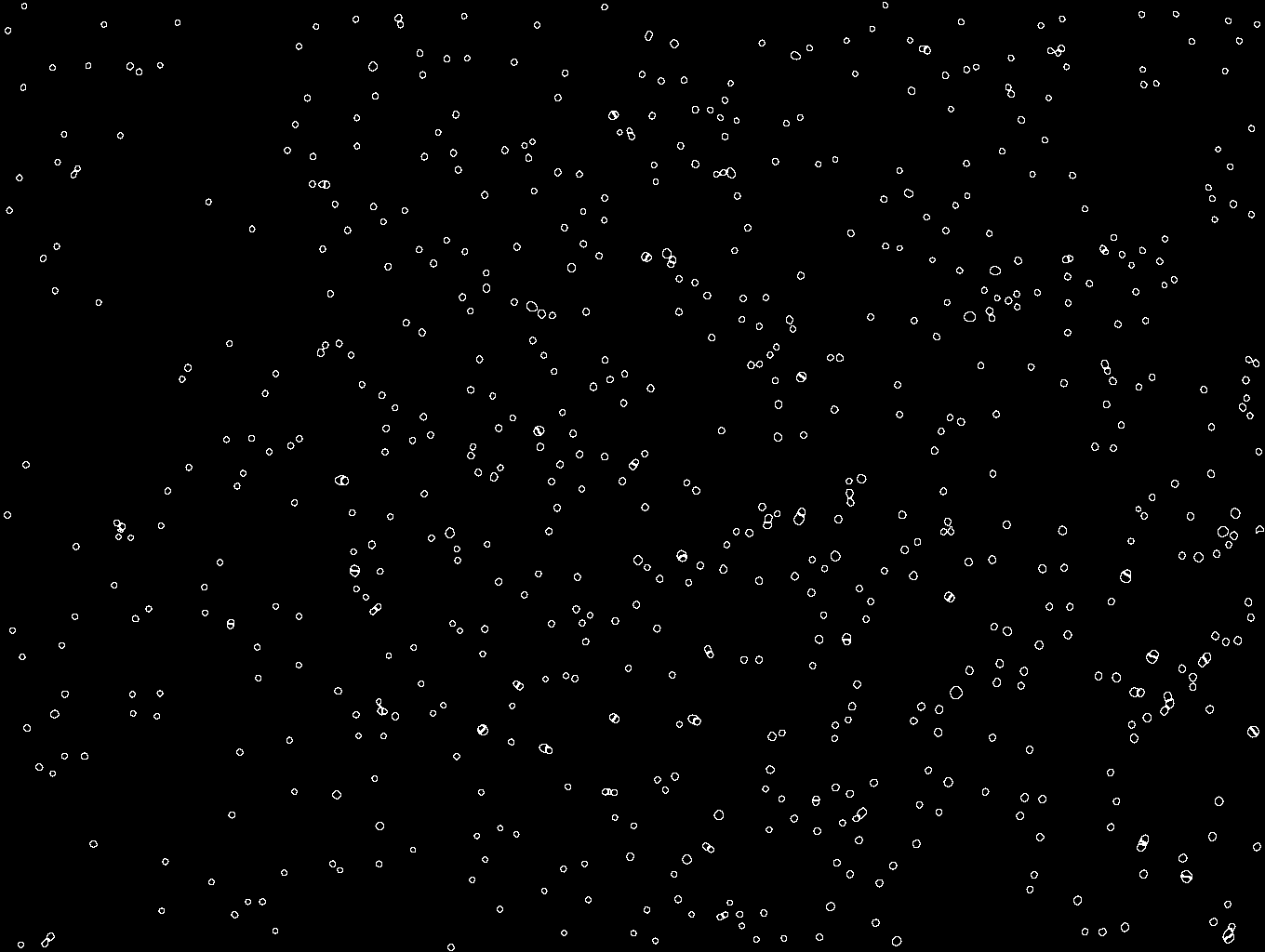

Supplement: Figure 2—figure supplement 2—source data 1. [file elife-38187-fig2-figsupp2-data1.zip › Figure 2 - figure suppl 2 - source data/Exp2/Plate B/Segmentation/r02c04f01p03-beads_outline.tif.tif]

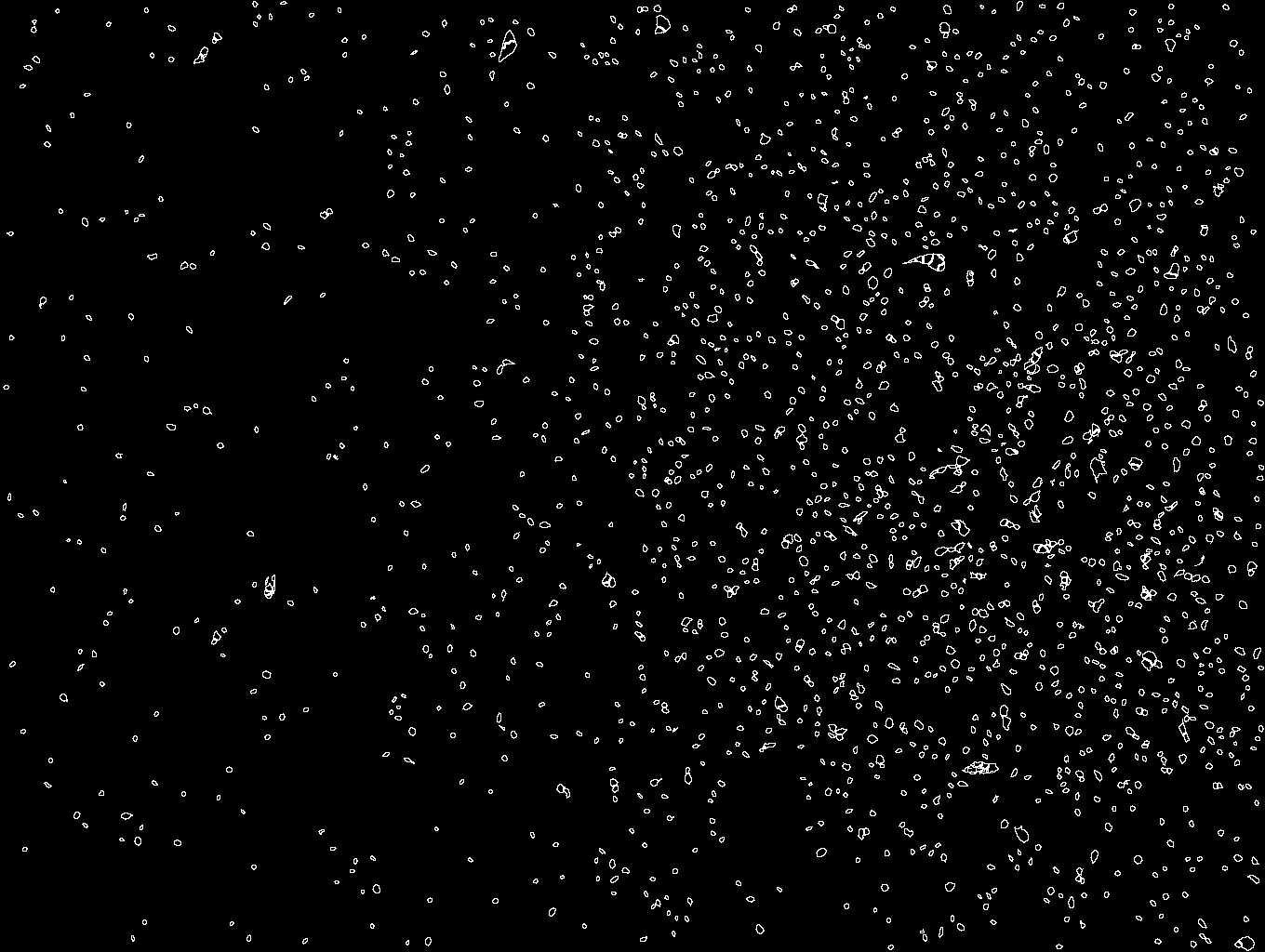

Supplement: Figure 2—figure supplement 2—source data 1. [file elife-38187-fig2-figsupp2-data1.zip › Figure 2 - figure suppl 2 - source data/Exp2/Plate B/Segmentation/r02c04f01p05-cell_outline.tif.tif]

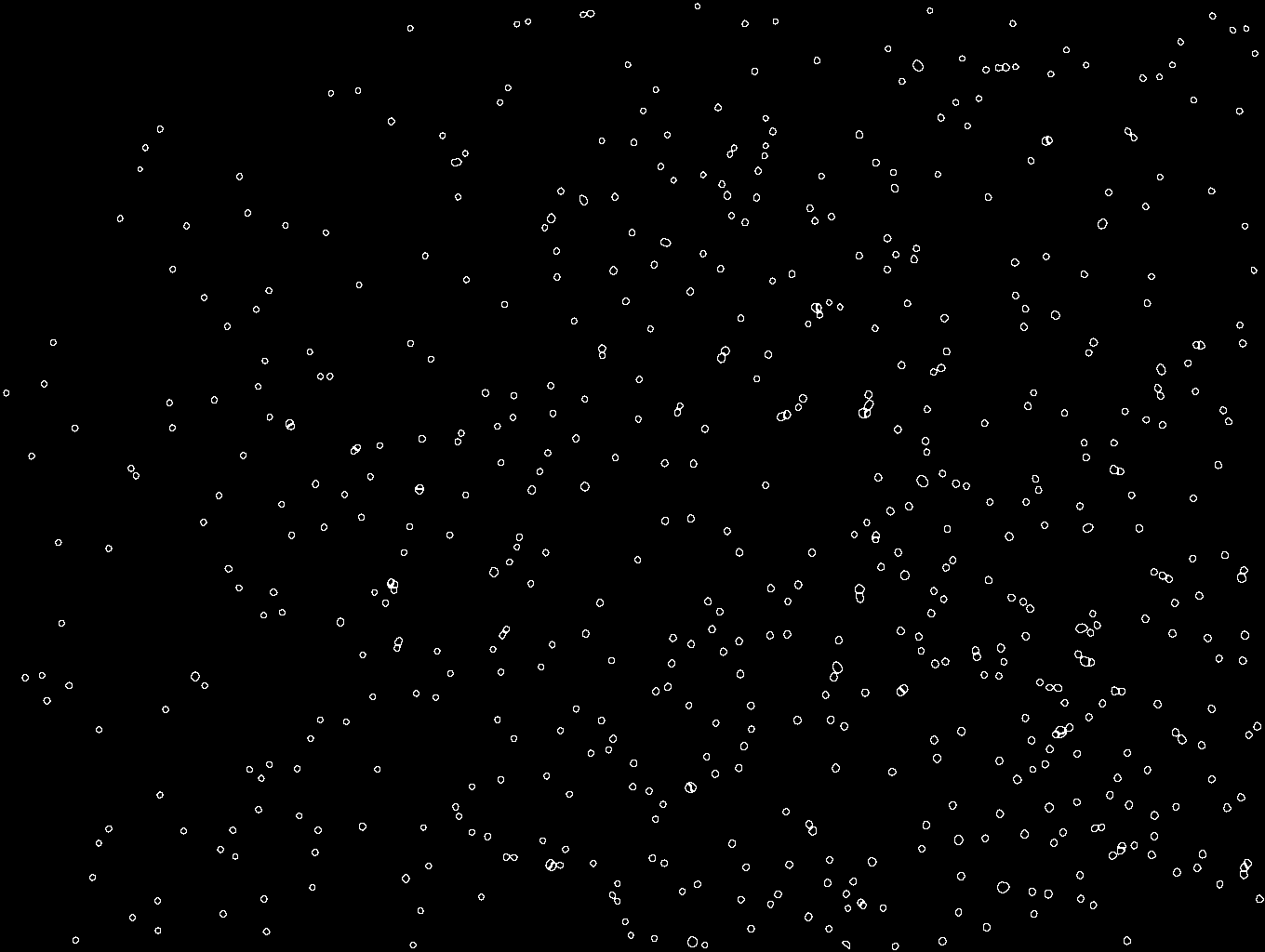

Supplement: Figure 2—figure supplement 2—source data 1. [file elife-38187-fig2-figsupp2-data1.zip › Figure 2 - figure suppl 2 - source data/Exp2/Plate B/Segmentation/r02c05f01p03-beads_outline.tif.tif]

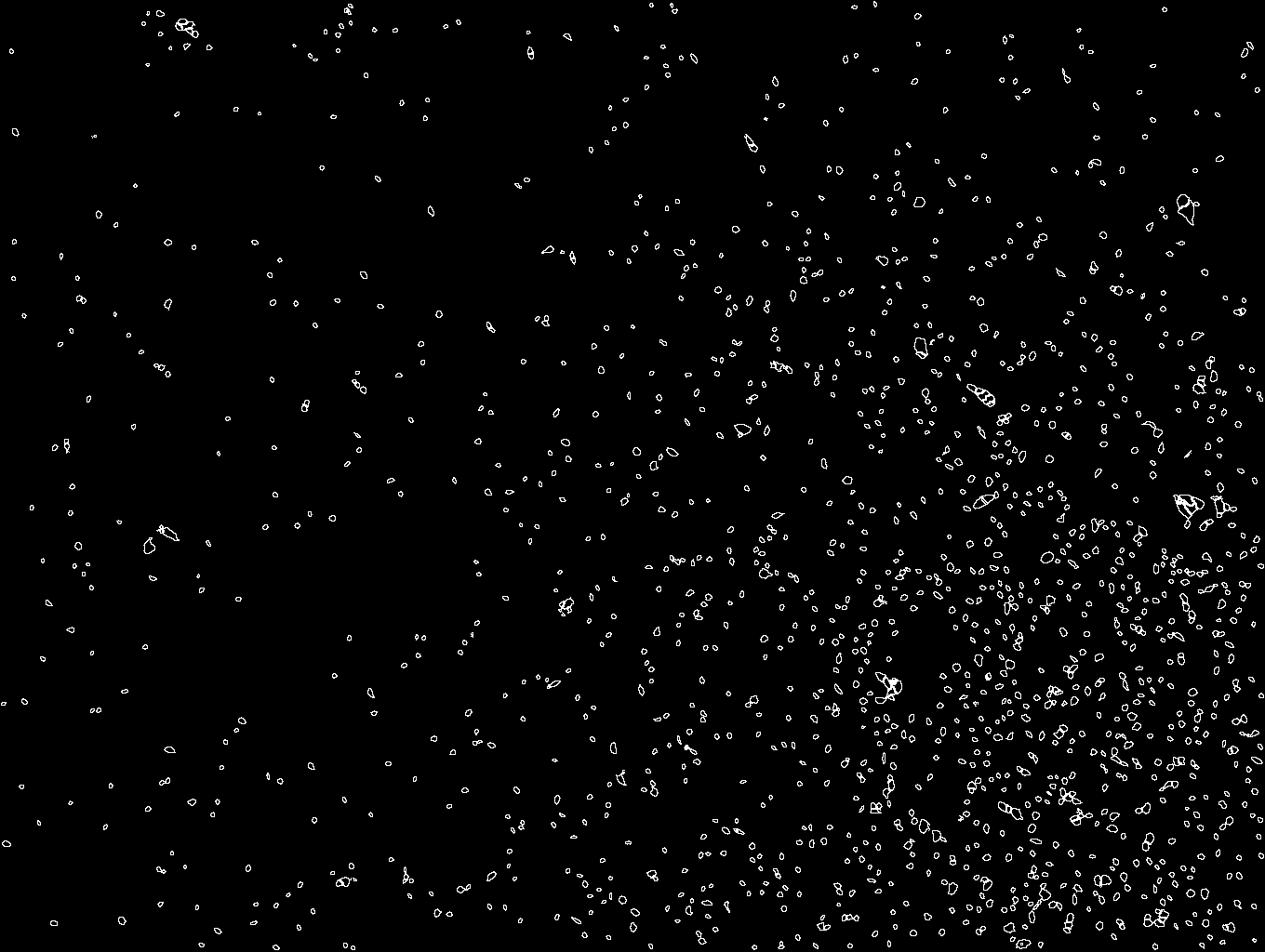

Supplement: Figure 2—figure supplement 2—source data 1. [file elife-38187-fig2-figsupp2-data1.zip › Figure 2 - figure suppl 2 - source data/Exp2/Plate B/Segmentation/r02c05f01p05-cell_outline.tif.tif]

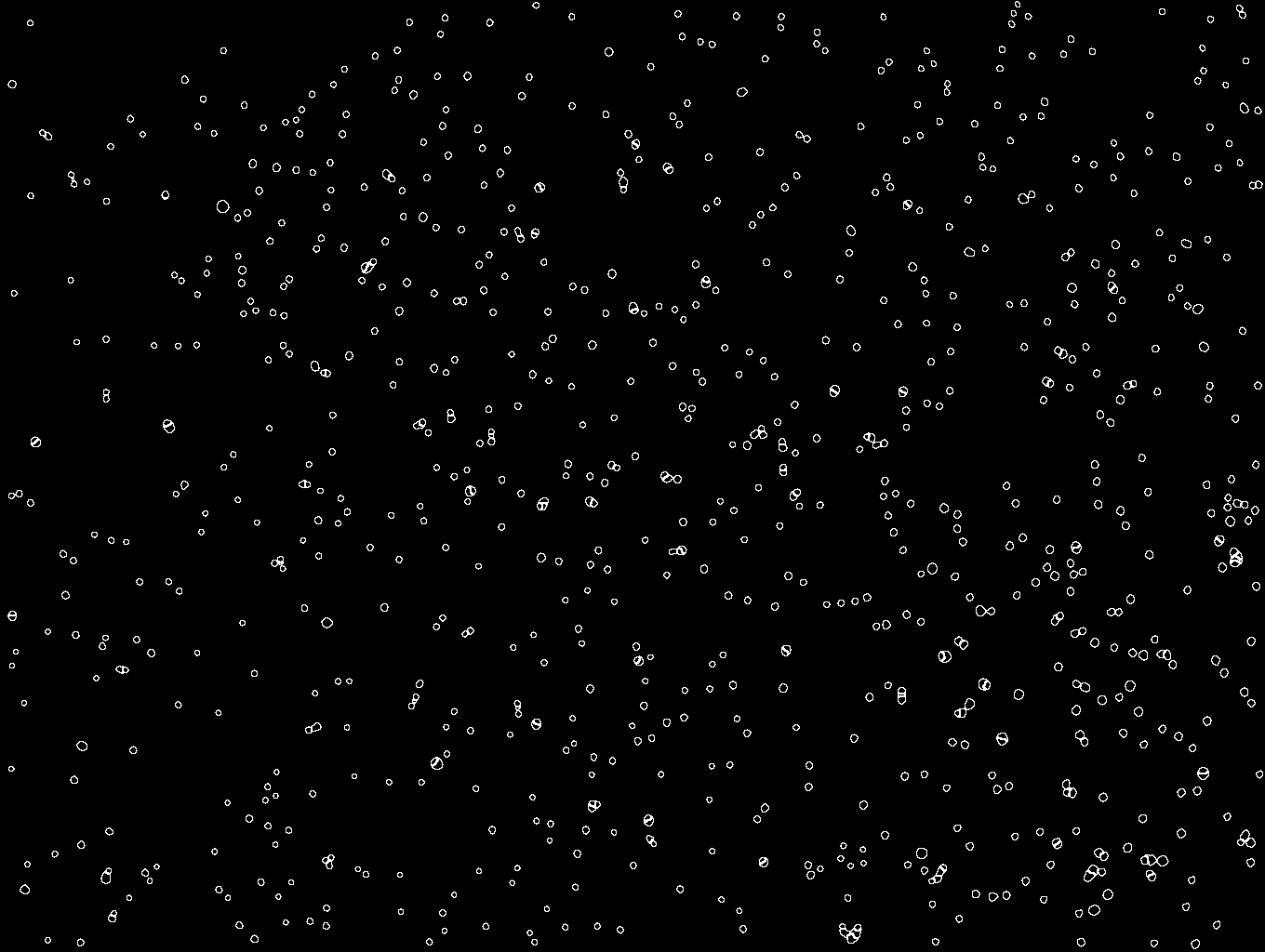

Supplement: Figure 2—figure supplement 2—source data 1. [file elife-38187-fig2-figsupp2-data1.zip › Figure 2 - figure suppl 2 - source data/Exp2/Plate B/Segmentation/r02c06f01p03-beads_outline.tif.tif]

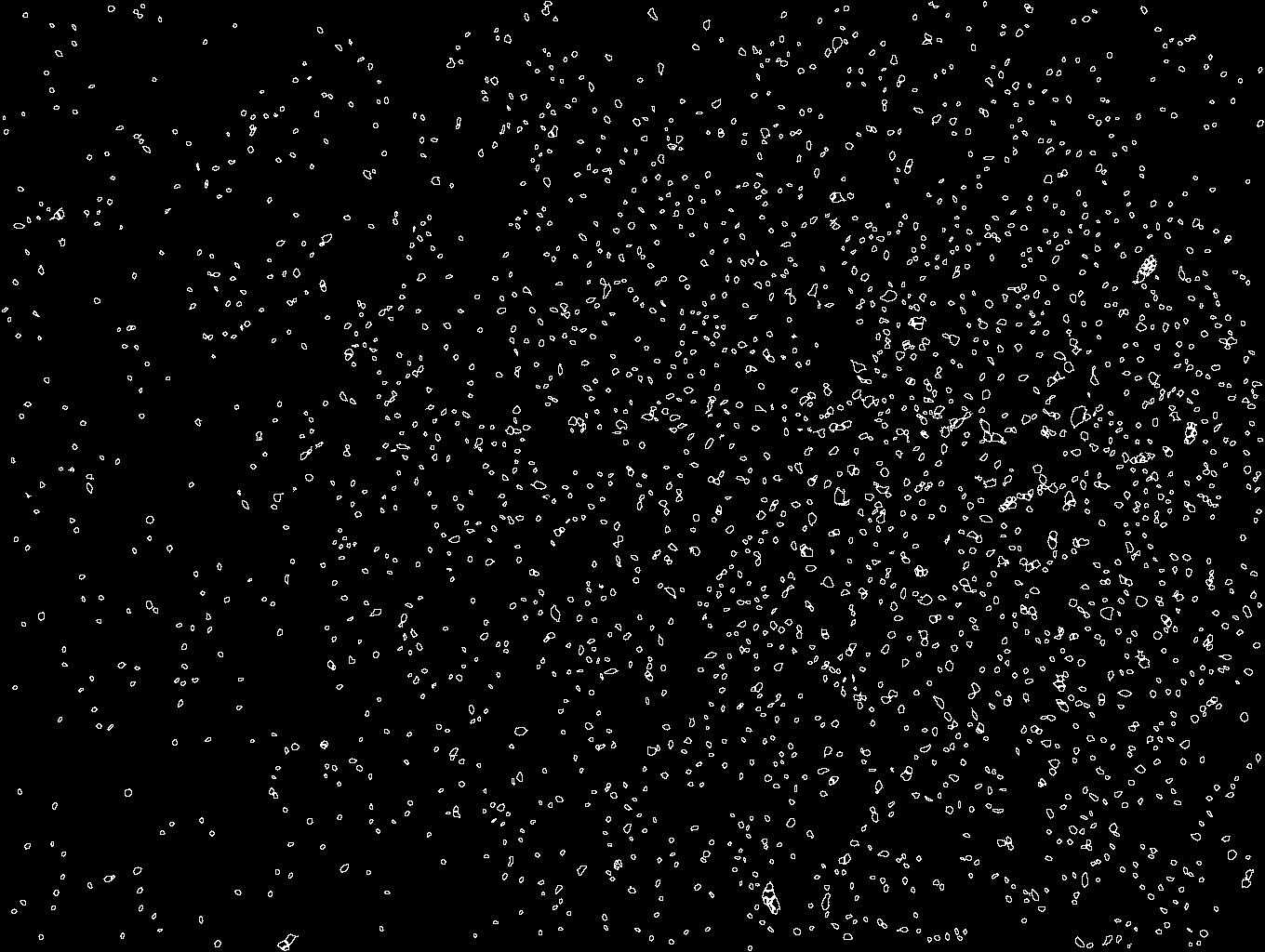

Supplement: Figure 2—figure supplement 2—source data 1. [file elife-38187-fig2-figsupp2-data1.zip › Figure 2 - figure suppl 2 - source data/Exp2/Plate B/Segmentation/r02c06f01p05-cell_outline.tif.tif]

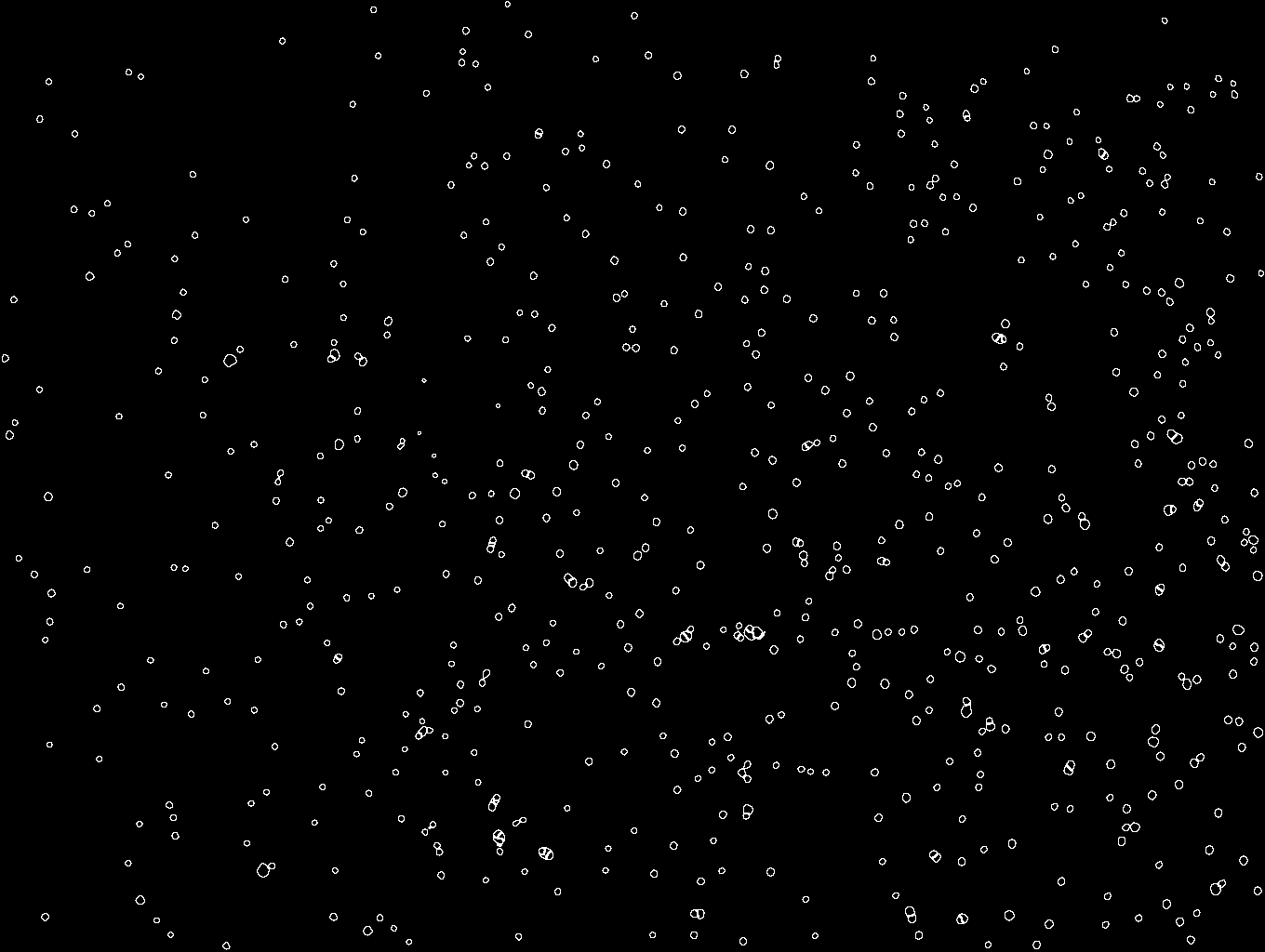

Supplement: Figure 2—figure supplement 2—source data 1. [file elife-38187-fig2-figsupp2-data1.zip › Figure 2 - figure suppl 2 - source data/Exp2/Plate B/Segmentation/r03c04f01p03-beads_outline.tif.tif]

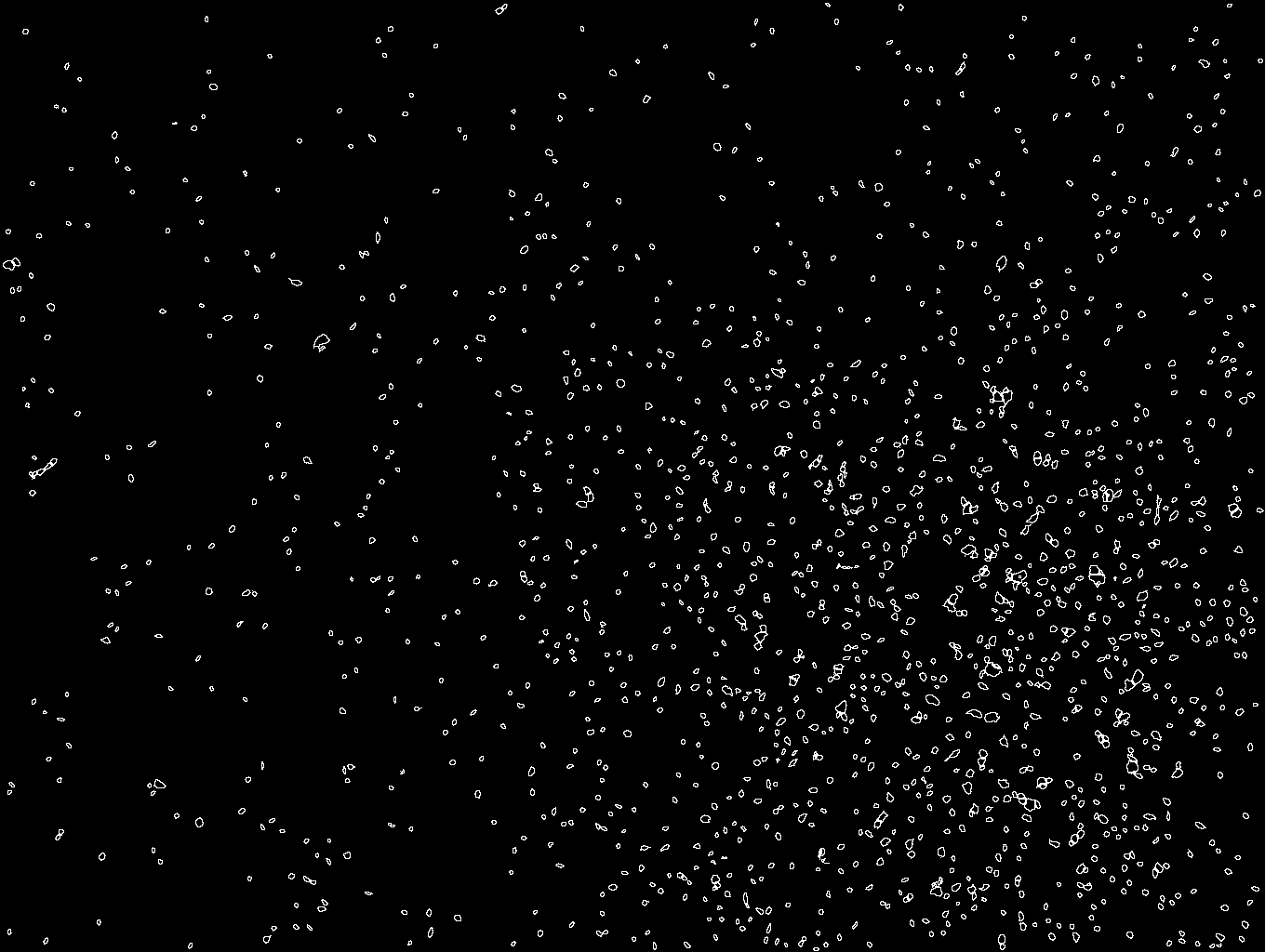

Supplement: Figure 2—figure supplement 2—source data 1. [file elife-38187-fig2-figsupp2-data1.zip › Figure 2 - figure suppl 2 - source data/Exp2/Plate B/Segmentation/r03c04f01p05-cell_outline.tif.tif]

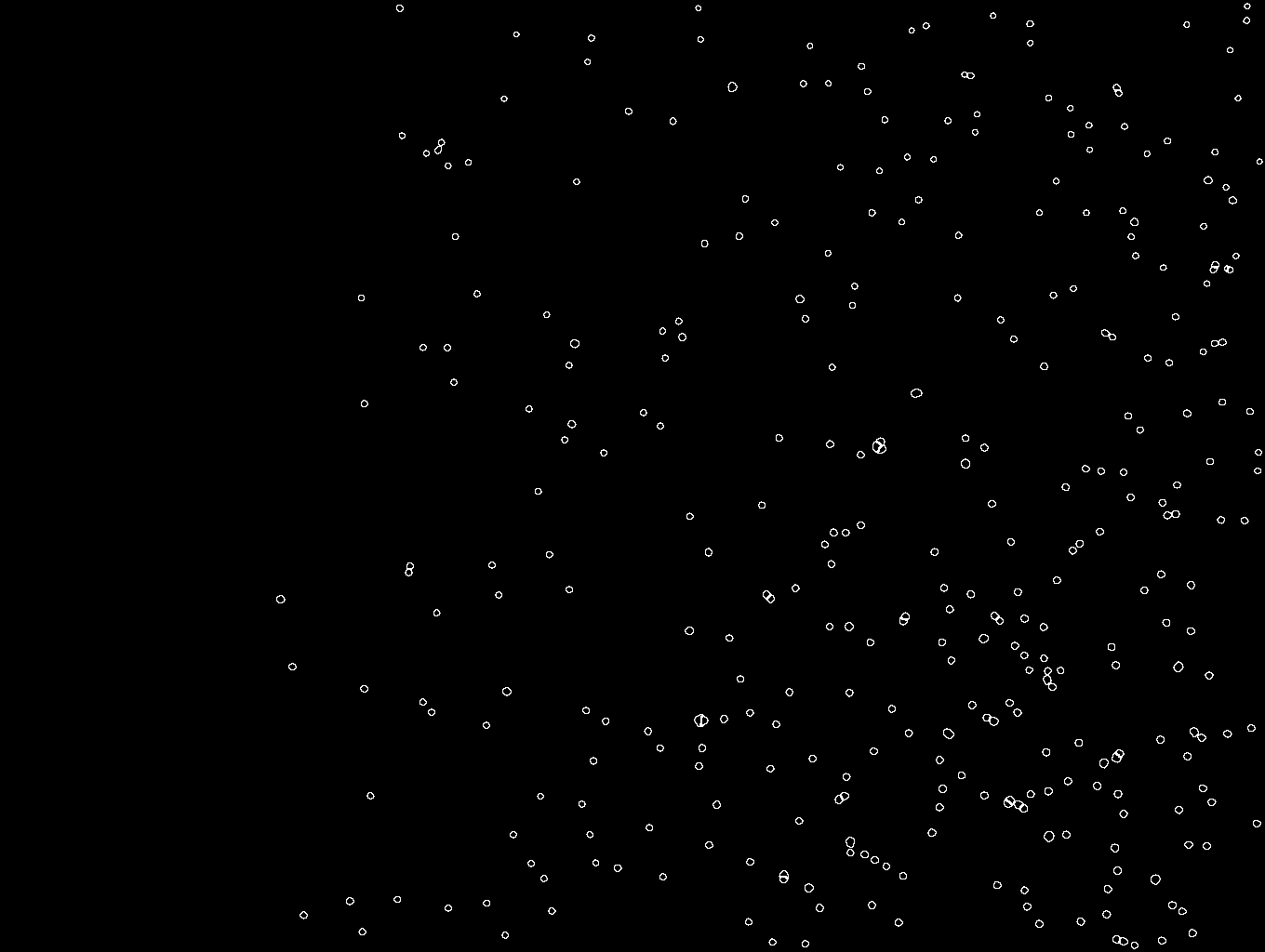

Supplement: Figure 2—figure supplement 2—source data 1. [file elife-38187-fig2-figsupp2-data1.zip › Figure 2 - figure suppl 2 - source data/Exp2/Plate B/Segmentation/r03c10f01p03-beads_outline.tif.tif]

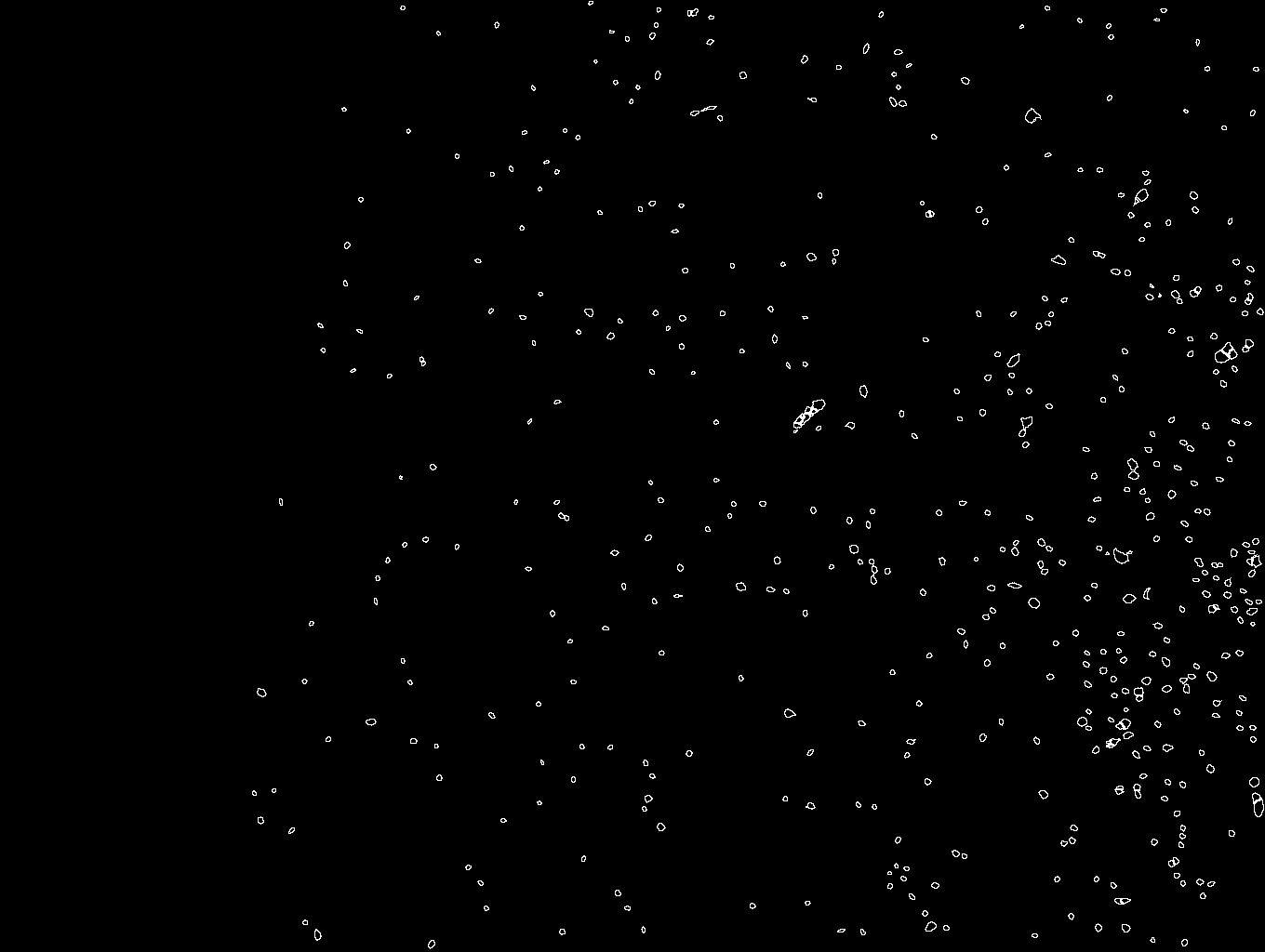

Supplement: Figure 2—figure supplement 2—source data 1. [file elife-38187-fig2-figsupp2-data1.zip › Figure 2 - figure suppl 2 - source data/Exp2/Plate B/Segmentation/r03c10f01p05-cell_outline.tif.tif]

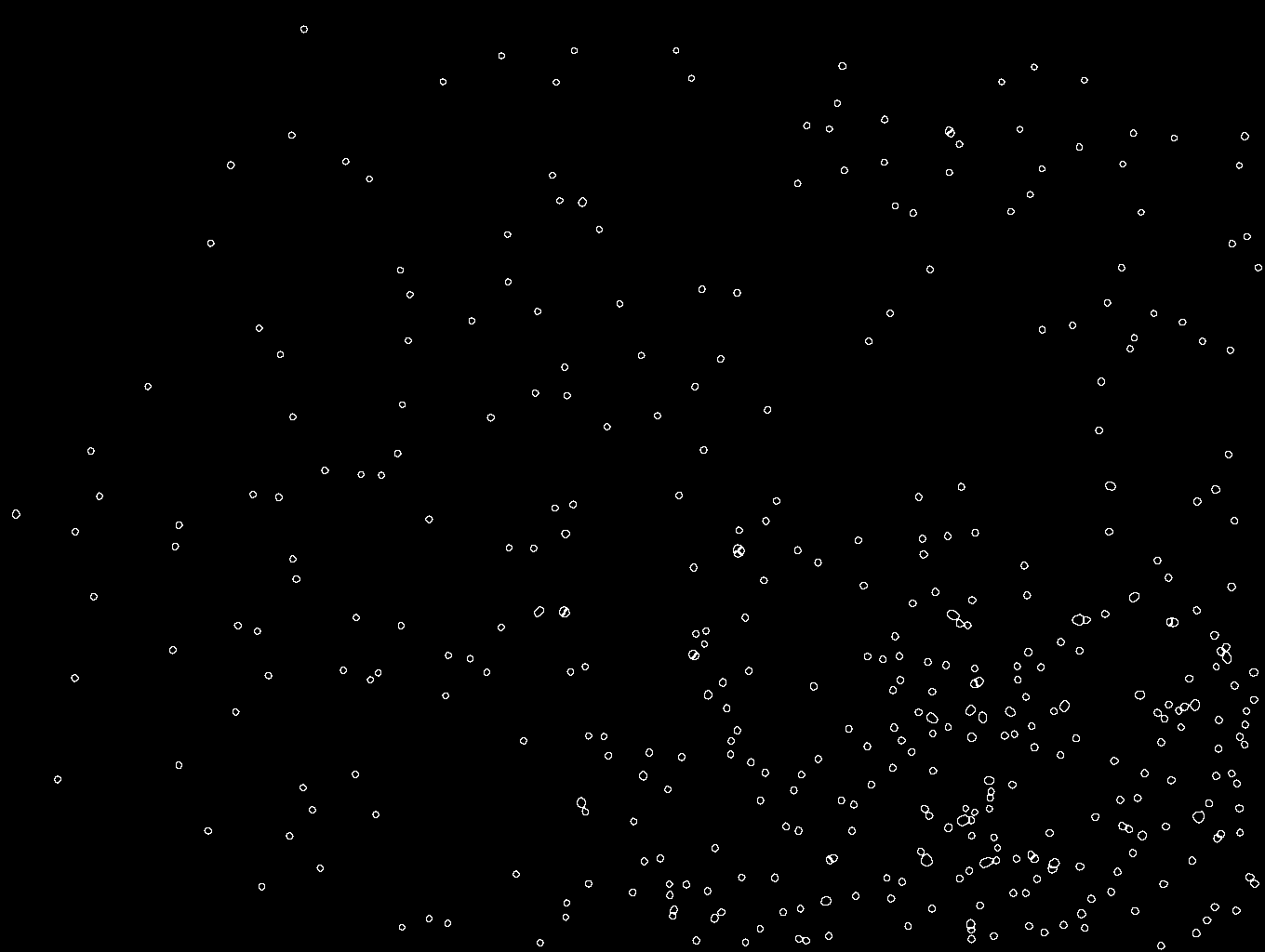

Supplement: Figure 2—figure supplement 2—source data 1. [file elife-38187-fig2-figsupp2-data1.zip › Figure 2 - figure suppl 2 - source data/Exp2/Plate B/Segmentation/r04c05f01p03-beads_outline.tif.tif]

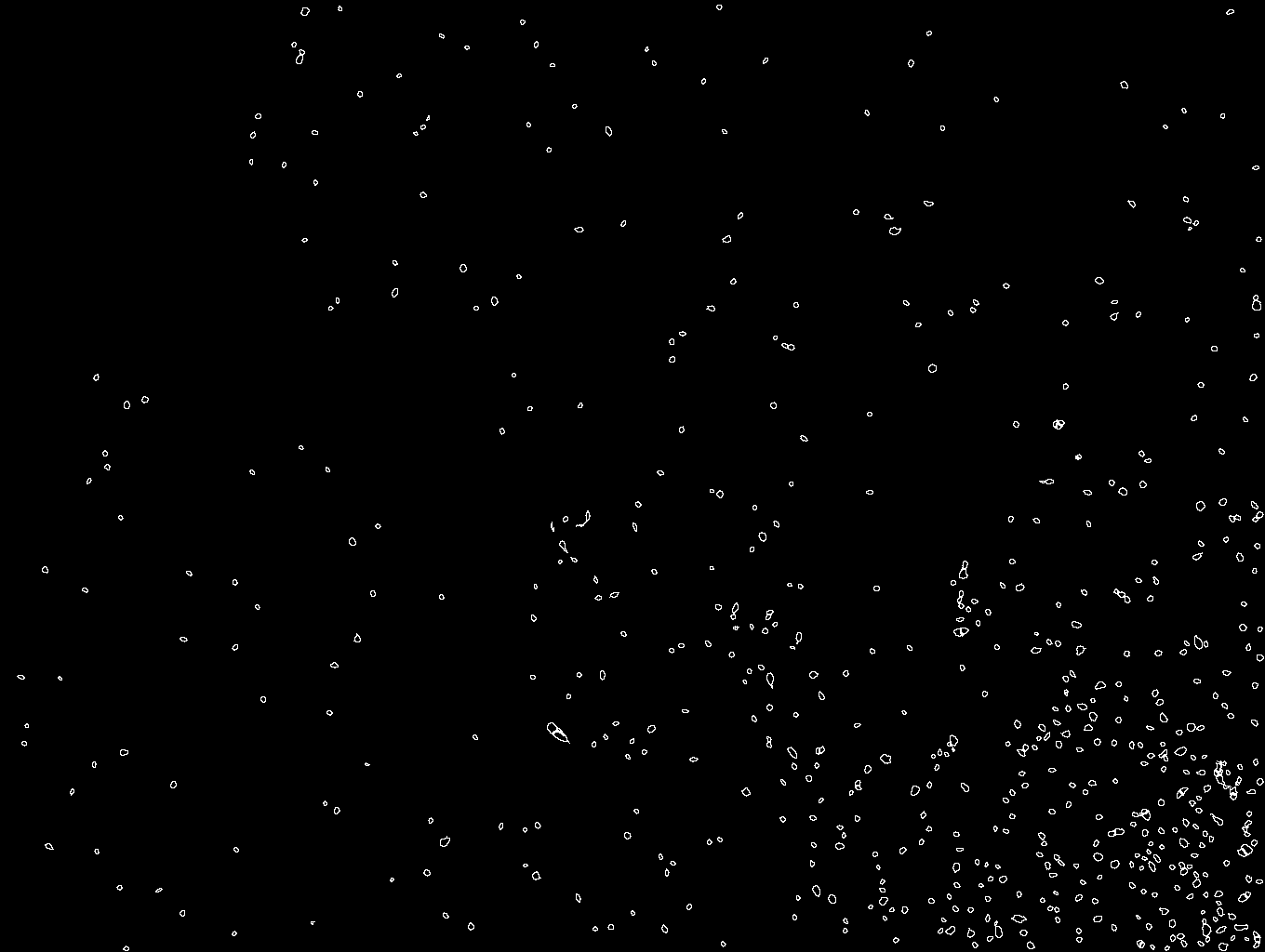

Supplement: Figure 2—figure supplement 2—source data 1. [file elife-38187-fig2-figsupp2-data1.zip › Figure 2 - figure suppl 2 - source data/Exp2/Plate B/Segmentation/r04c05f01p05-cell_outline.tif.tif]

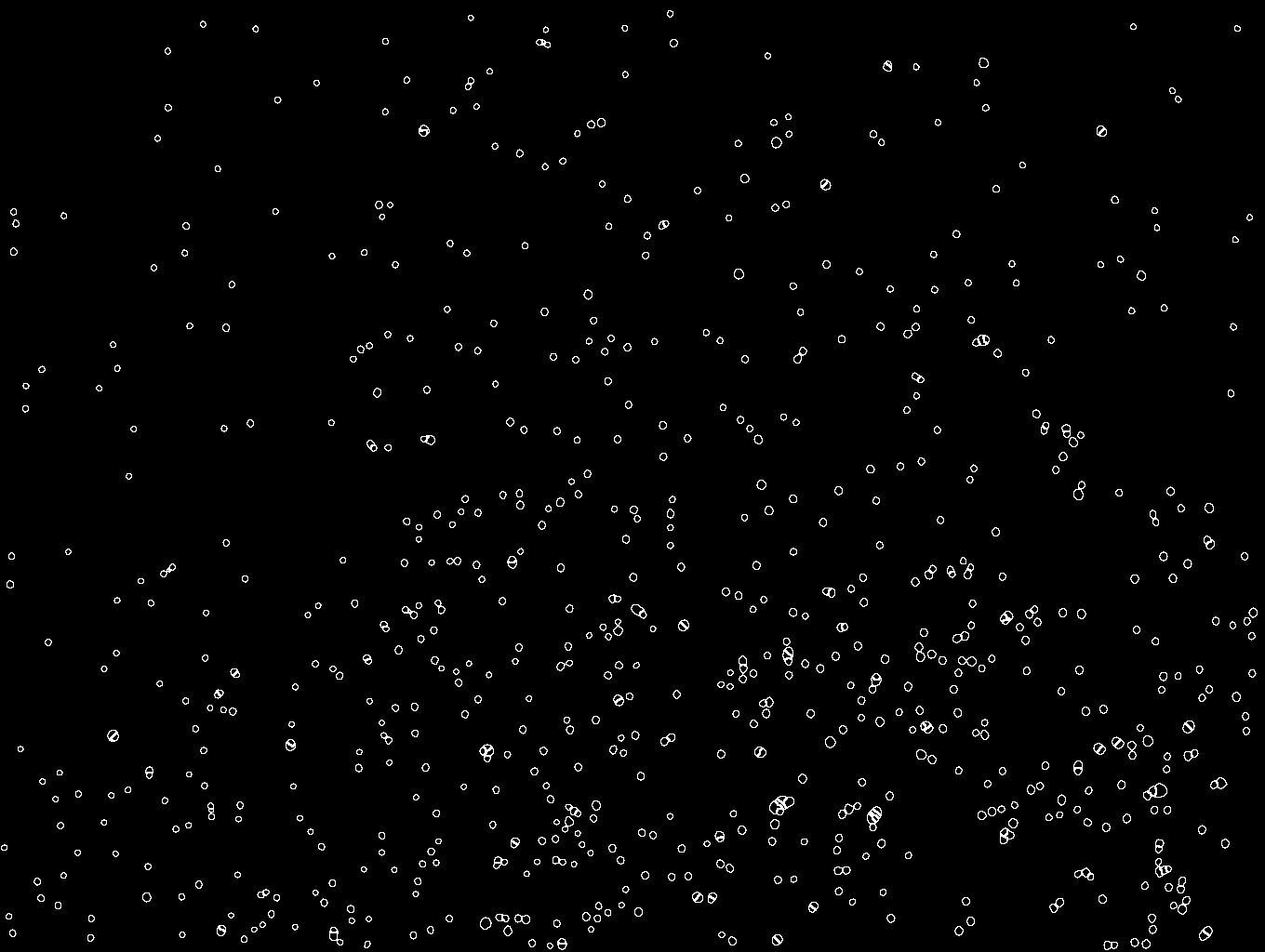

Supplement: Figure 2—figure supplement 2—source data 1. [file elife-38187-fig2-figsupp2-data1.zip › Figure 2 - figure suppl 2 - source data/Exp2/Plate B/Segmentation/r04c06f01p03-beads_outline.tif.tif]

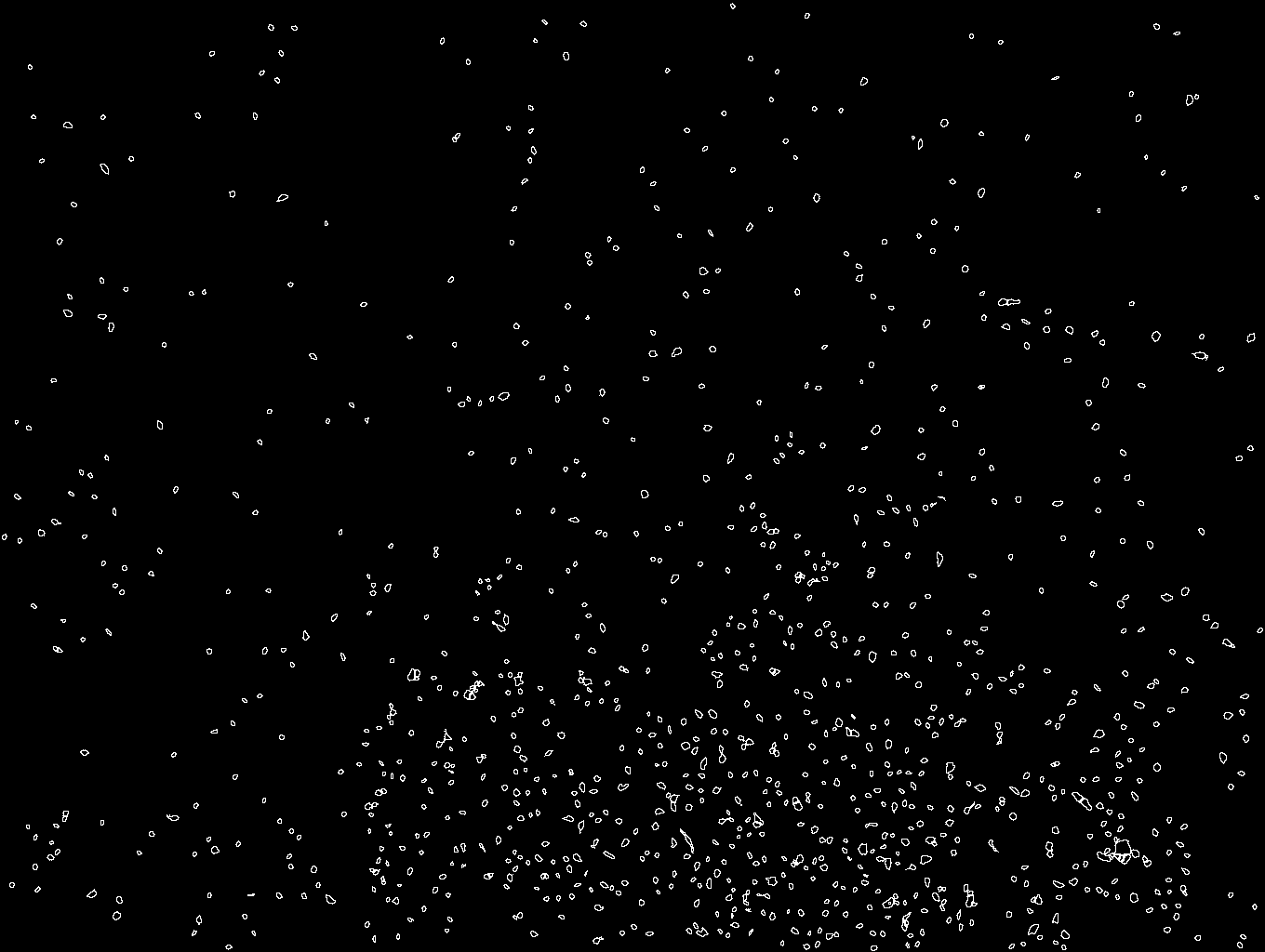

Supplement: Figure 2—figure supplement 2—source data 1. [file elife-38187-fig2-figsupp2-data1.zip › Figure 2 - figure suppl 2 - source data/Exp2/Plate B/Segmentation/r04c06f01p05-cell_outline.tif.tif]

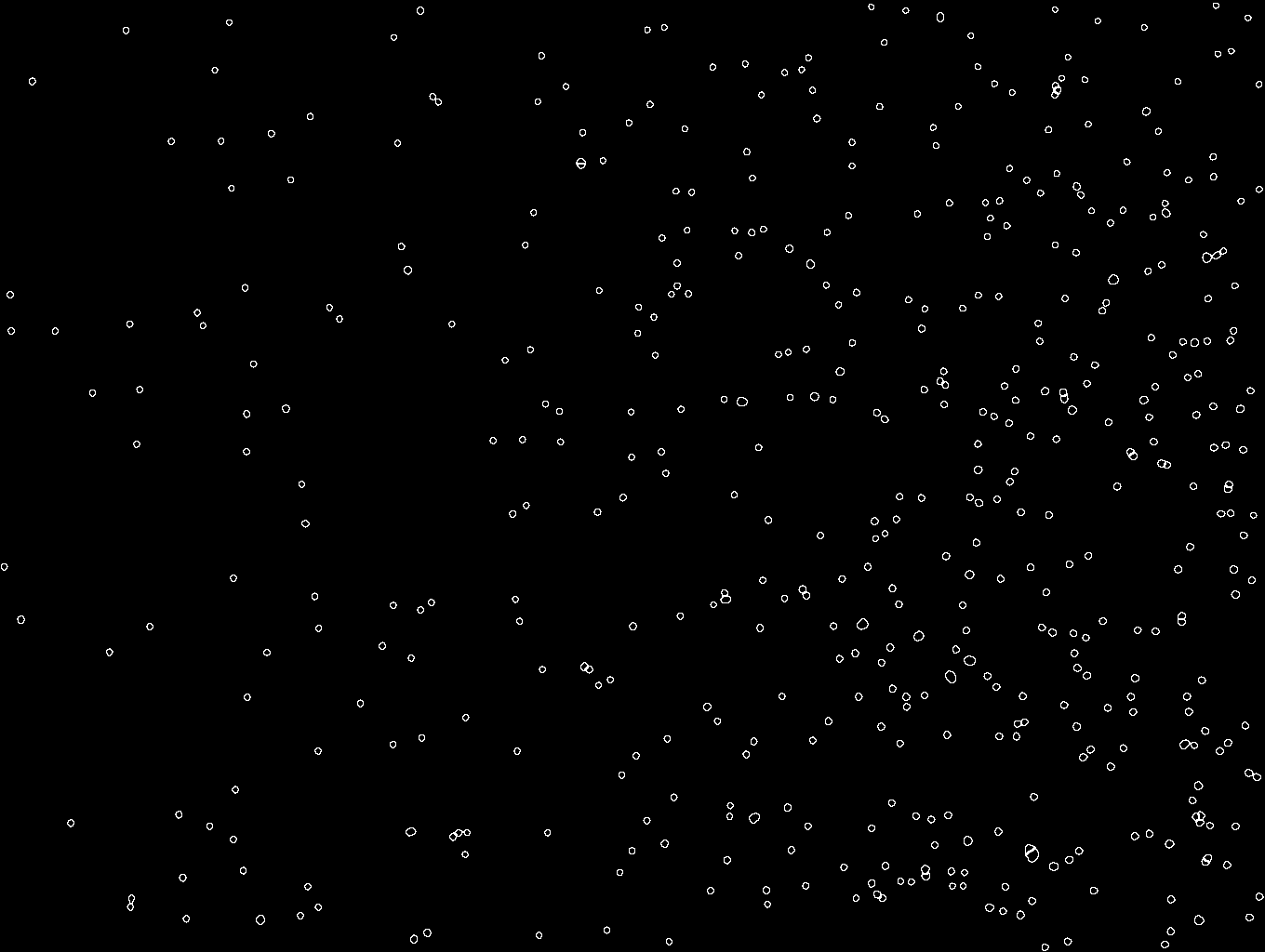

Supplement: Figure 2—figure supplement 2—source data 1. [file elife-38187-fig2-figsupp2-data1.zip › Figure 2 - figure suppl 2 - source data/Exp2/Plate B/Segmentation/r05c06f01p03-beads_outline.tif.tif]

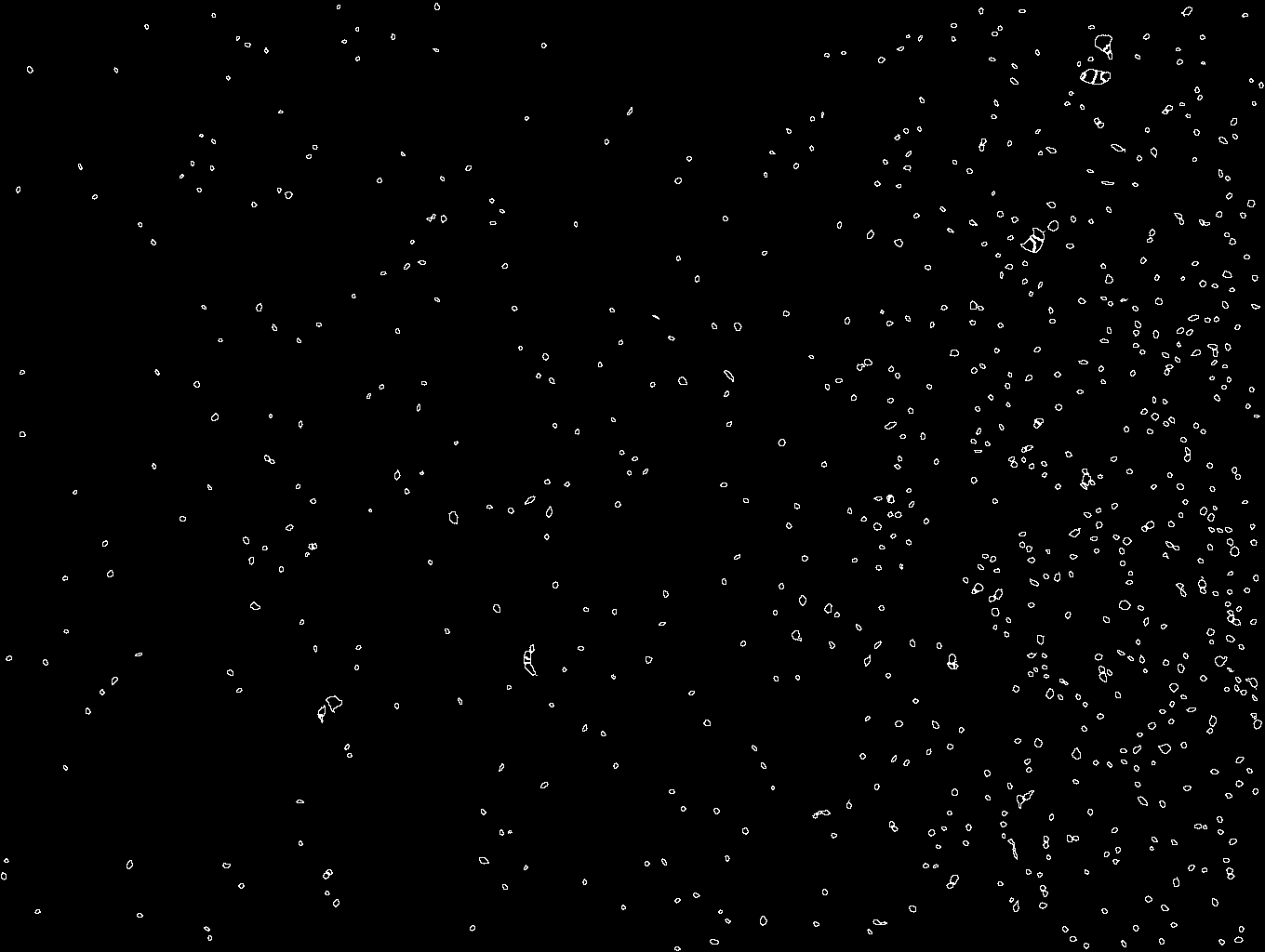

Supplement: Figure 2—figure supplement 2—source data 1. [file elife-38187-fig2-figsupp2-data1.zip › Figure 2 - figure suppl 2 - source data/Exp2/Plate B/Segmentation/r05c06f01p05-cell_outline.tif.tif]

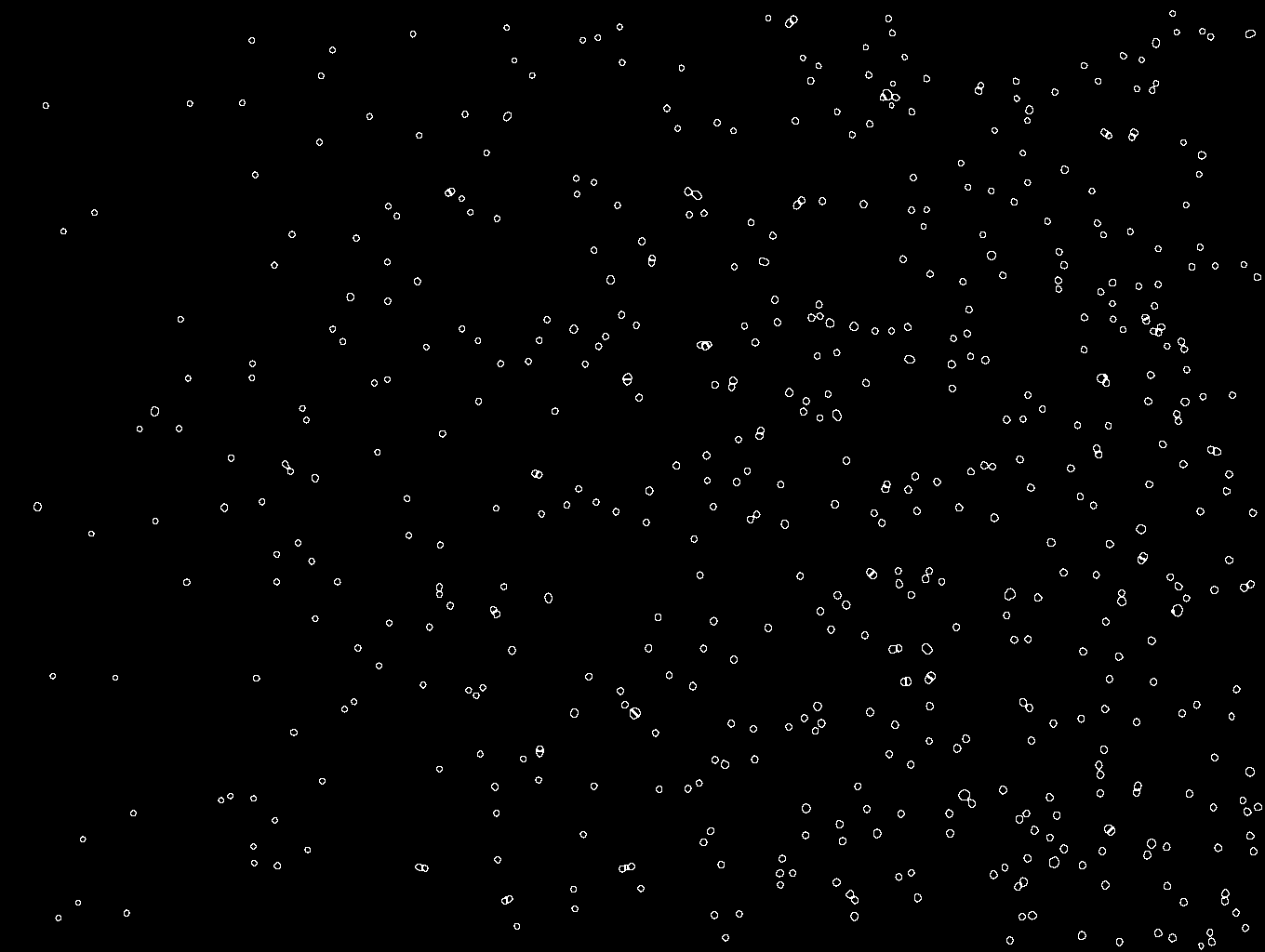

Supplement: Figure 2—figure supplement 2—source data 1. [file elife-38187-fig2-figsupp2-data1.zip › Figure 2 - figure suppl 2 - source data/Exp2/Plate B/Segmentation/r05c09f01p03-beads_outline.tif.tif]

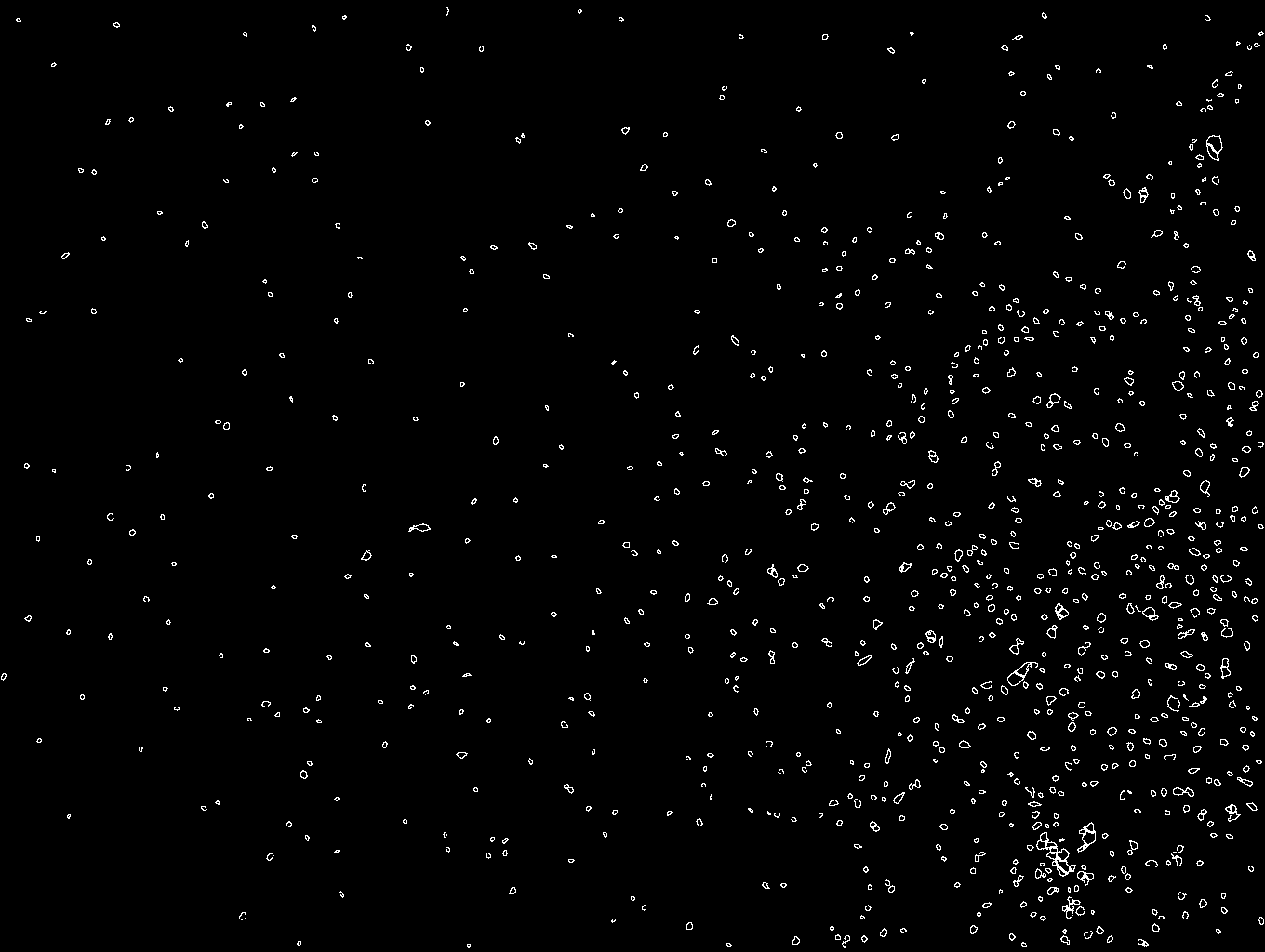

Supplement: Figure 2—figure supplement 2—source data 1. [file elife-38187-fig2-figsupp2-data1.zip › Figure 2 - figure suppl 2 - source data/Exp2/Plate B/Segmentation/r05c09f01p05-cell_outline.tif.tif]

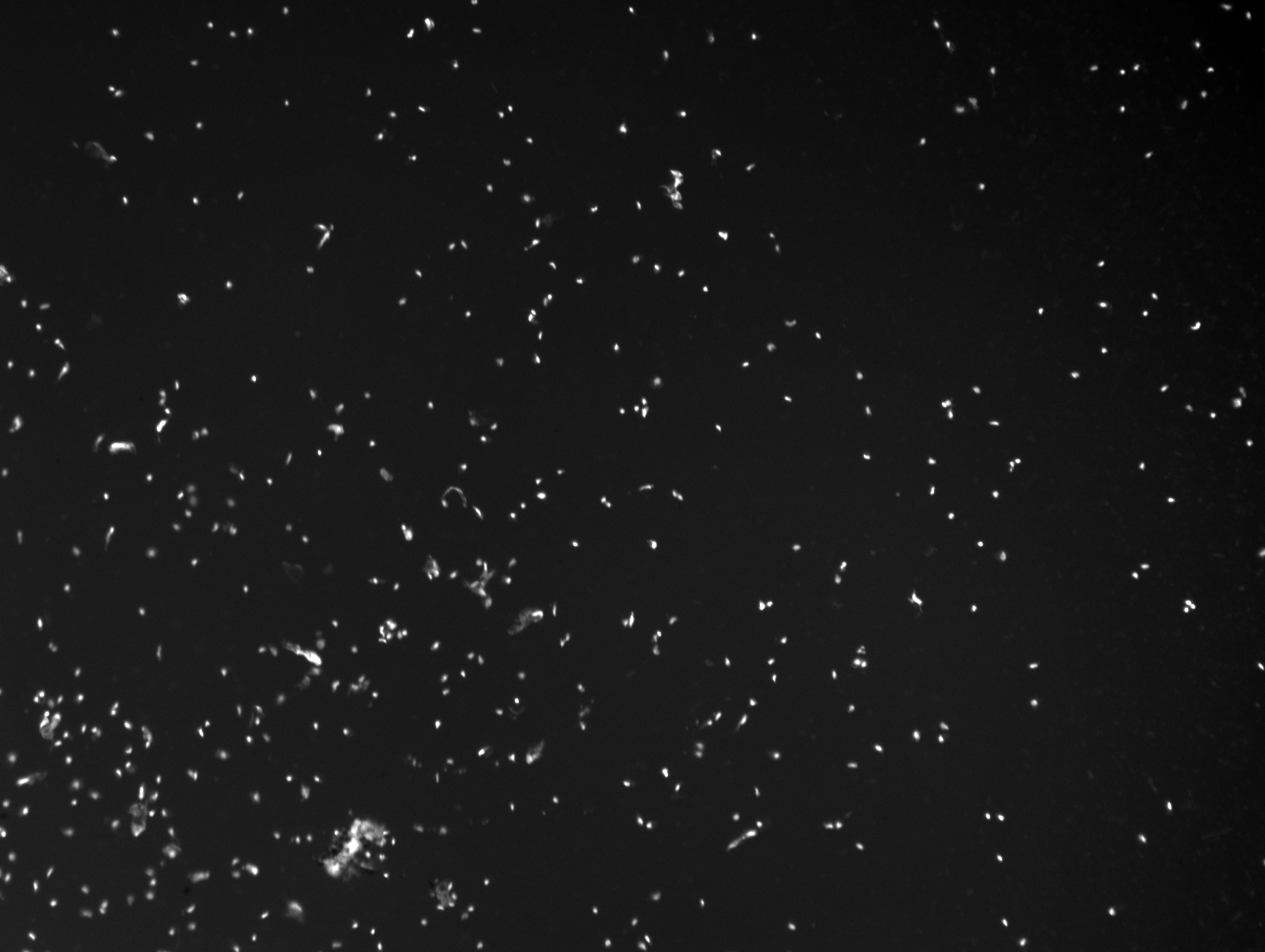

Supplement: Figure 2—figure supplement 2—source data 1. [file elife-38187-fig2-figsupp2-data1.zip › Figure 2 - figure suppl 2 - source data/Exp4/Plate A/r02c02f01p01-ch1sk1fk1fl1.tiff]

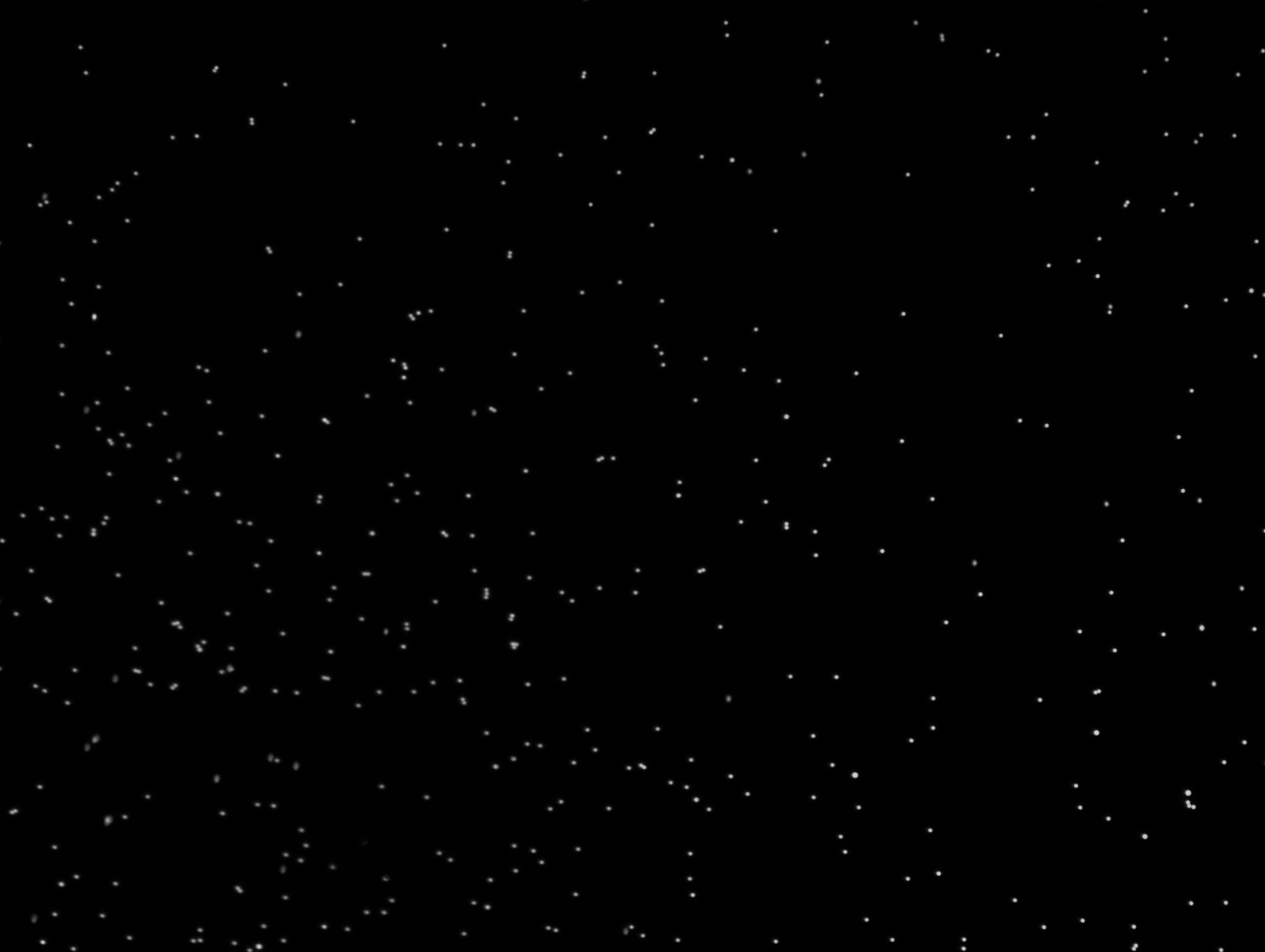

Supplement: Figure 2—figure supplement 2—source data 1. [file elife-38187-fig2-figsupp2-data1.zip › Figure 2 - figure suppl 2 - source data/Exp4/Plate A/r02c02f01p01-ch2sk1fk1fl1.tiff]

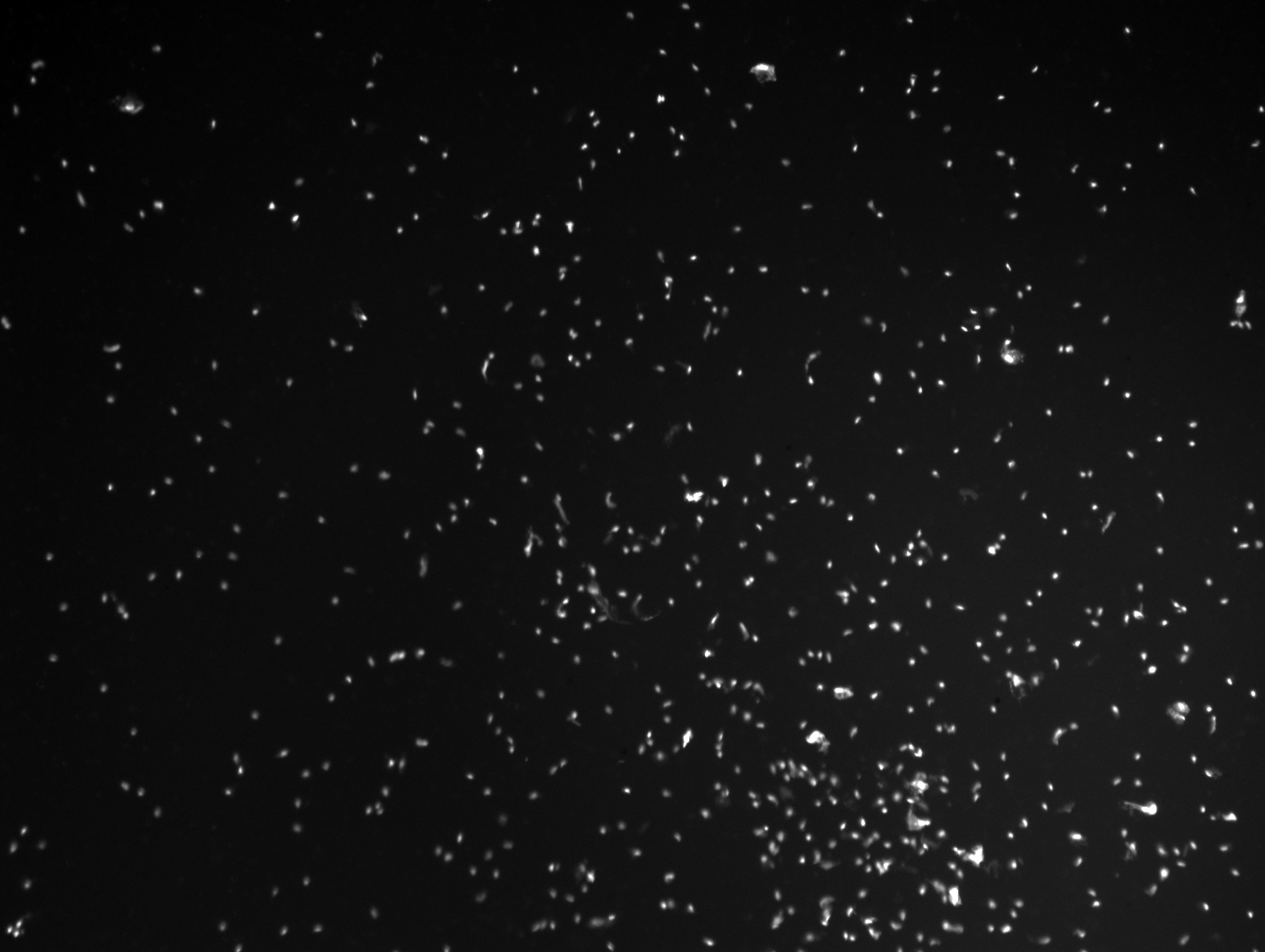

Supplement: Figure 2—figure supplement 2—source data 1. [file elife-38187-fig2-figsupp2-data1.zip › Figure 2 - figure suppl 2 - source data/Exp4/Plate A/r02c05f01p01-ch1sk1fk1fl1.tiff]

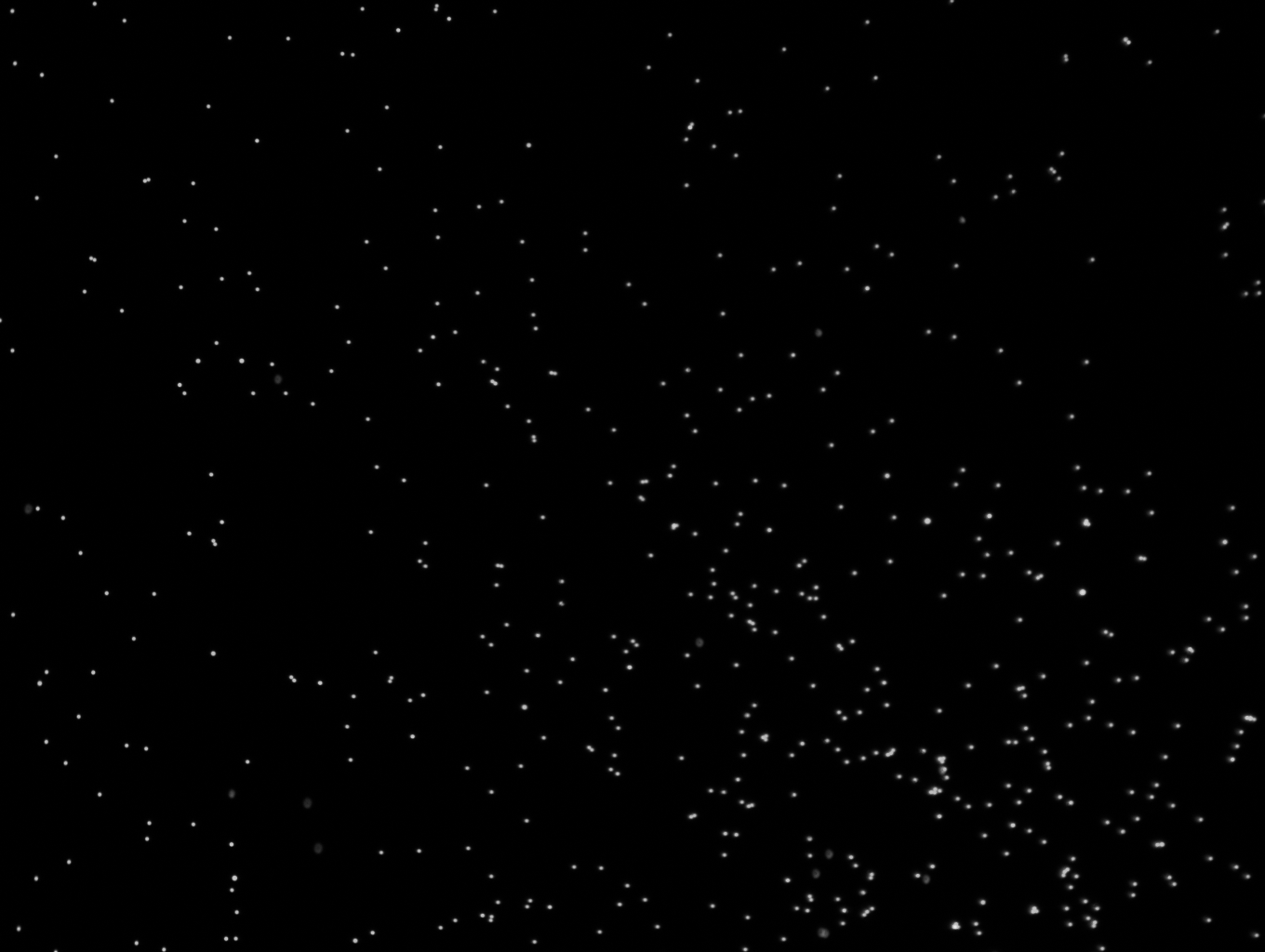

Supplement: Figure 2—figure supplement 2—source data 1. [file elife-38187-fig2-figsupp2-data1.zip › Figure 2 - figure suppl 2 - source data/Exp4/Plate A/r02c05f01p01-ch2sk1fk1fl1.tiff]

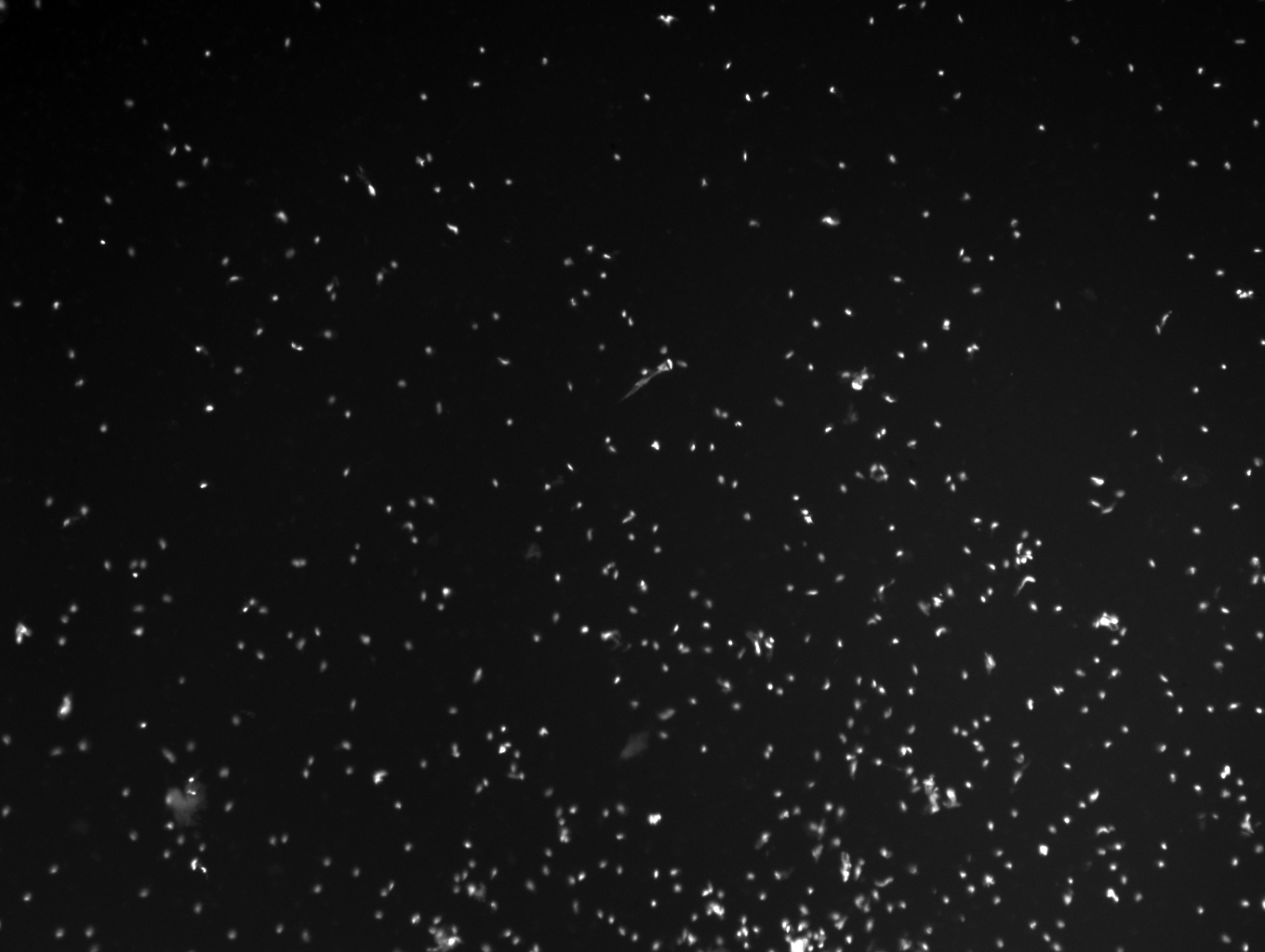

Supplement: Figure 2—figure supplement 2—source data 1. [file elife-38187-fig2-figsupp2-data1.zip › Figure 2 - figure suppl 2 - source data/Exp4/Plate A/r02c09f01p01-ch1sk1fk1fl1.tiff]

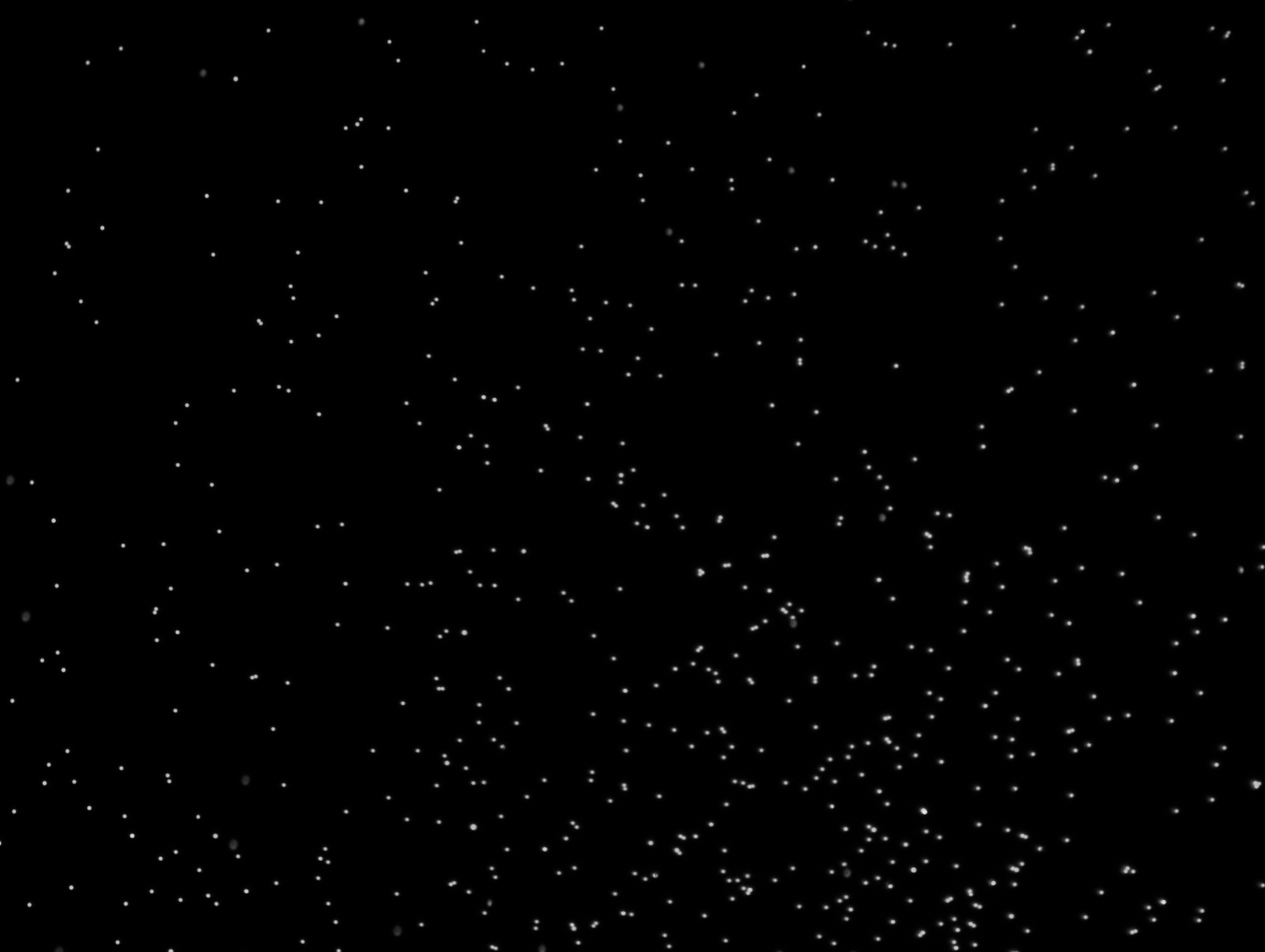

Supplement: Figure 2—figure supplement 2—source data 1. [file elife-38187-fig2-figsupp2-data1.zip › Figure 2 - figure suppl 2 - source data/Exp4/Plate A/r02c09f01p01-ch2sk1fk1fl1.tiff]

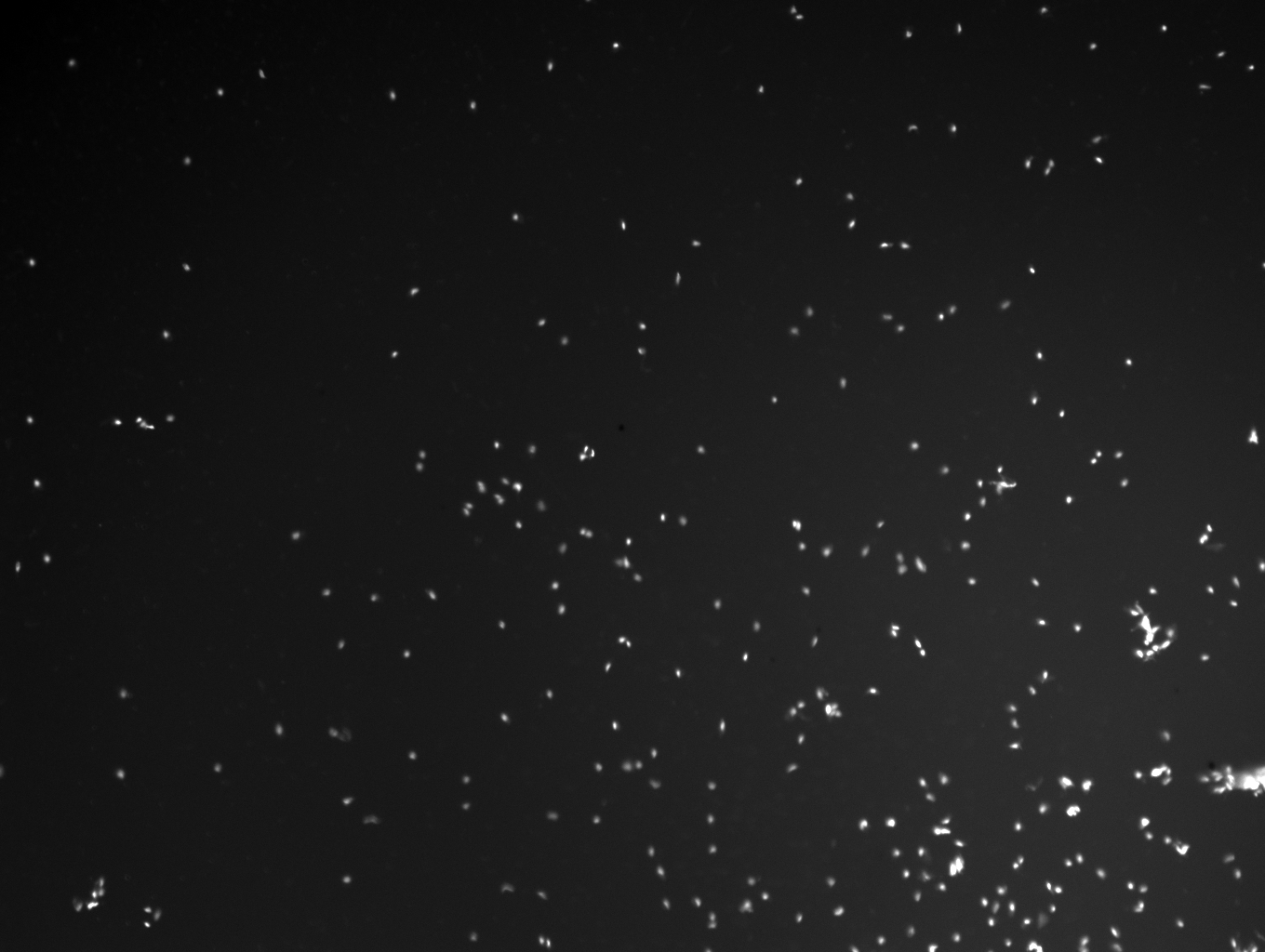

Supplement: Figure 2—figure supplement 2—source data 1. [file elife-38187-fig2-figsupp2-data1.zip › Figure 2 - figure suppl 2 - source data/Exp4/Plate A/r03c04f01p01-ch1sk1fk1fl1.tiff]

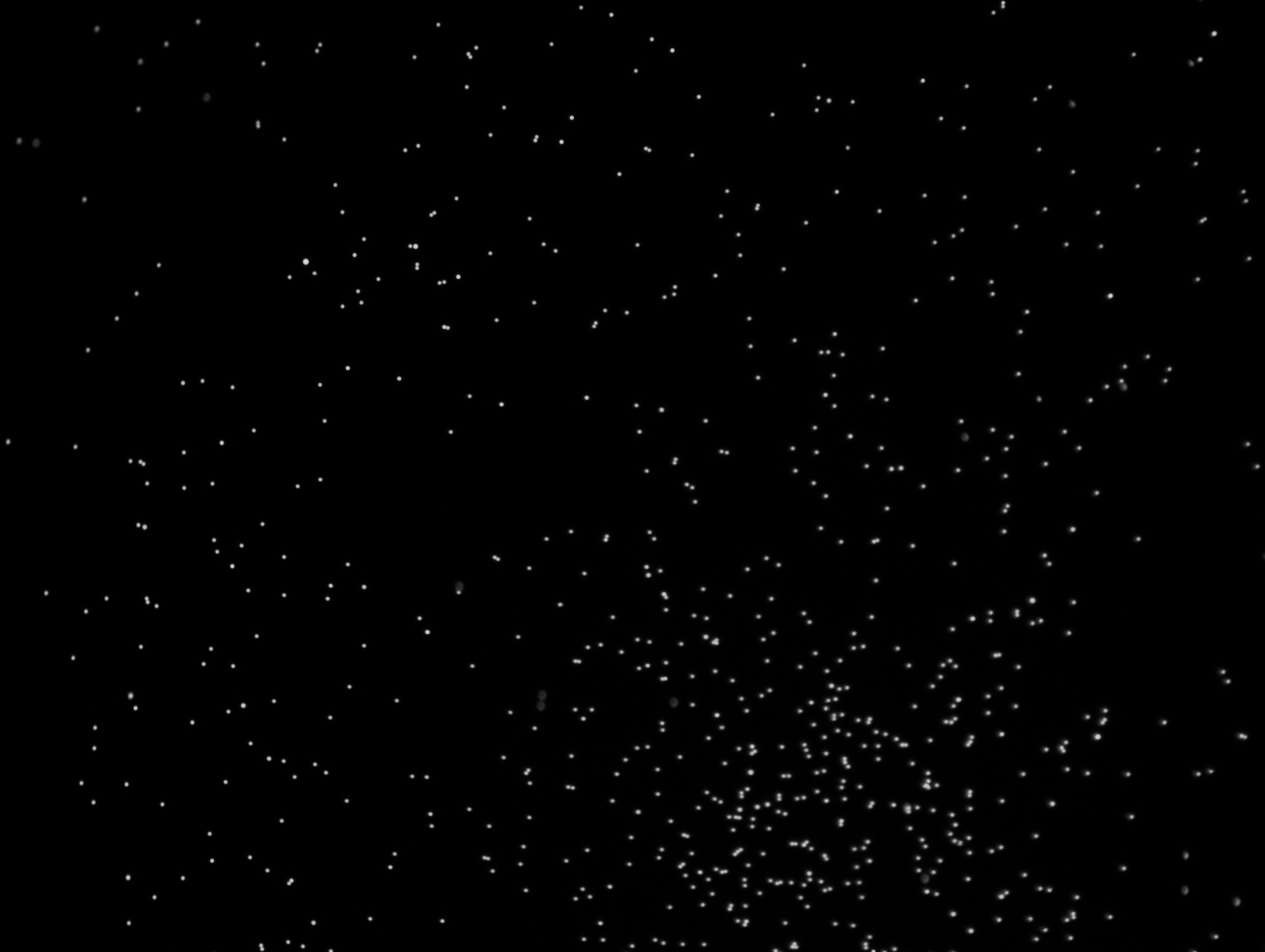

Supplement: Figure 2—figure supplement 2—source data 1. [file elife-38187-fig2-figsupp2-data1.zip › Figure 2 - figure suppl 2 - source data/Exp4/Plate A/r03c04f01p01-ch2sk1fk1fl1.tiff]

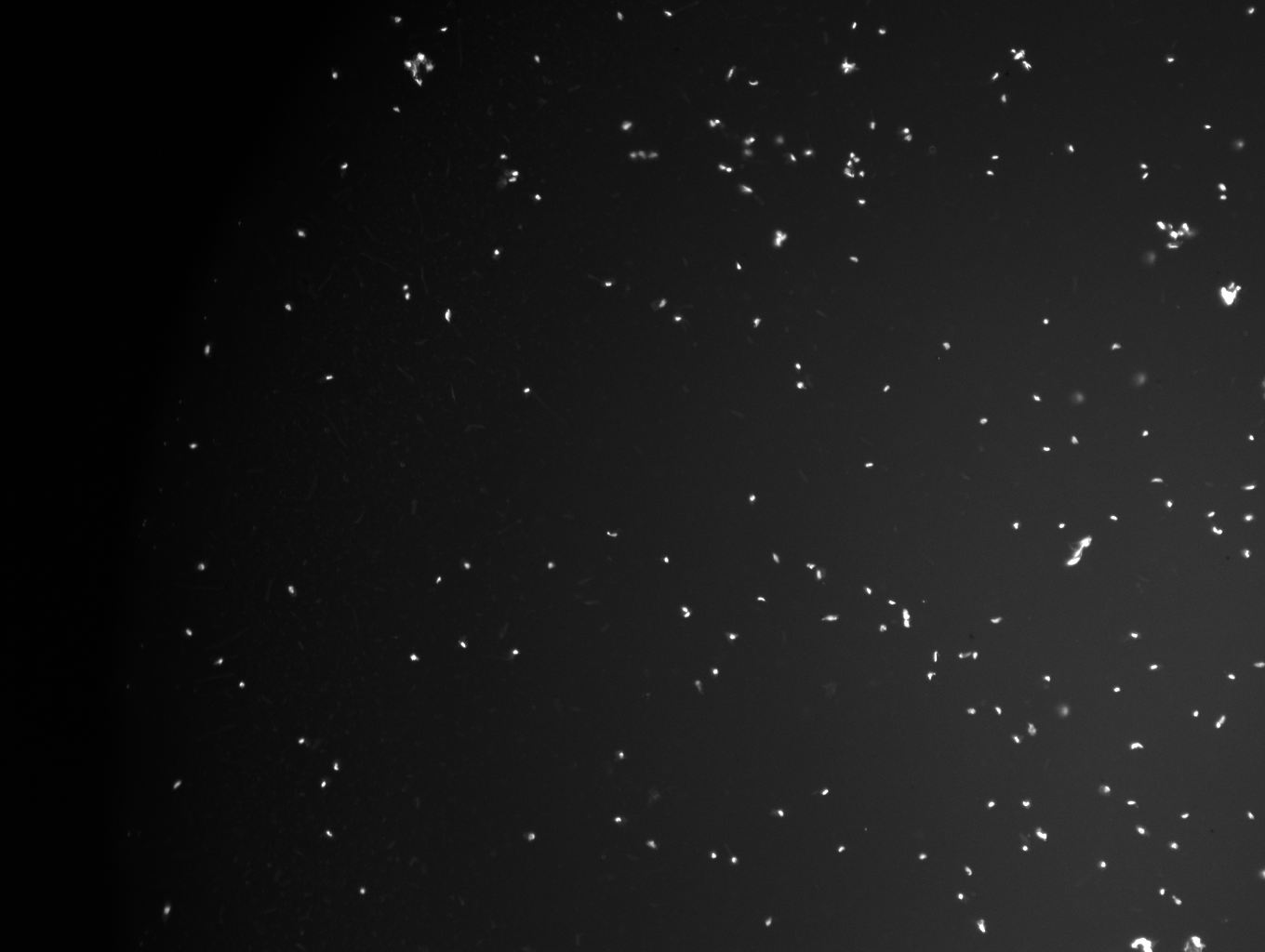

Supplement: Figure 2—figure supplement 2—source data 1. [file elife-38187-fig2-figsupp2-data1.zip › Figure 2 - figure suppl 2 - source data/Exp4/Plate A/r03c09f01p01-ch1sk1fk1fl1.tiff]

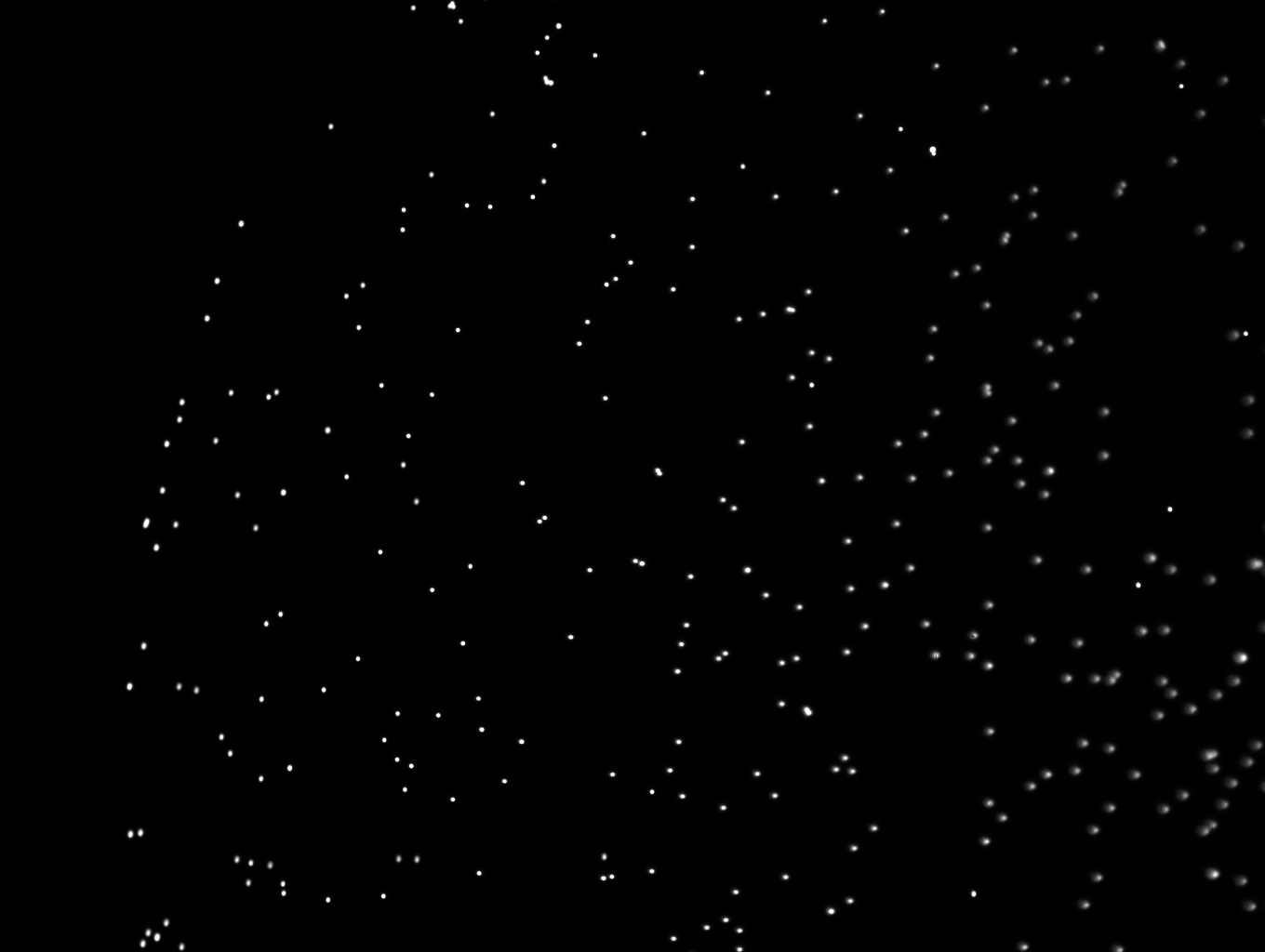

Supplement: Figure 2—figure supplement 2—source data 1. [file elife-38187-fig2-figsupp2-data1.zip › Figure 2 - figure suppl 2 - source data/Exp4/Plate A/r03c09f01p01-ch2sk1fk1fl1.tiff]

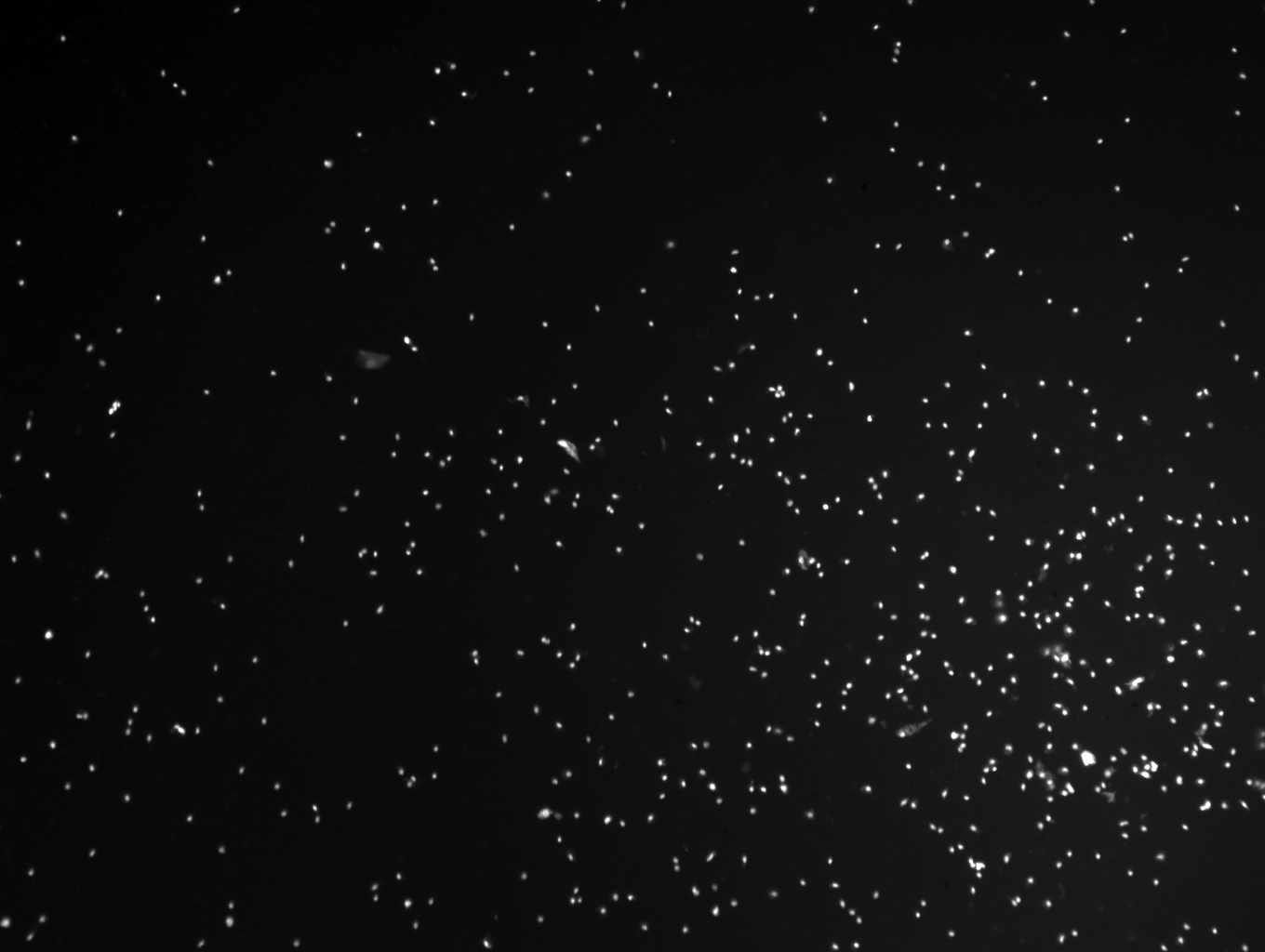

Supplement: Figure 2—figure supplement 2—source data 1. [file elife-38187-fig2-figsupp2-data1.zip › Figure 2 - figure suppl 2 - source data/Exp4/Plate A/r04c02f01p01-ch1sk1fk1fl1.tiff]

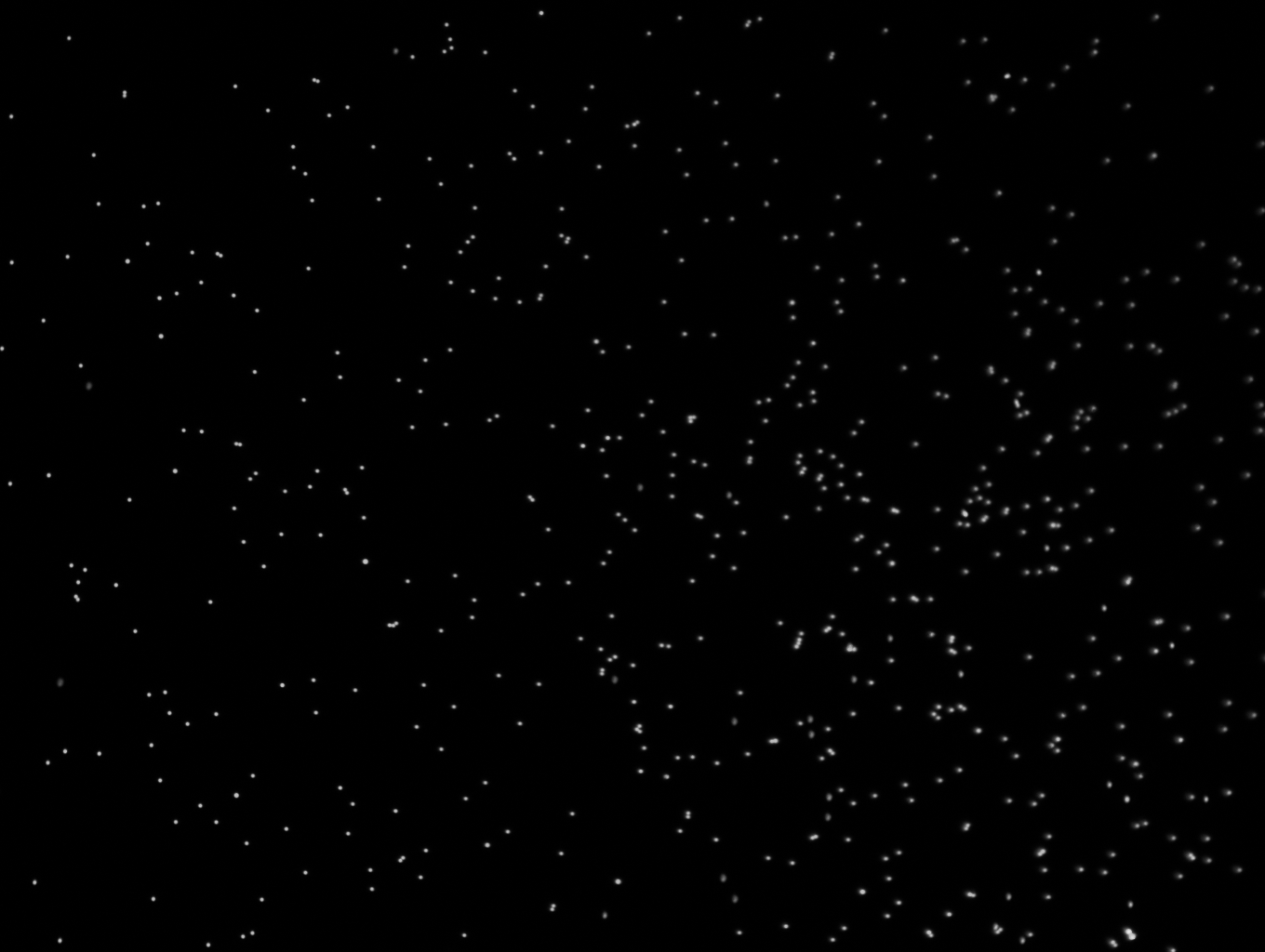

Supplement: Figure 2—figure supplement 2—source data 1. [file elife-38187-fig2-figsupp2-data1.zip › Figure 2 - figure suppl 2 - source data/Exp4/Plate A/r04c02f01p01-ch2sk1fk1fl1.tiff]

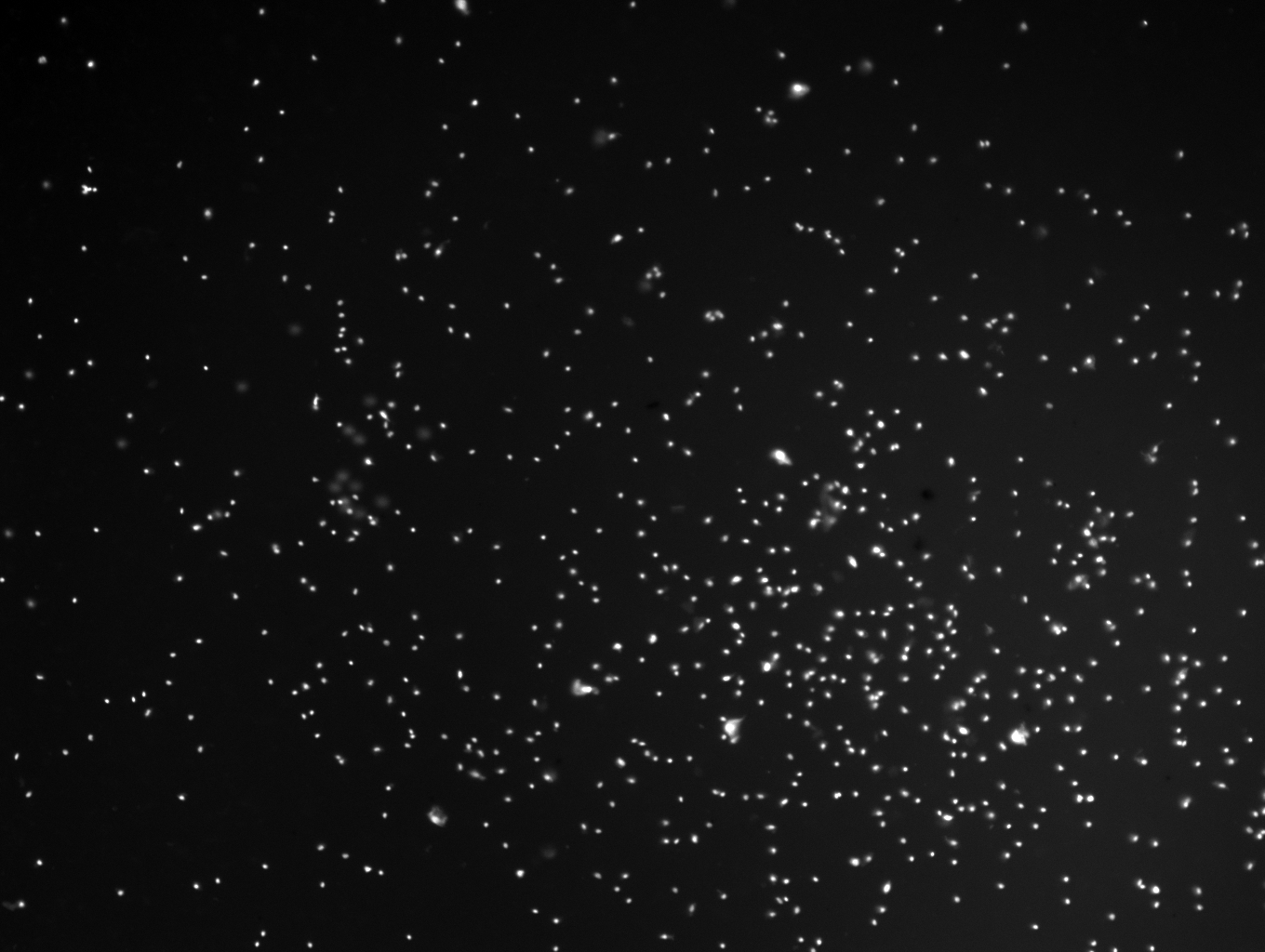

Supplement: Figure 2—figure supplement 2—source data 1. [file elife-38187-fig2-figsupp2-data1.zip › Figure 2 - figure suppl 2 - source data/Exp4/Plate A/r04c03f01p01-ch1sk1fk1fl1.tiff]

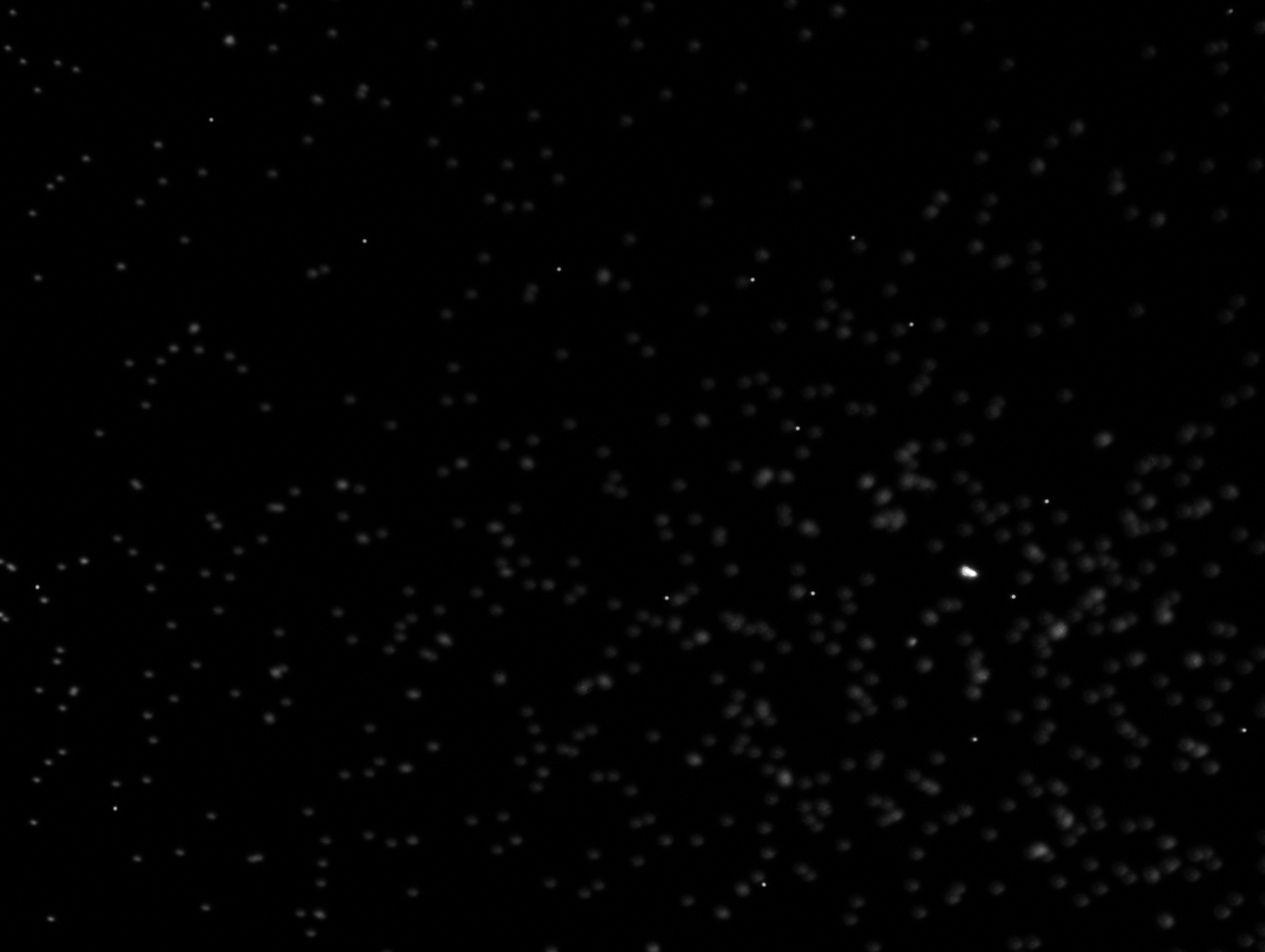

Supplement: Figure 2—figure supplement 2—source data 1. [file elife-38187-fig2-figsupp2-data1.zip › Figure 2 - figure suppl 2 - source data/Exp4/Plate A/r04c03f01p01-ch2sk1fk1fl1.tiff]

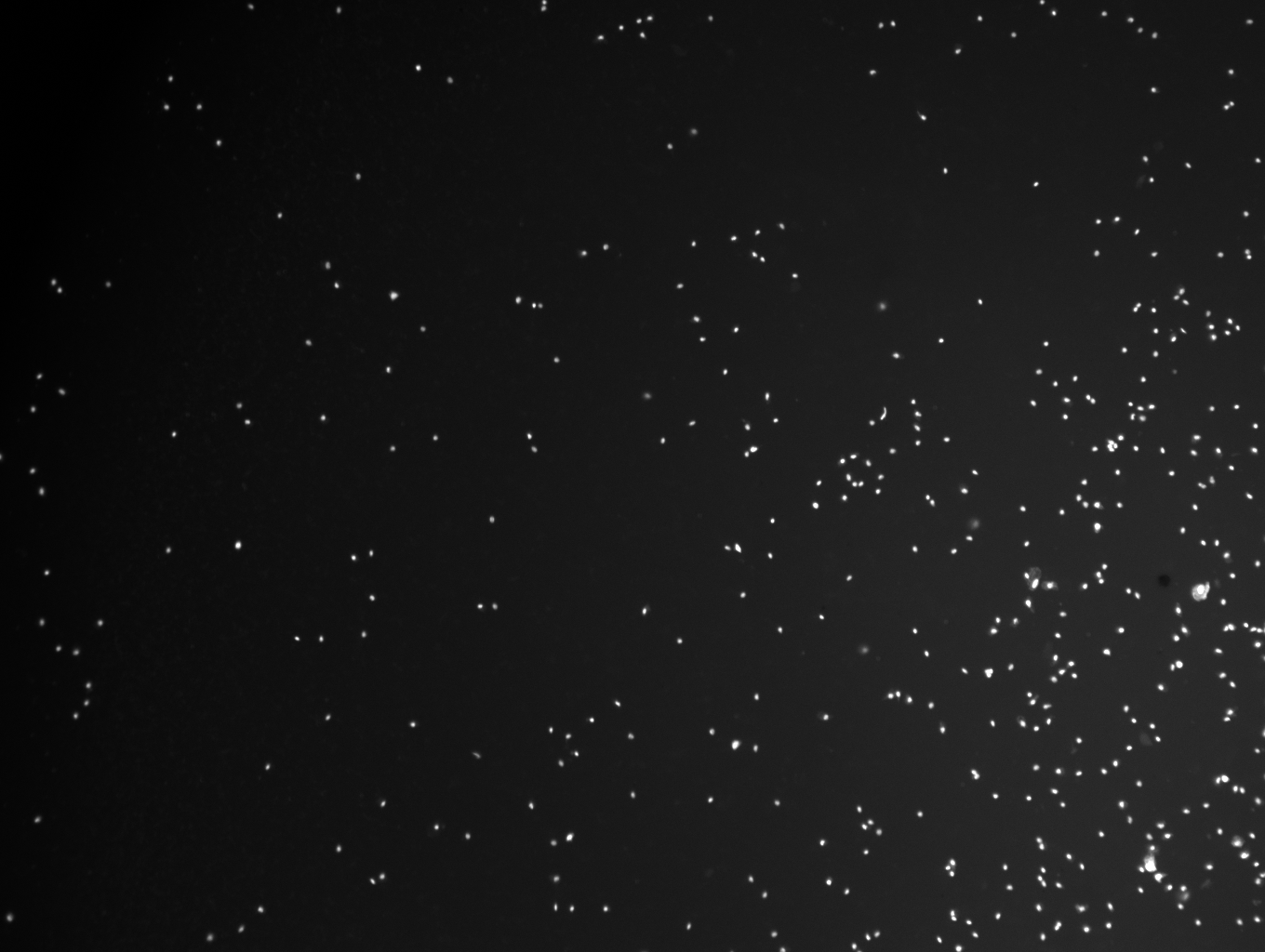

Supplement: Figure 2—figure supplement 2—source data 1. [file elife-38187-fig2-figsupp2-data1.zip › Figure 2 - figure suppl 2 - source data/Exp4/Plate A/r04c07f01p01-ch1sk1fk1fl1.tiff]

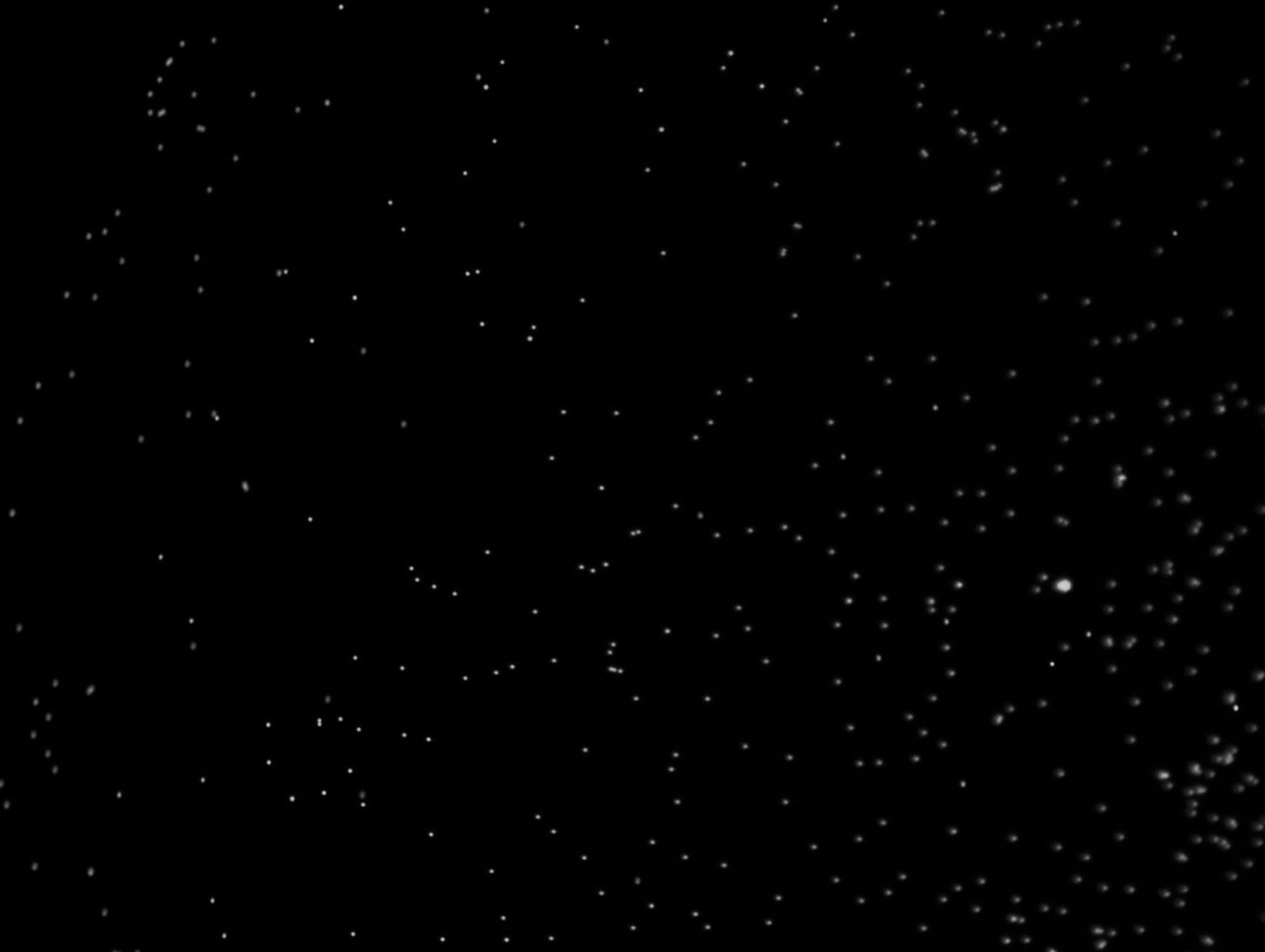

Supplement: Figure 2—figure supplement 2—source data 1. [file elife-38187-fig2-figsupp2-data1.zip › Figure 2 - figure suppl 2 - source data/Exp4/Plate A/r04c07f01p01-ch2sk1fk1fl1.tiff]

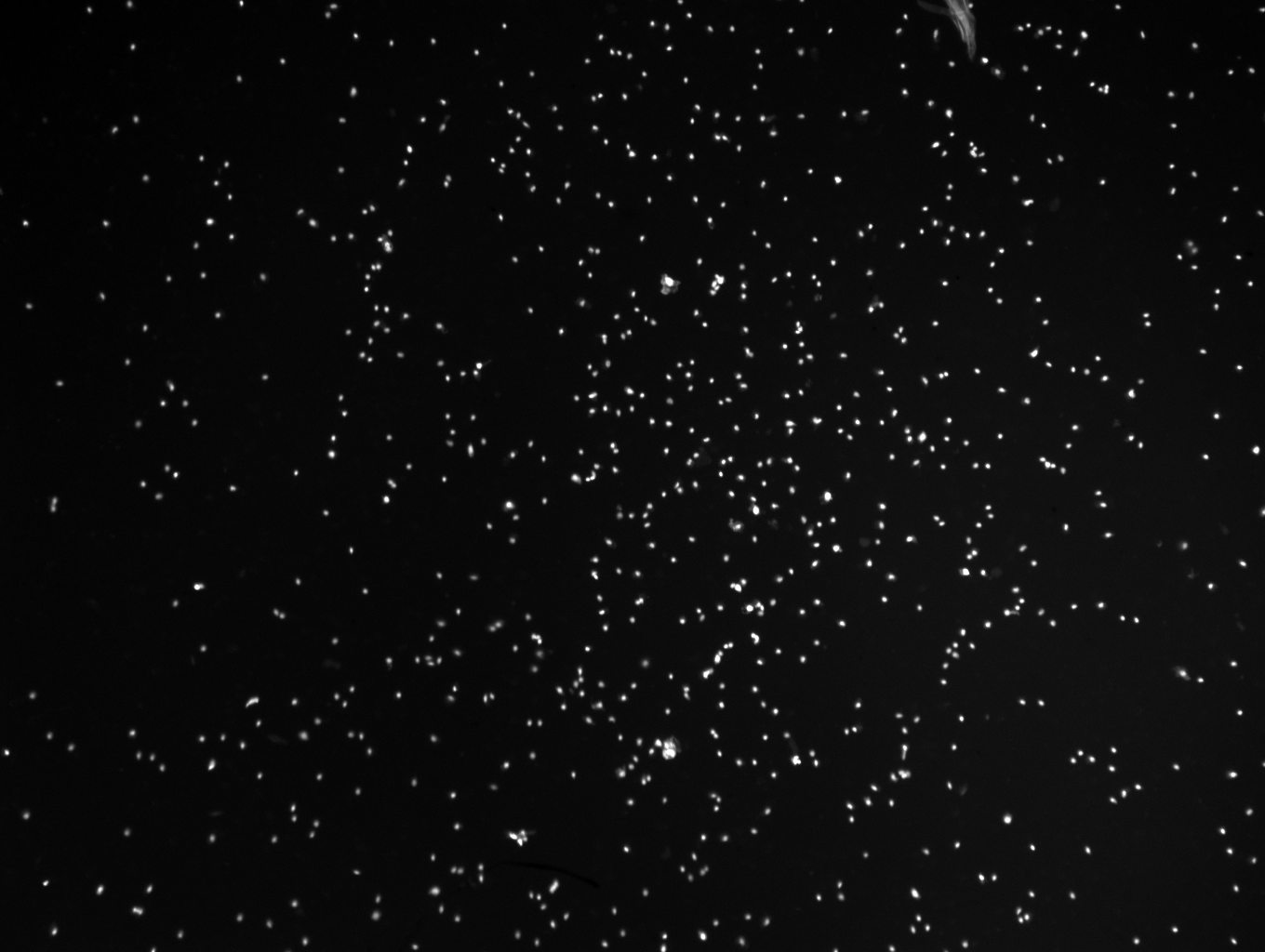

Supplement: Figure 2—figure supplement 2—source data 1. [file elife-38187-fig2-figsupp2-data1.zip › Figure 2 - figure suppl 2 - source data/Exp4/Plate A/r04c08f01p01-ch1sk1fk1fl1.tiff]

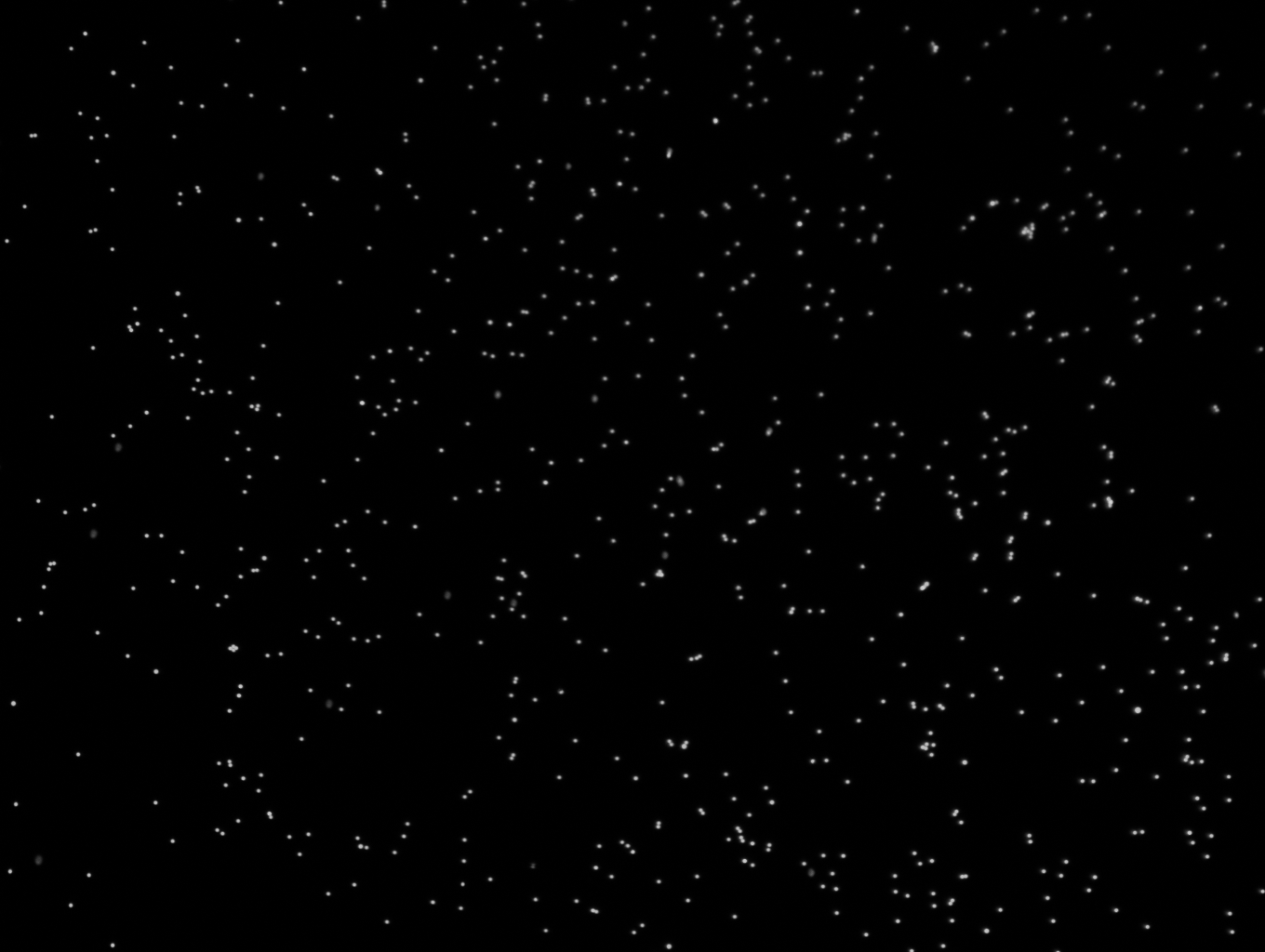

Supplement: Figure 2—figure supplement 2—source data 1. [file elife-38187-fig2-figsupp2-data1.zip › Figure 2 - figure suppl 2 - source data/Exp4/Plate A/r04c08f01p01-ch2sk1fk1fl1.tiff]

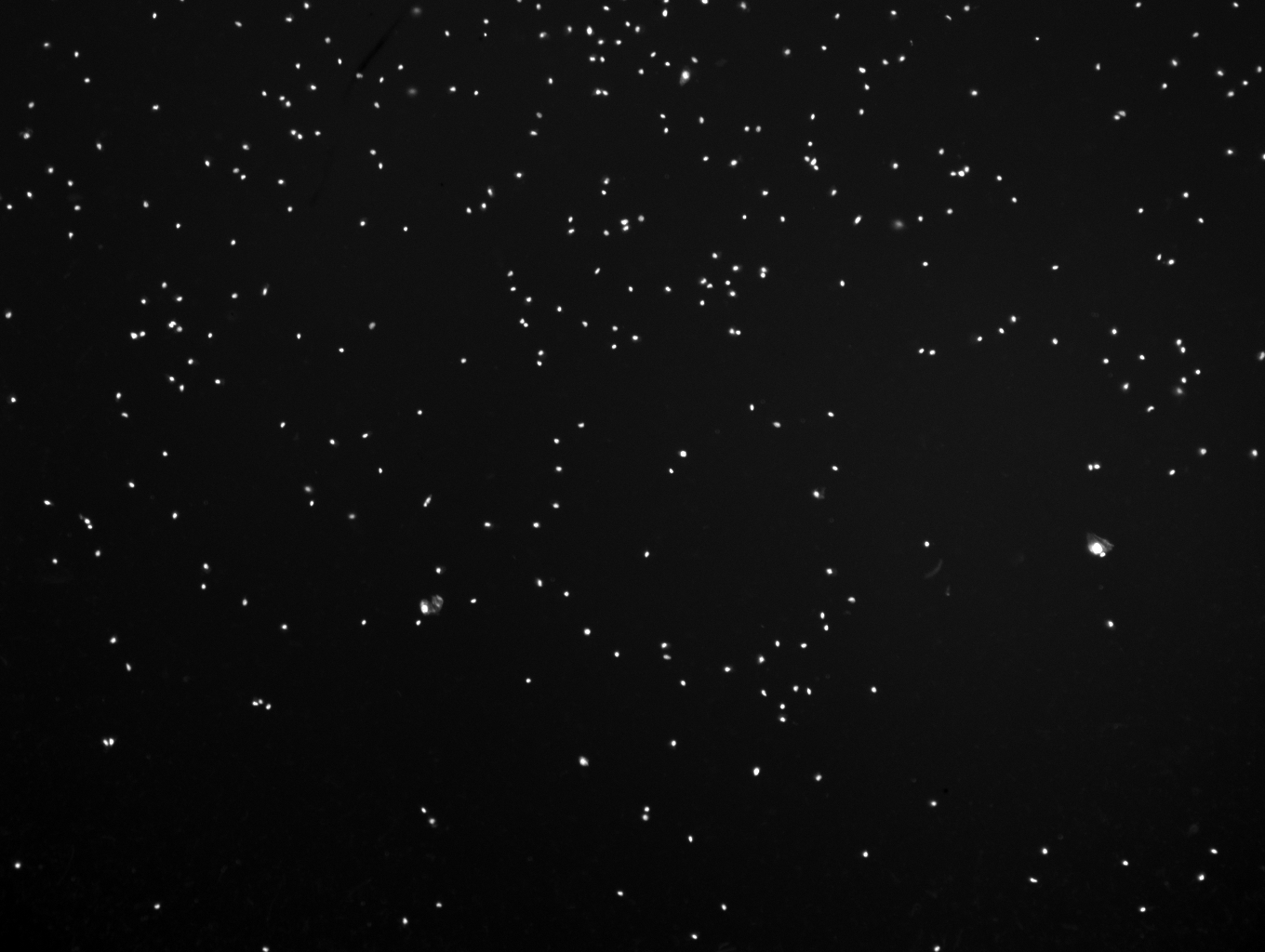

Supplement: Figure 2—figure supplement 2—source data 1. [file elife-38187-fig2-figsupp2-data1.zip › Figure 2 - figure suppl 2 - source data/Exp4/Plate A/r04c08f16p01-ch1sk1fk1fl1.tiff]

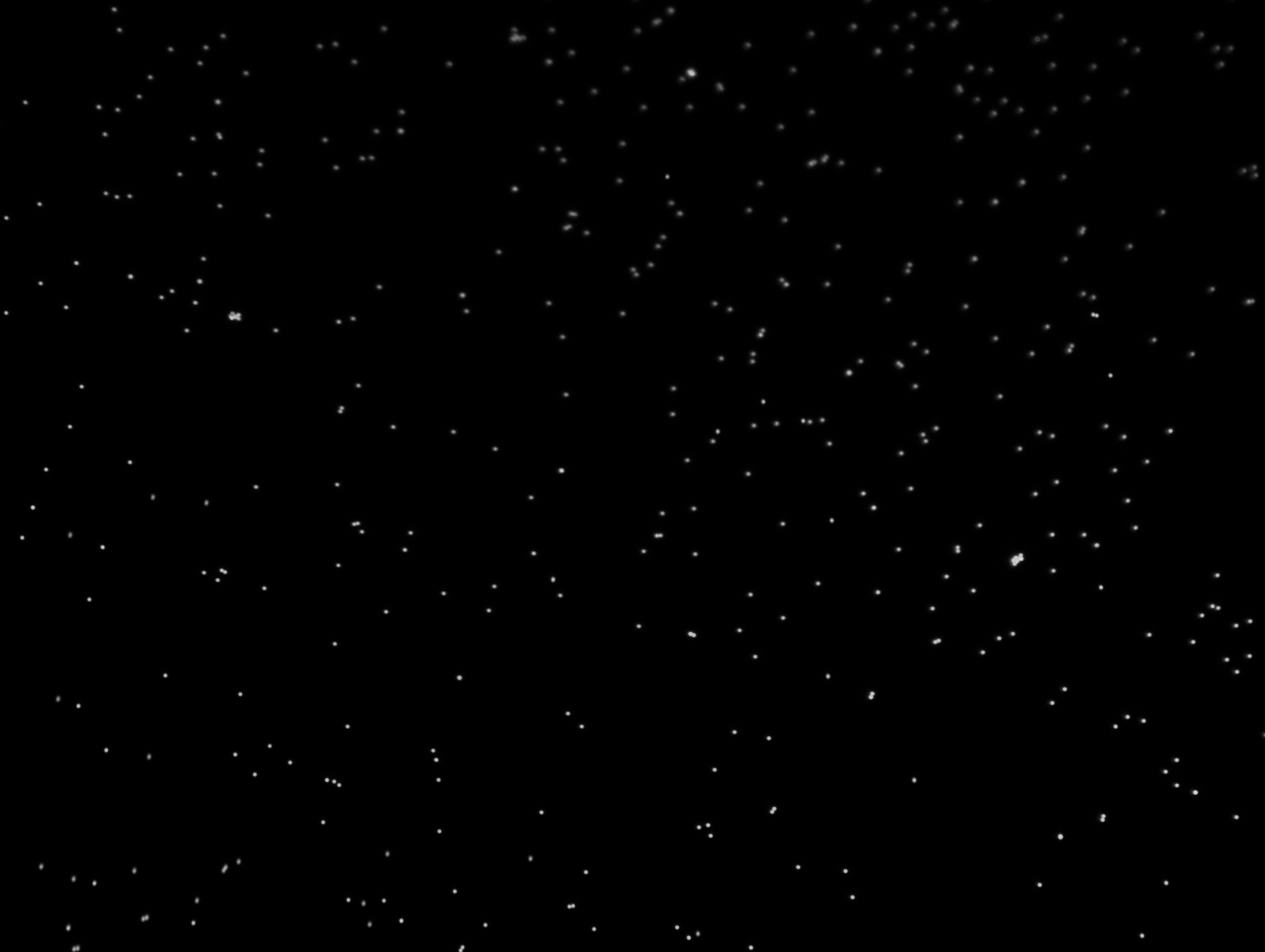

Supplement: Figure 2—figure supplement 2—source data 1. [file elife-38187-fig2-figsupp2-data1.zip › Figure 2 - figure suppl 2 - source data/Exp4/Plate A/r04c08f16p01-ch2sk1fk1fl1.tiff]

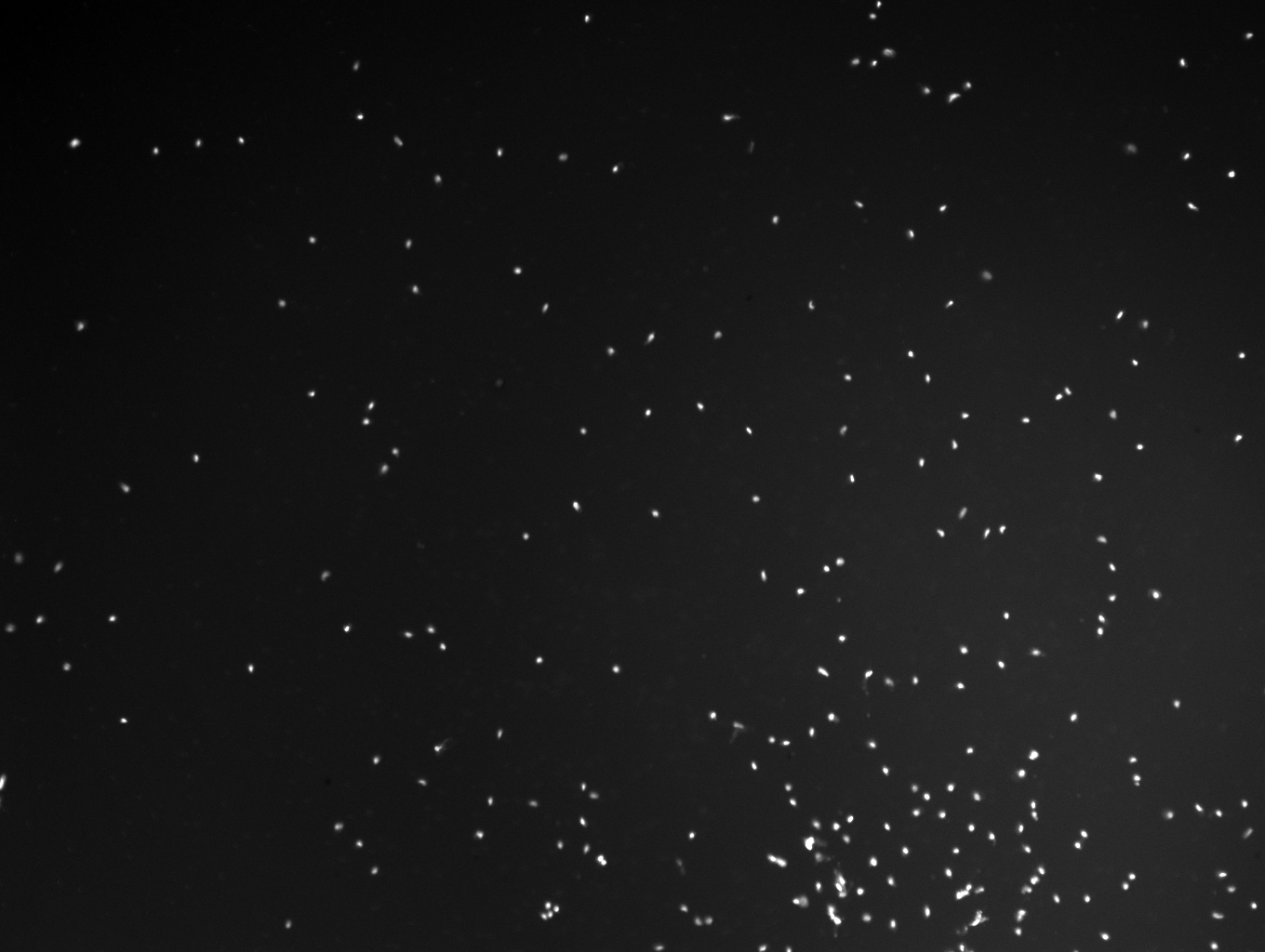

Supplement: Figure 2—figure supplement 2—source data 1. [file elife-38187-fig2-figsupp2-data1.zip › Figure 2 - figure suppl 2 - source data/Exp4/Plate A/r05c05f01p01-ch1sk1fk1fl1.tiff]

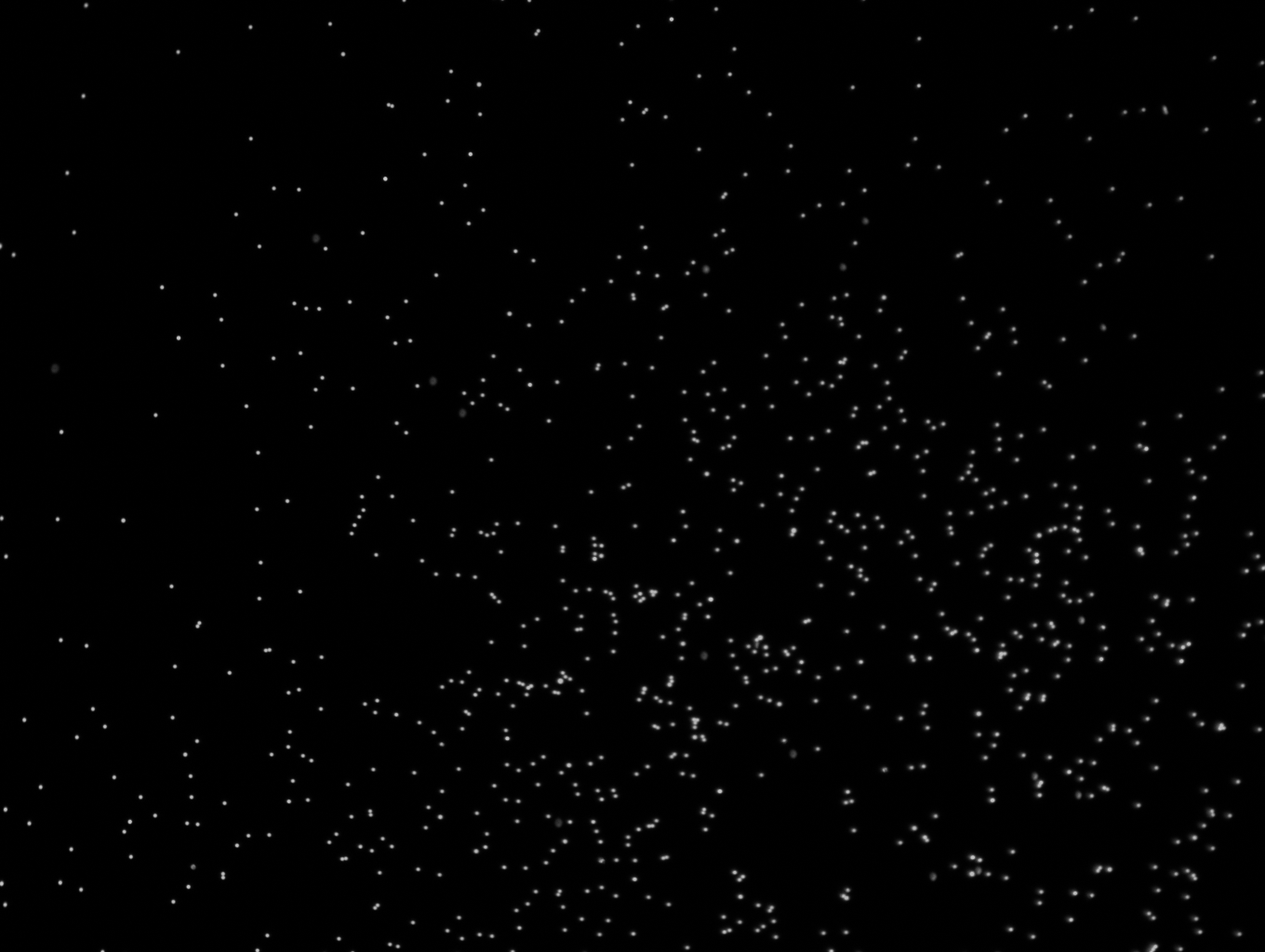

Supplement: Figure 2—figure supplement 2—source data 1. [file elife-38187-fig2-figsupp2-data1.zip › Figure 2 - figure suppl 2 - source data/Exp4/Plate A/r05c05f01p01-ch2sk1fk1fl1.tiff]

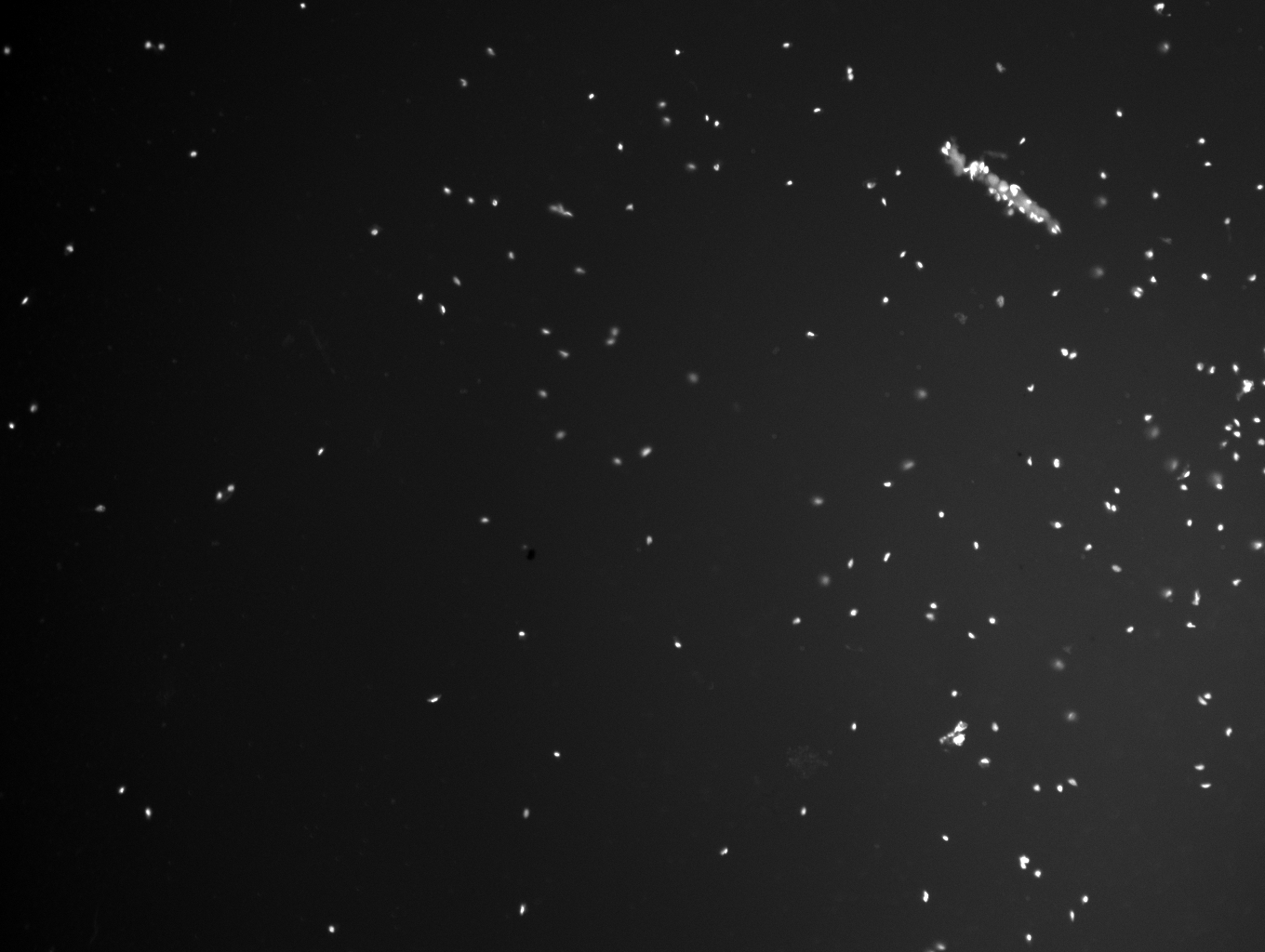

Supplement: Figure 2—figure supplement 2—source data 1. [file elife-38187-fig2-figsupp2-data1.zip › Figure 2 - figure suppl 2 - source data/Exp4/Plate A/r06c04f01p01-ch1sk1fk1fl1.tiff]

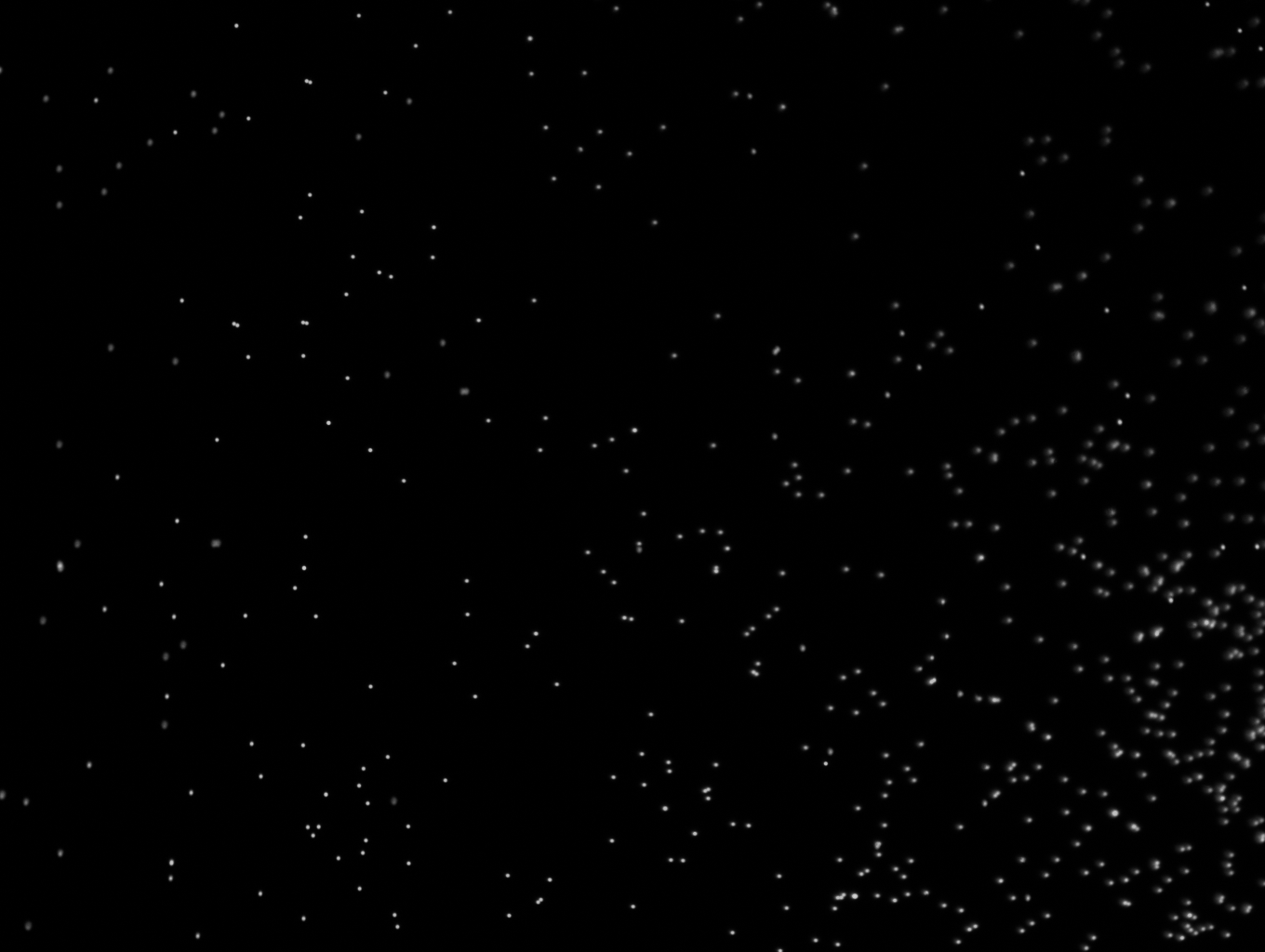

Supplement: Figure 2—figure supplement 2—source data 1. [file elife-38187-fig2-figsupp2-data1.zip › Figure 2 - figure suppl 2 - source data/Exp4/Plate A/r06c04f01p01-ch2sk1fk1fl1.tiff]

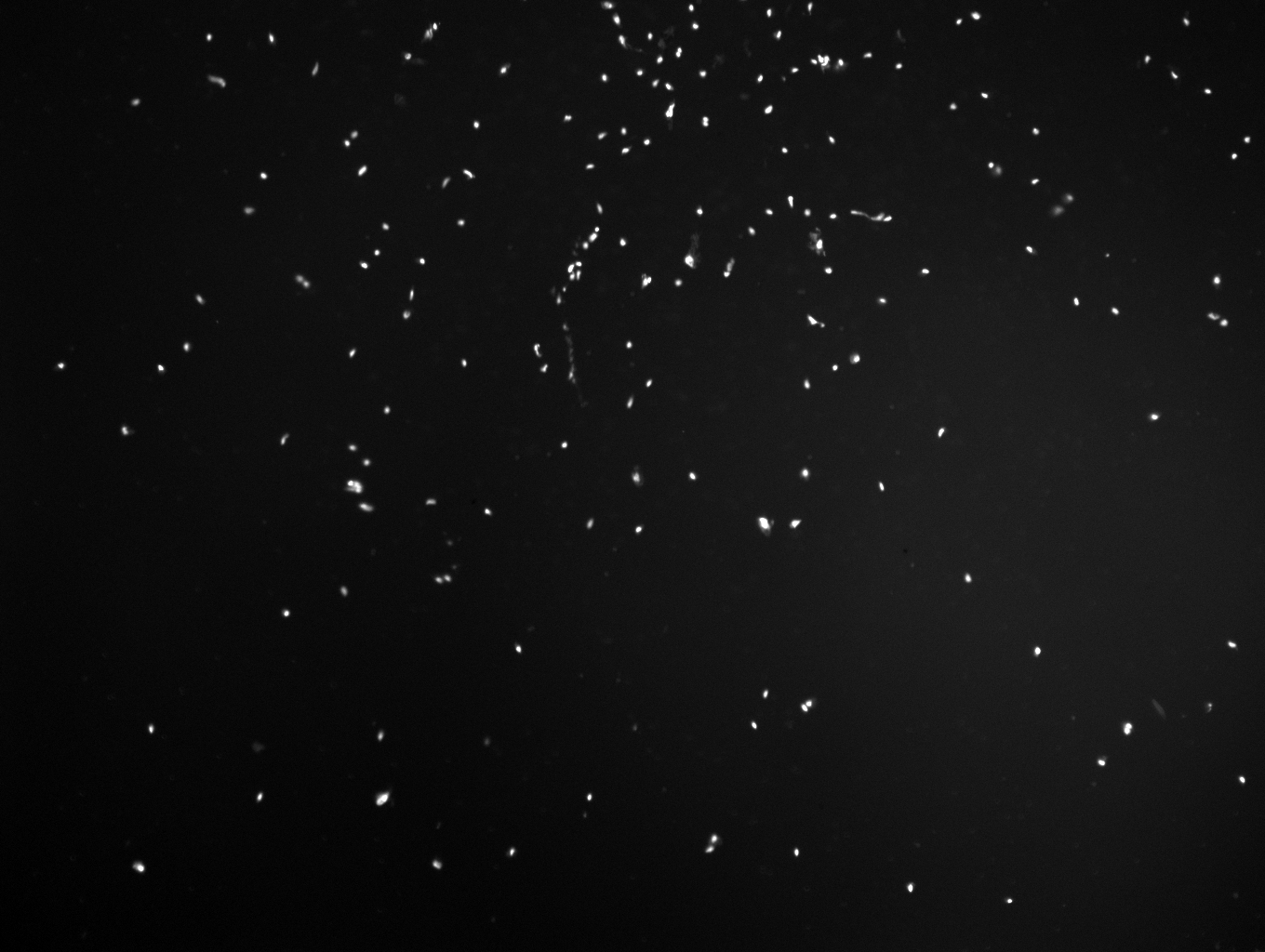

Supplement: Figure 2—figure supplement 2—source data 1. [file elife-38187-fig2-figsupp2-data1.zip › Figure 2 - figure suppl 2 - source data/Exp4/Plate A/r06c06f01p01-ch1sk1fk1fl1.tiff]

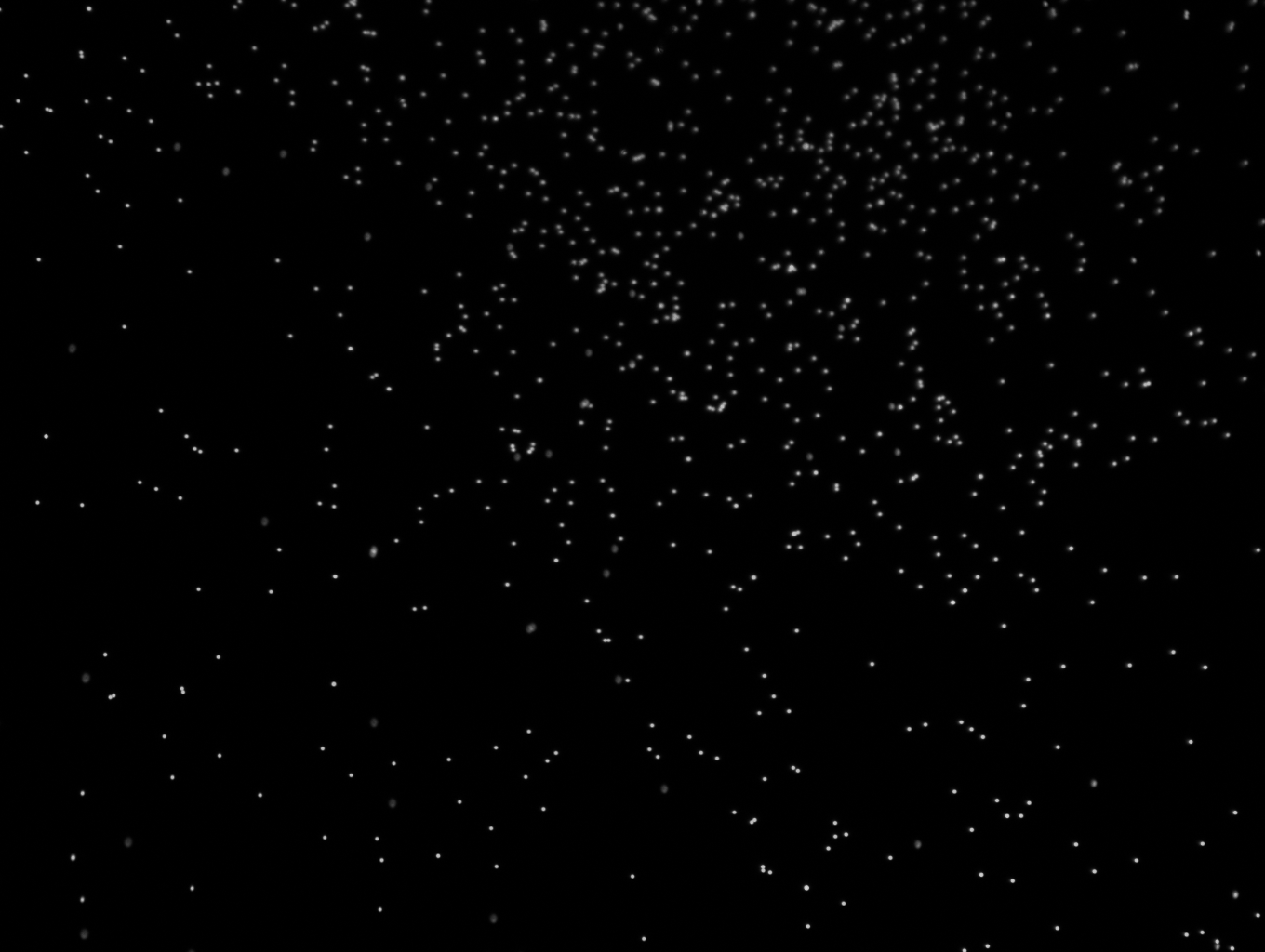

Supplement: Figure 2—figure supplement 2—source data 1. [file elife-38187-fig2-figsupp2-data1.zip › Figure 2 - figure suppl 2 - source data/Exp4/Plate A/r06c06f01p01-ch2sk1fk1fl1.tiff]

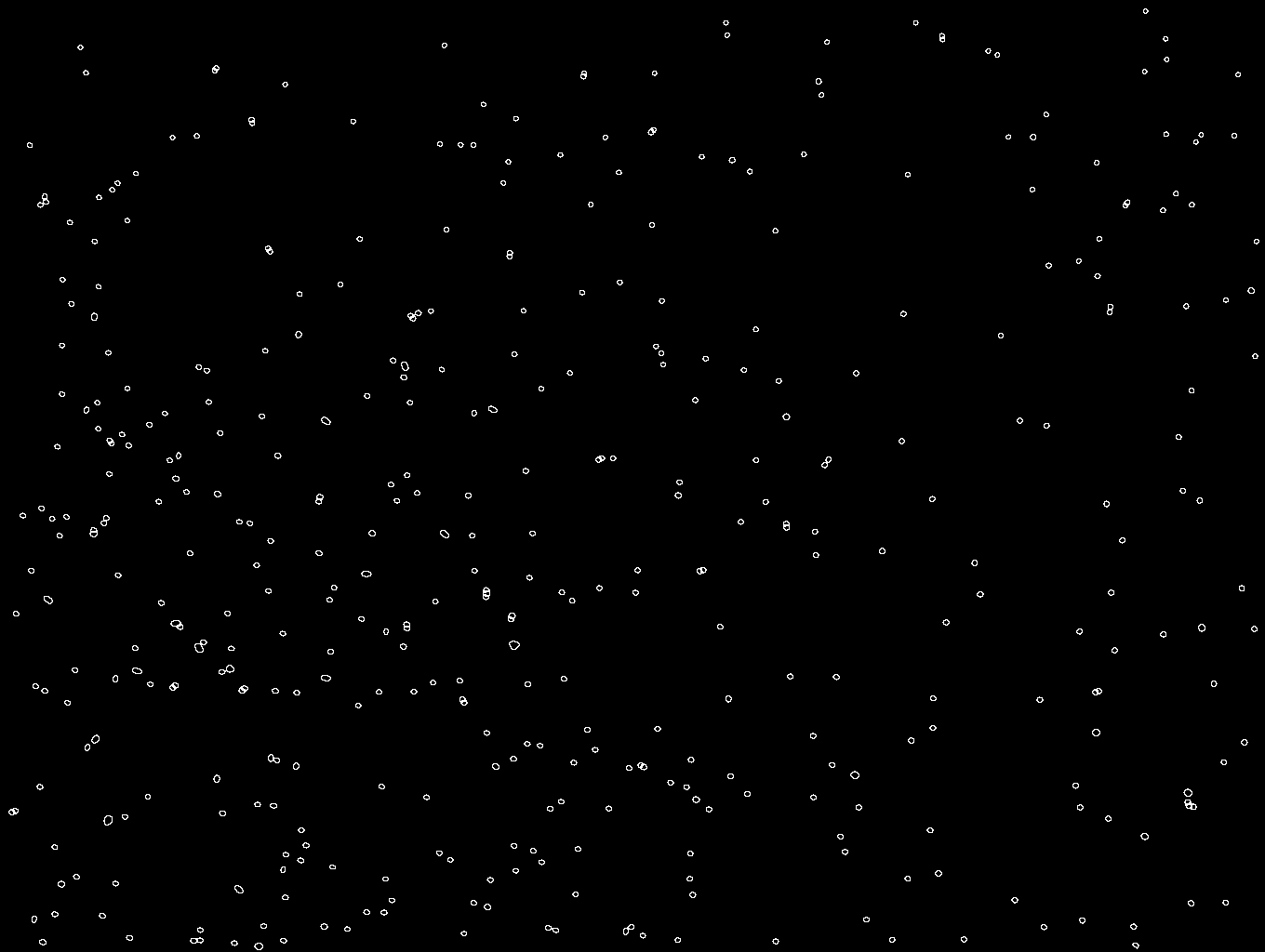

Supplement: Figure 2—figure supplement 2—source data 1. [file elife-38187-fig2-figsupp2-data1.zip › Figure 2 - figure suppl 2 - source data/Exp4/Plate A/Segmentation/r02c02f01p01-beads_outline.tif.tif]

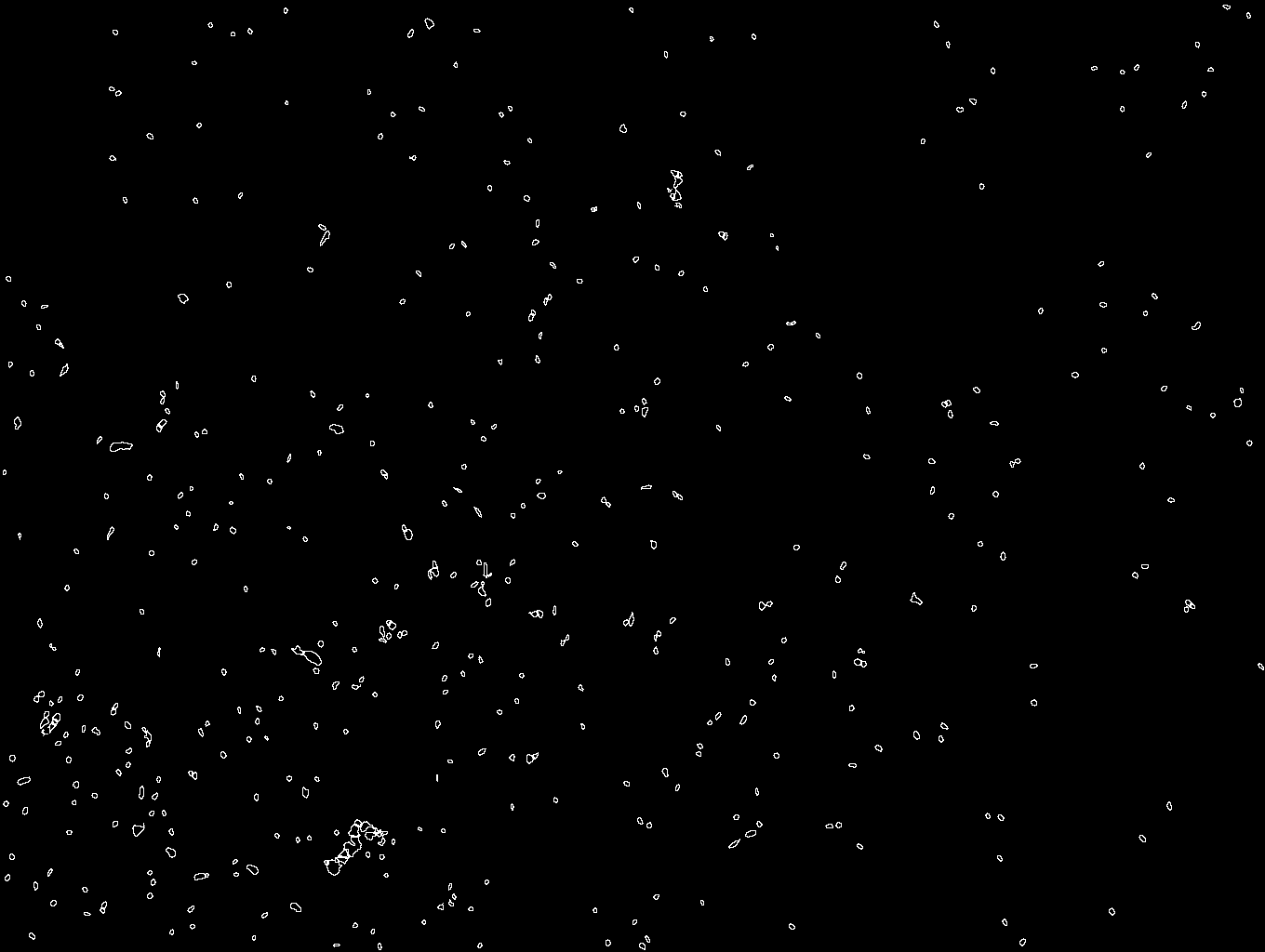

Supplement: Figure 2—figure supplement 2—source data 1. [file elife-38187-fig2-figsupp2-data1.zip › Figure 2 - figure suppl 2 - source data/Exp4/Plate A/Segmentation/r02c02f01p01-cell_outline.tif.tif]

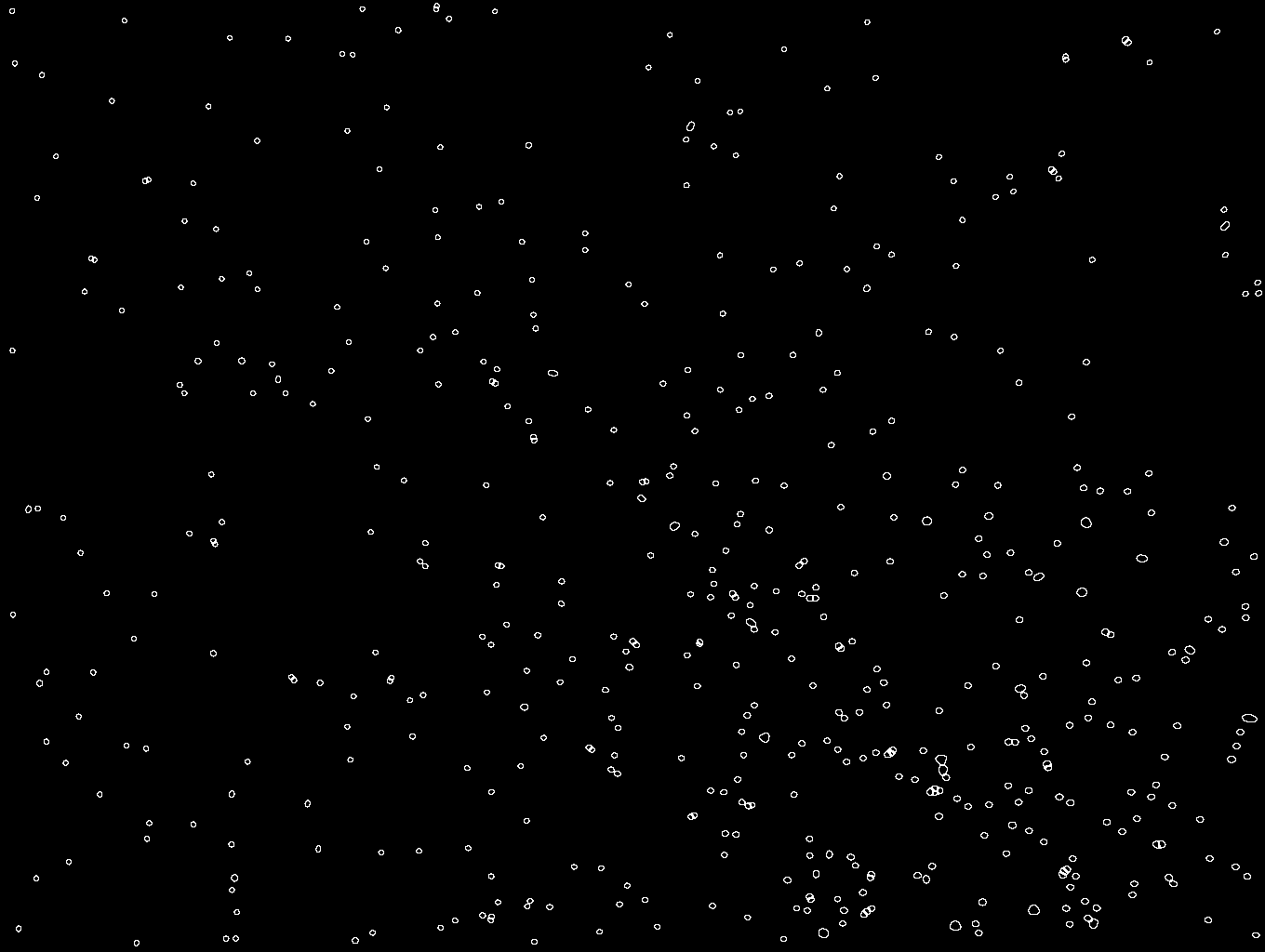

Supplement: Figure 2—figure supplement 2—source data 1. [file elife-38187-fig2-figsupp2-data1.zip › Figure 2 - figure suppl 2 - source data/Exp4/Plate A/Segmentation/r02c05f01p01-beads_outline.tif.tif]

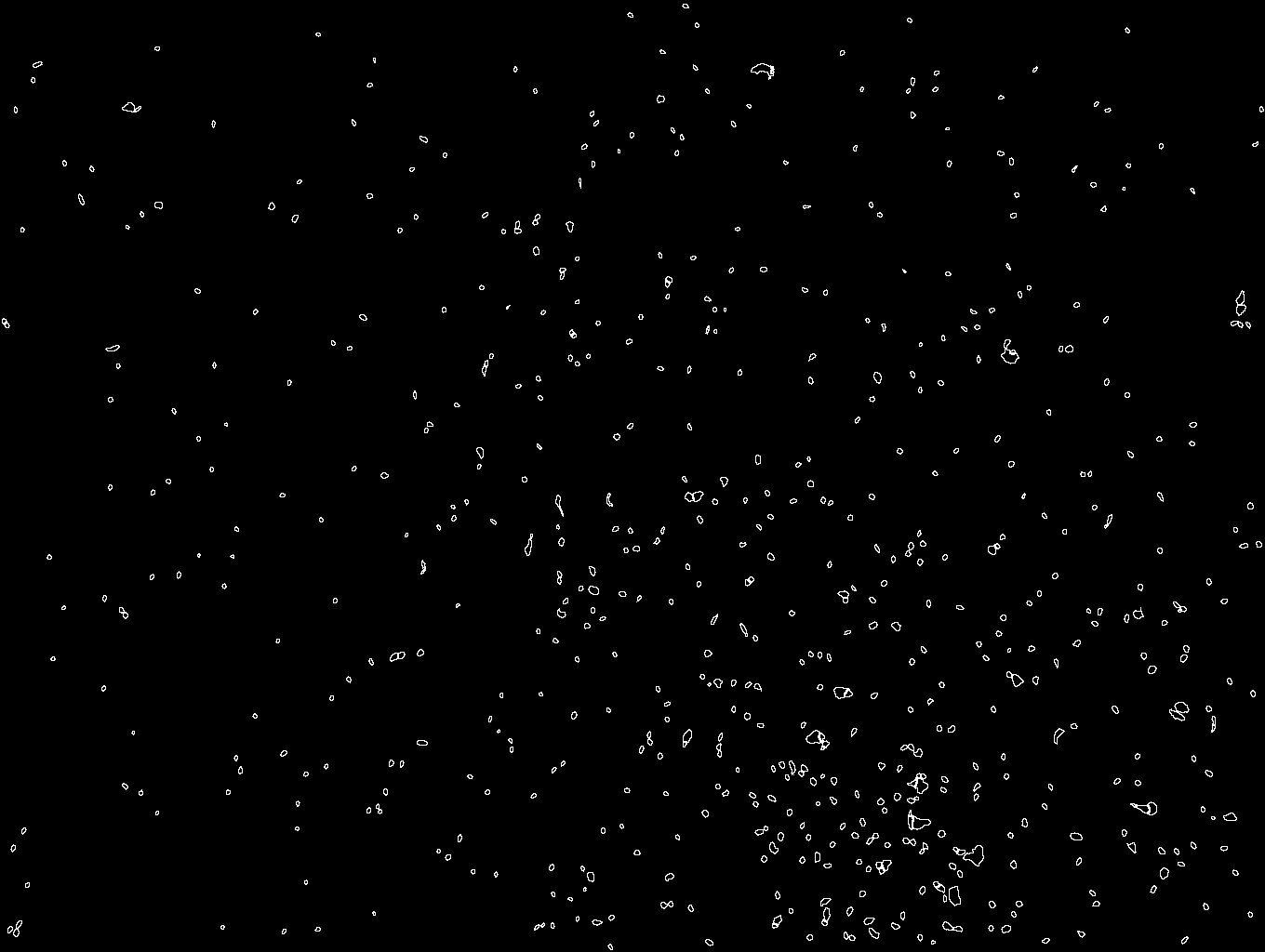

Supplement: Figure 2—figure supplement 2—source data 1. [file elife-38187-fig2-figsupp2-data1.zip › Figure 2 - figure suppl 2 - source data/Exp4/Plate A/Segmentation/r02c05f01p01-cell_outline.tif.tif]

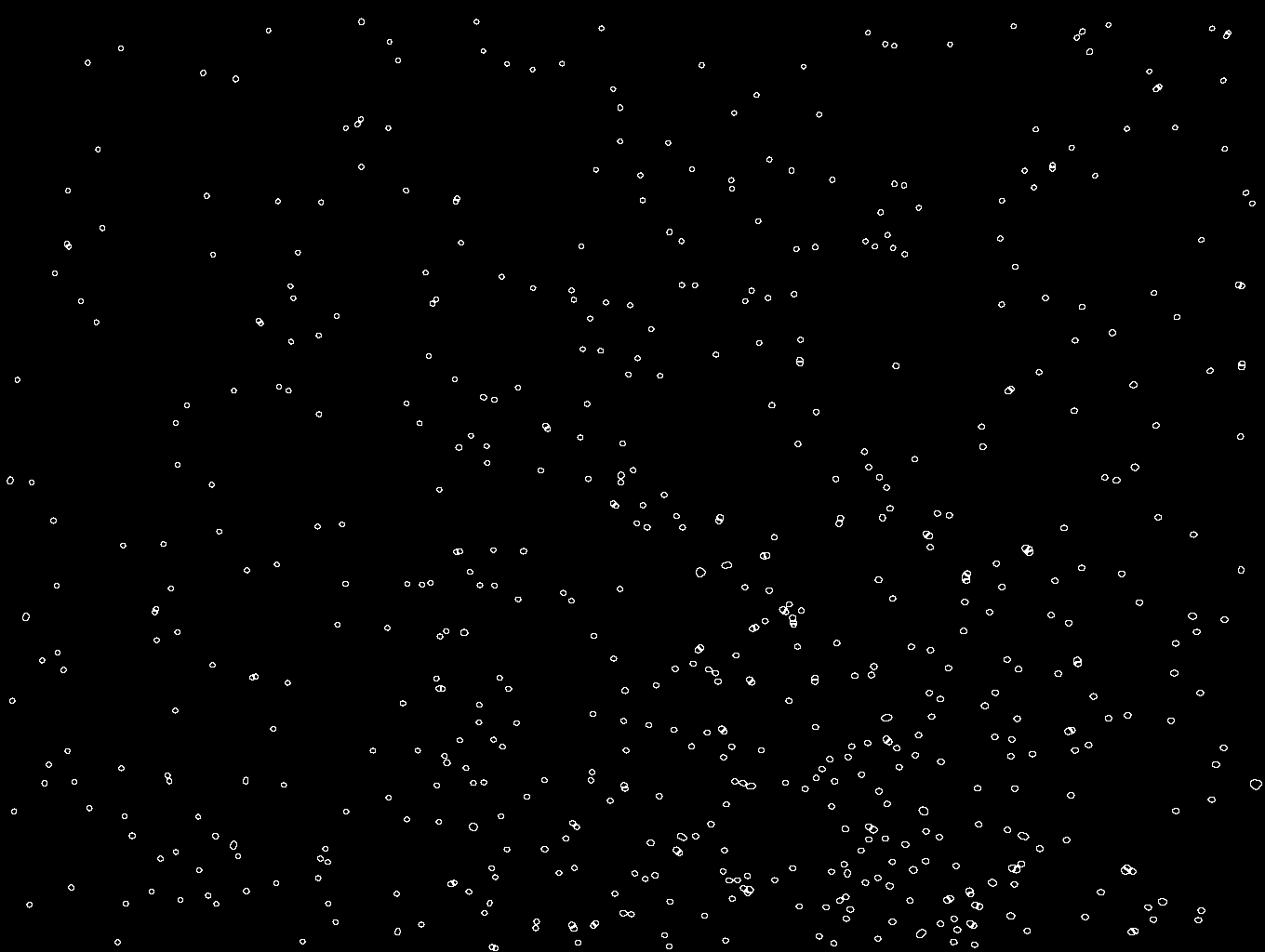

Supplement: Figure 2—figure supplement 2—source data 1. [file elife-38187-fig2-figsupp2-data1.zip › Figure 2 - figure suppl 2 - source data/Exp4/Plate A/Segmentation/r02c09f01p01-beads_outline.tif.tif]

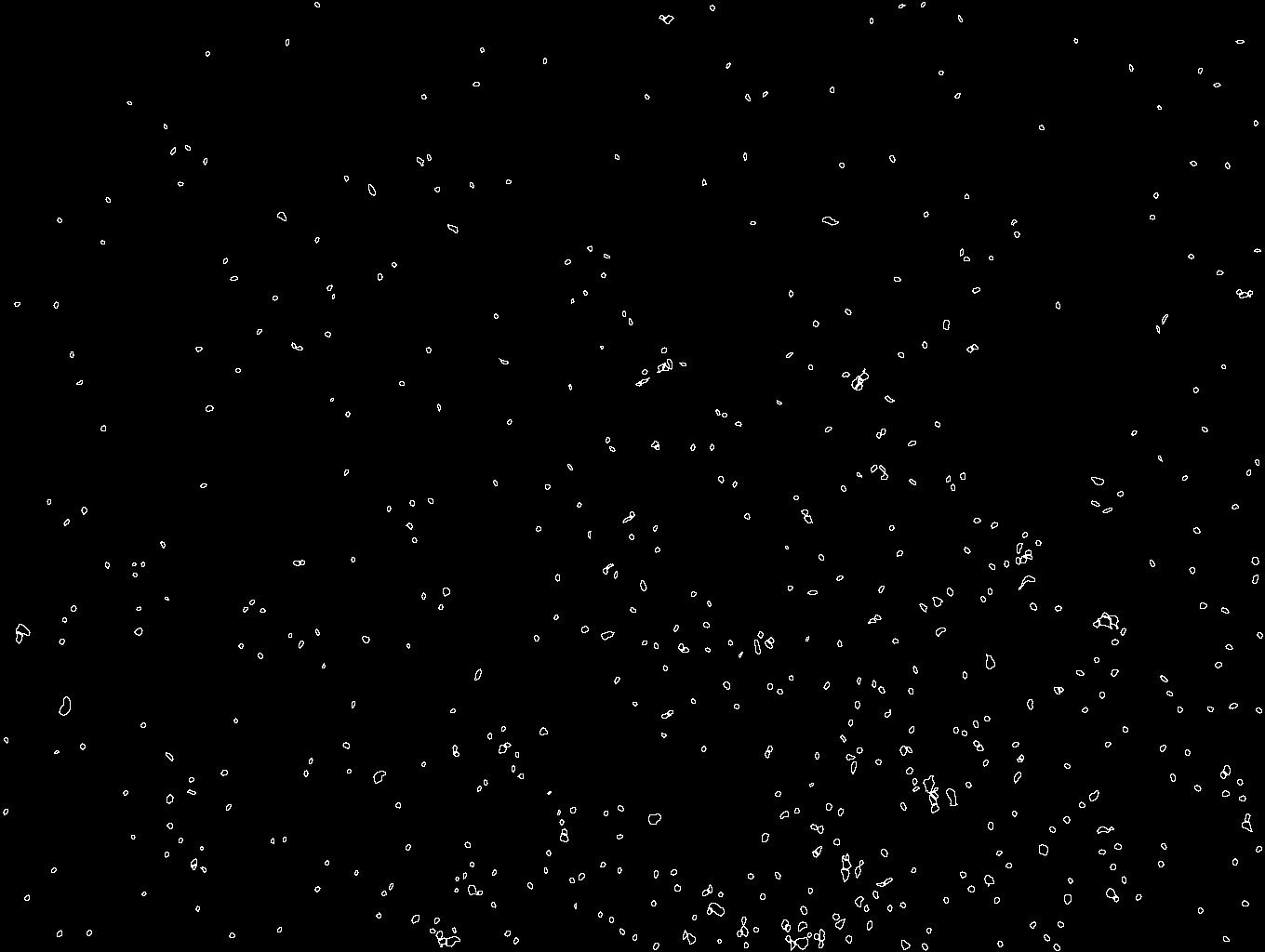

Supplement: Figure 2—figure supplement 2—source data 1. [file elife-38187-fig2-figsupp2-data1.zip › Figure 2 - figure suppl 2 - source data/Exp4/Plate A/Segmentation/r02c09f01p01-cell_outline.tif.tif]

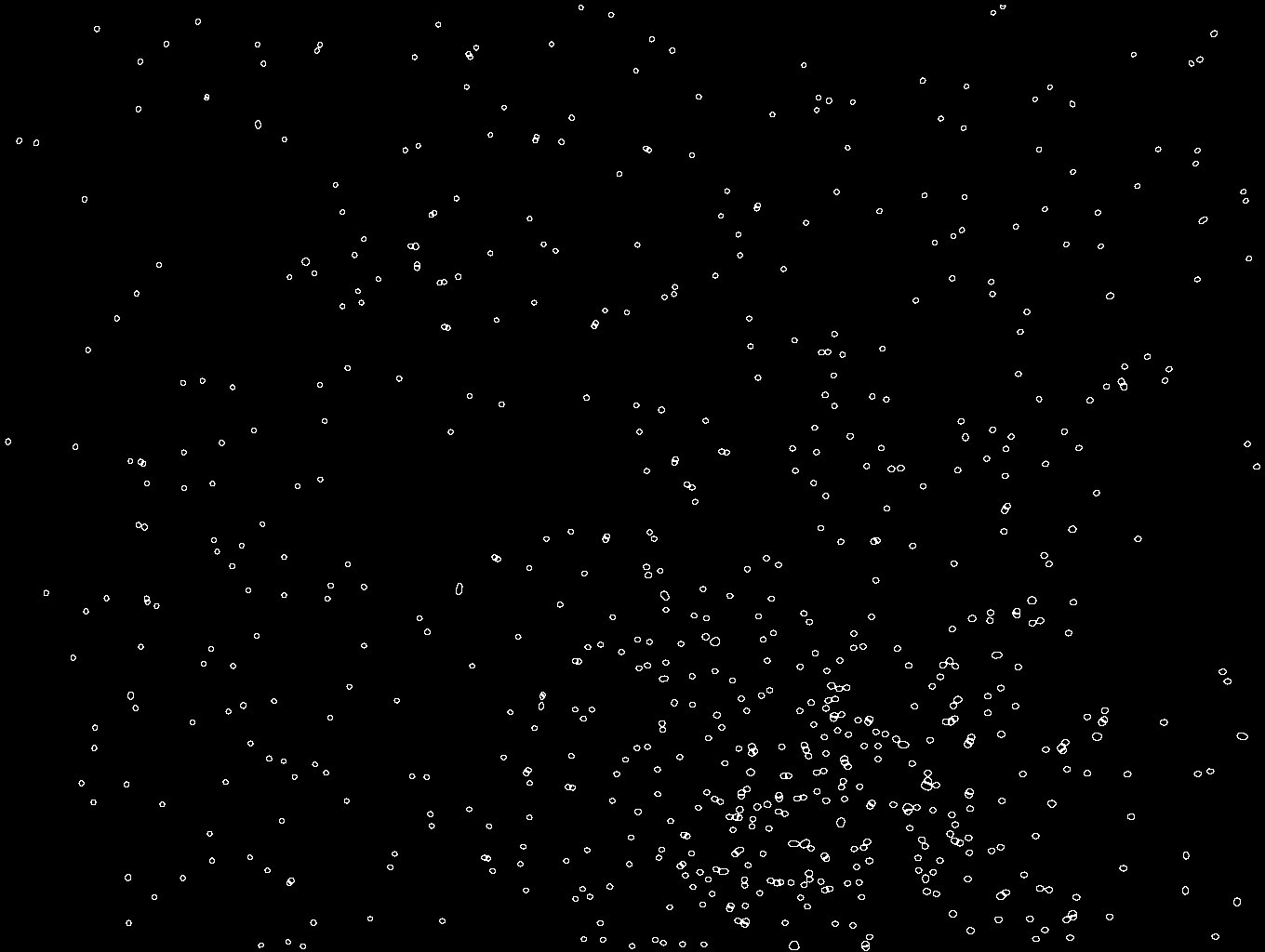

Supplement: Figure 2—figure supplement 2—source data 1. [file elife-38187-fig2-figsupp2-data1.zip › Figure 2 - figure suppl 2 - source data/Exp4/Plate A/Segmentation/r03c04f01p01-beads_outline.tif.tif]

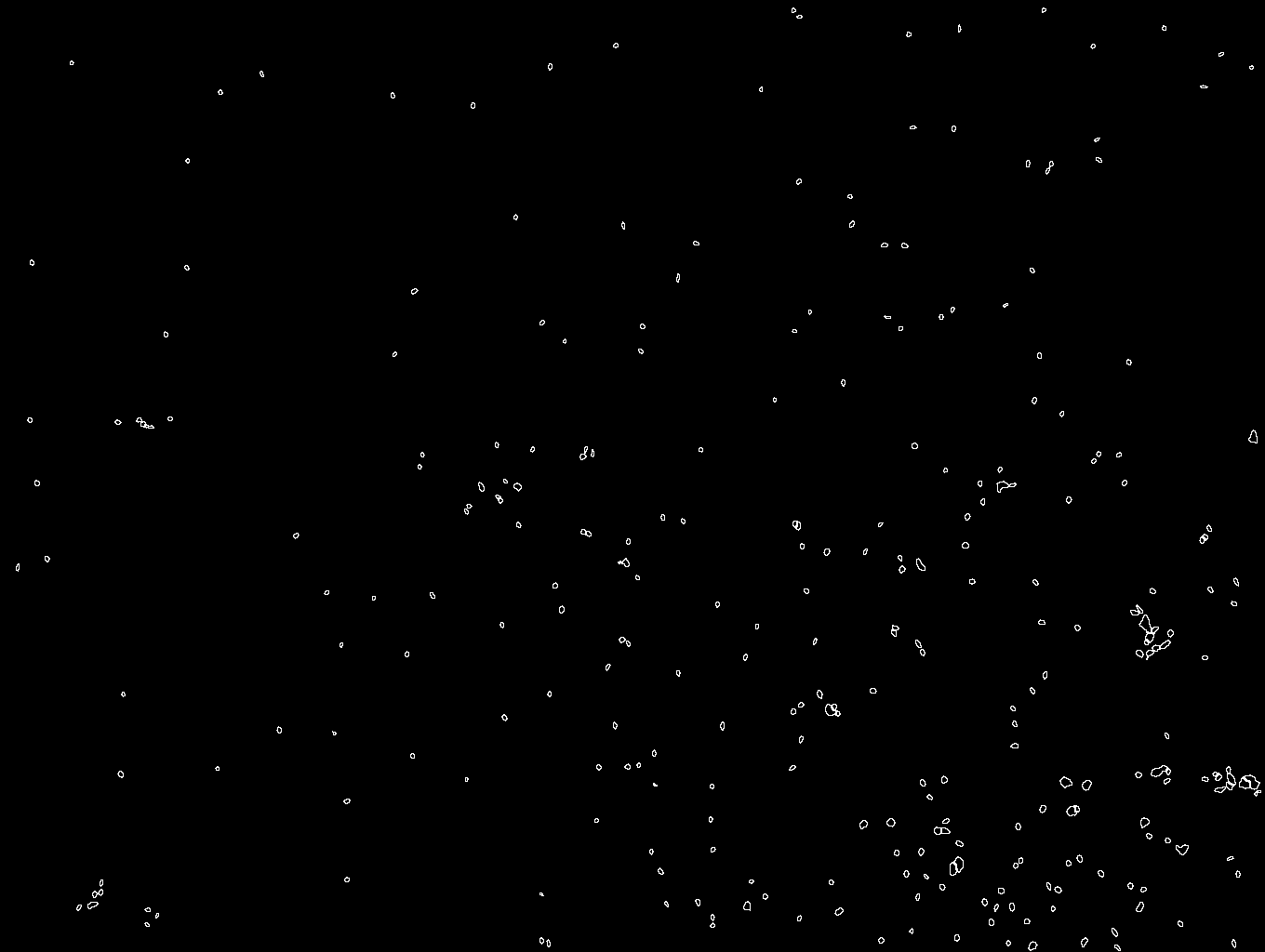

Supplement: Figure 2—figure supplement 2—source data 1. [file elife-38187-fig2-figsupp2-data1.zip › Figure 2 - figure suppl 2 - source data/Exp4/Plate A/Segmentation/r03c04f01p01-cell_outline.tif.tif]

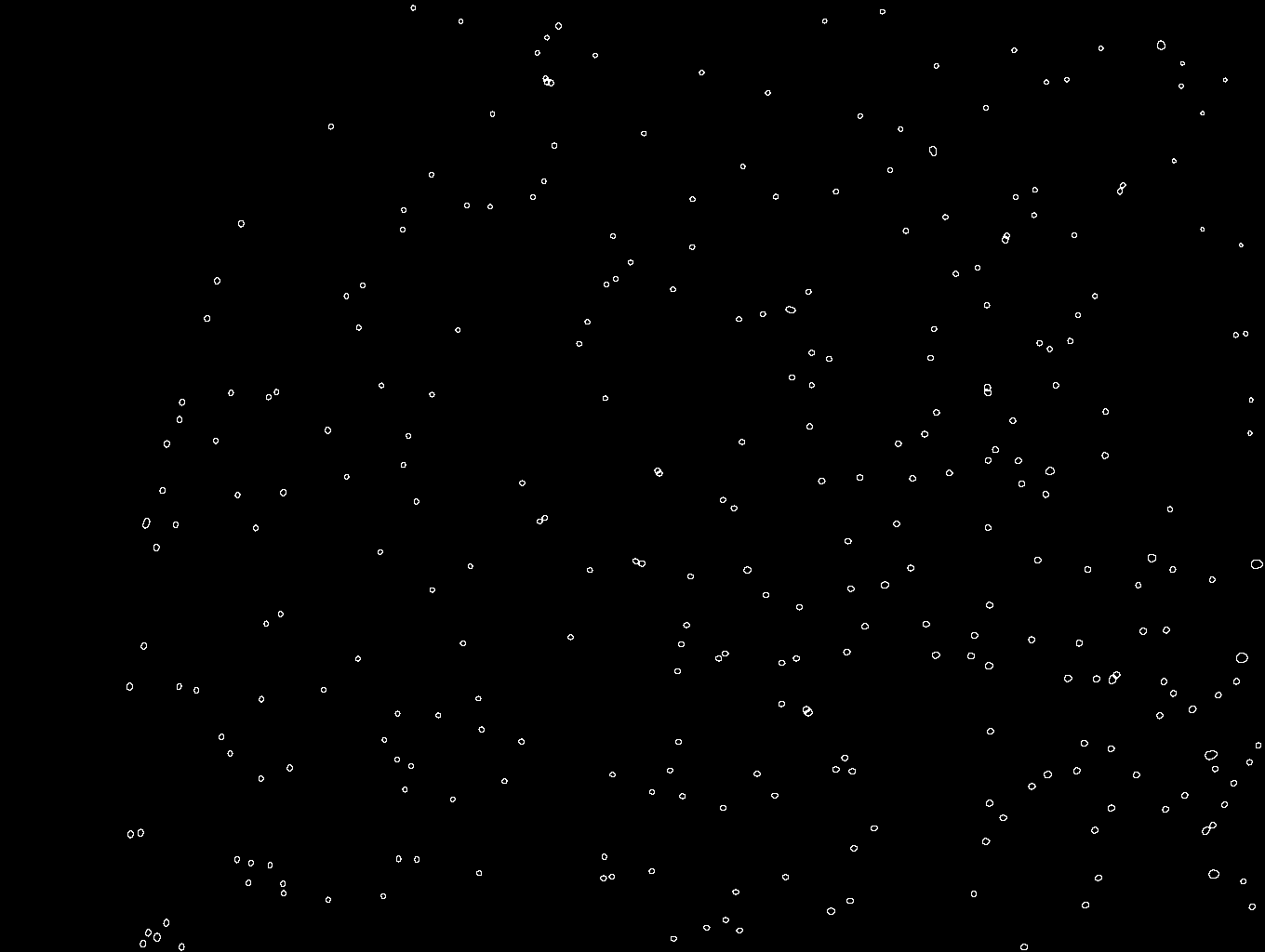

Supplement: Figure 2—figure supplement 2—source data 1. [file elife-38187-fig2-figsupp2-data1.zip › Figure 2 - figure suppl 2 - source data/Exp4/Plate A/Segmentation/r03c09f01p01-beads_outline.tif.tif]

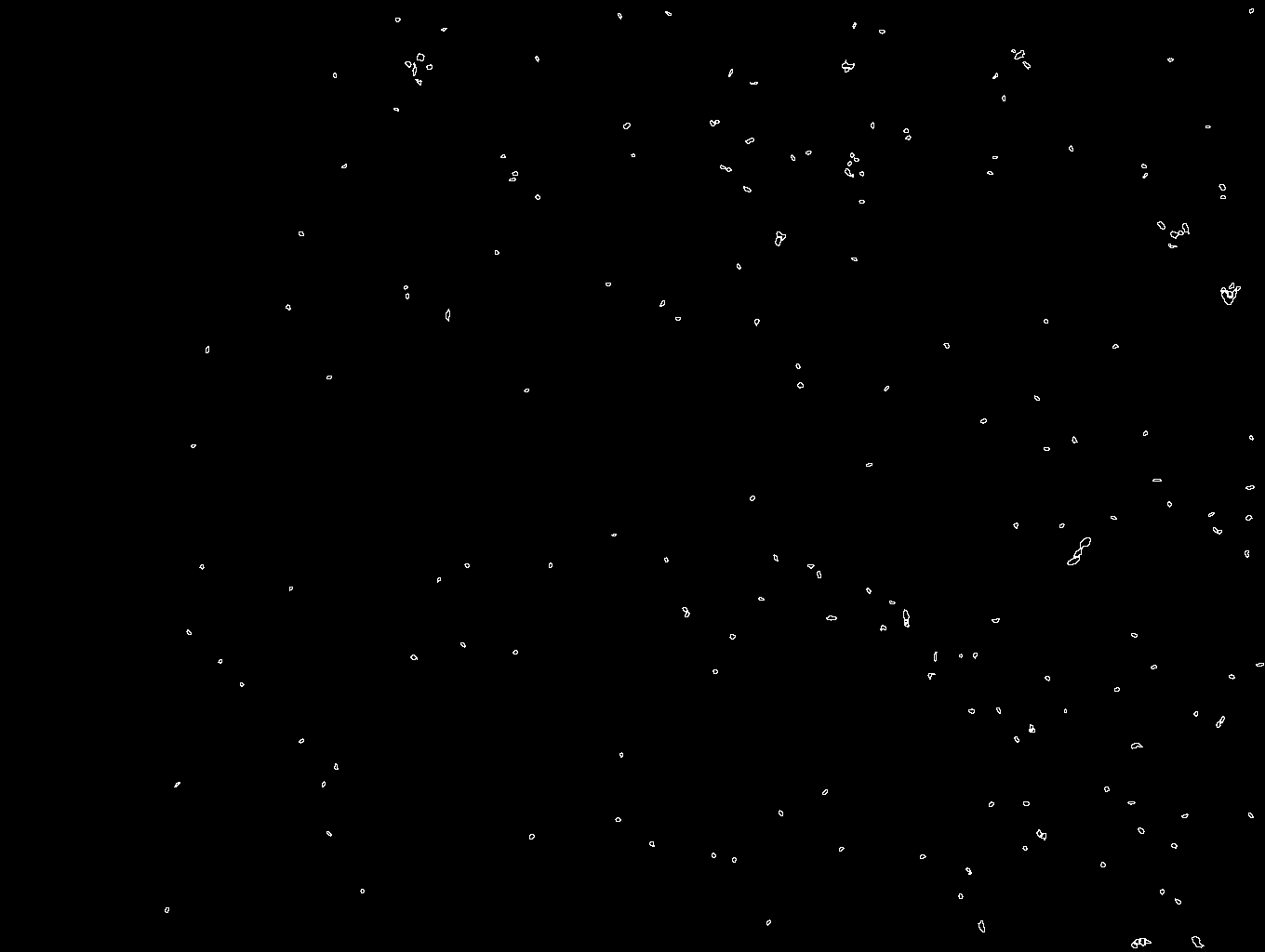

Supplement: Figure 2—figure supplement 2—source data 1. [file elife-38187-fig2-figsupp2-data1.zip › Figure 2 - figure suppl 2 - source data/Exp4/Plate A/Segmentation/r03c09f01p01-cell_outline.tif.tif]

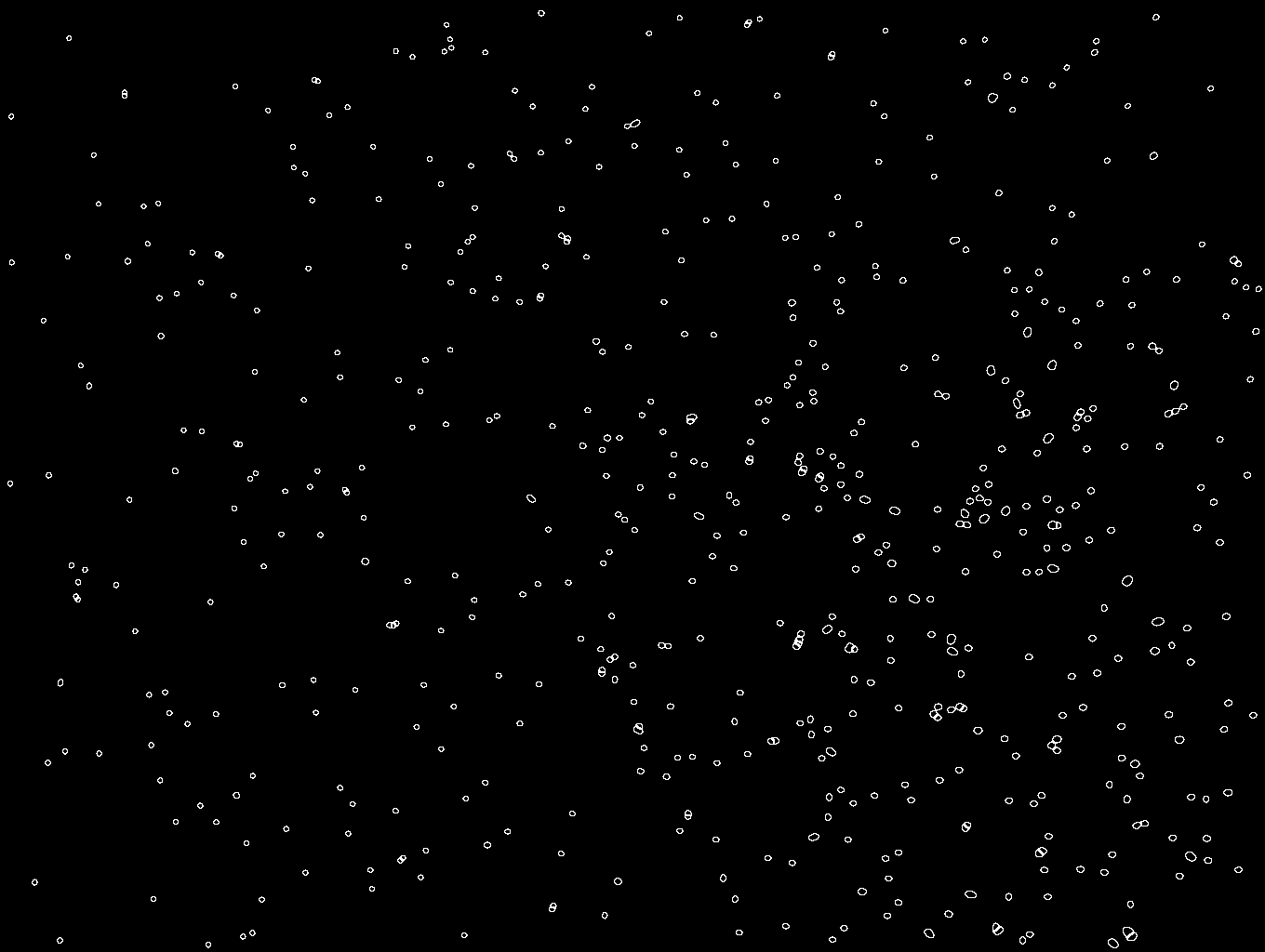

Supplement: Figure 2—figure supplement 2—source data 1. [file elife-38187-fig2-figsupp2-data1.zip › Figure 2 - figure suppl 2 - source data/Exp4/Plate A/Segmentation/r04c02f01p01-beads_outline.tif.tif]

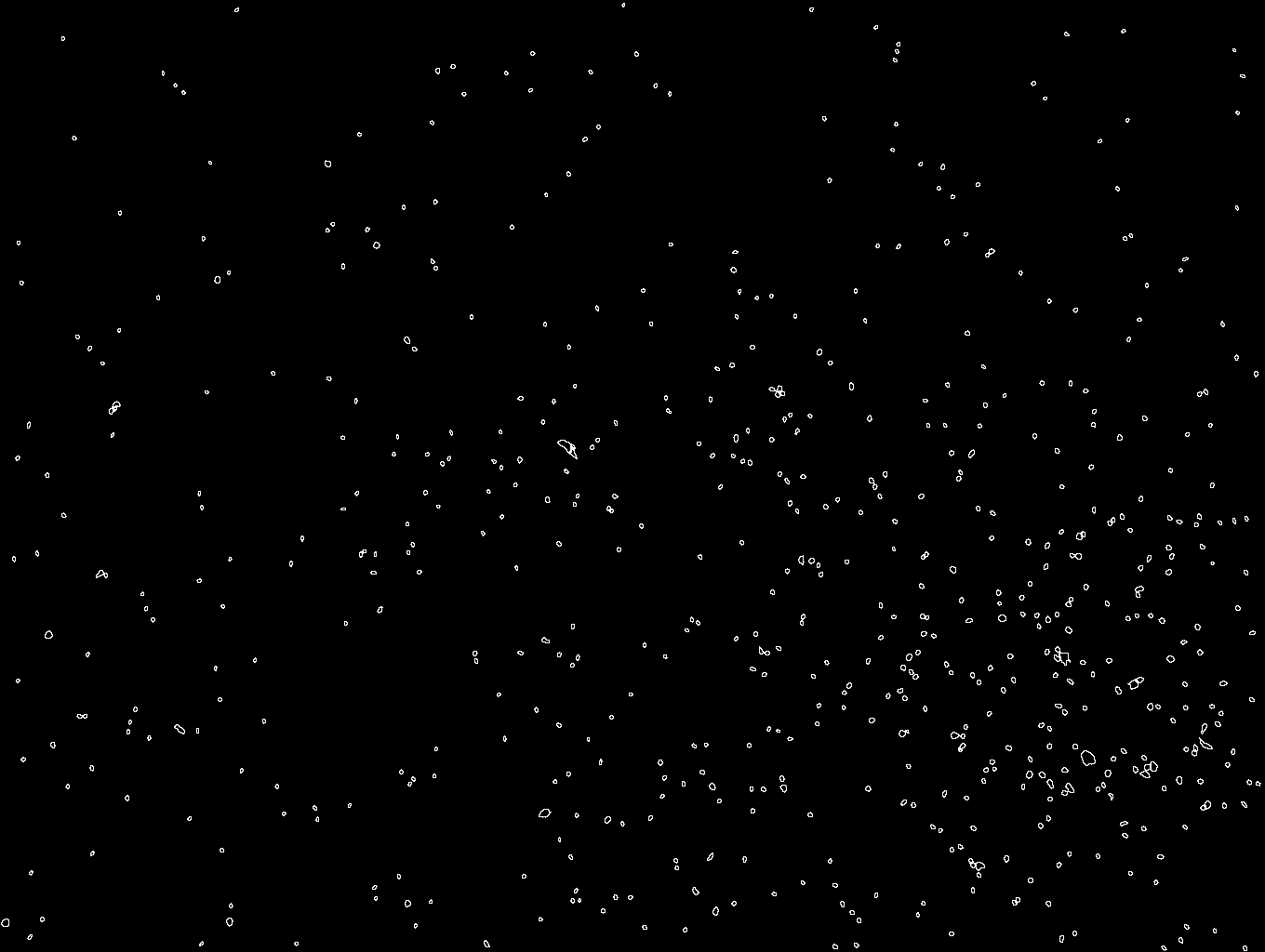

Supplement: Figure 2—figure supplement 2—source data 1. [file elife-38187-fig2-figsupp2-data1.zip › Figure 2 - figure suppl 2 - source data/Exp4/Plate A/Segmentation/r04c02f01p01-cell_outline.tif.tif]

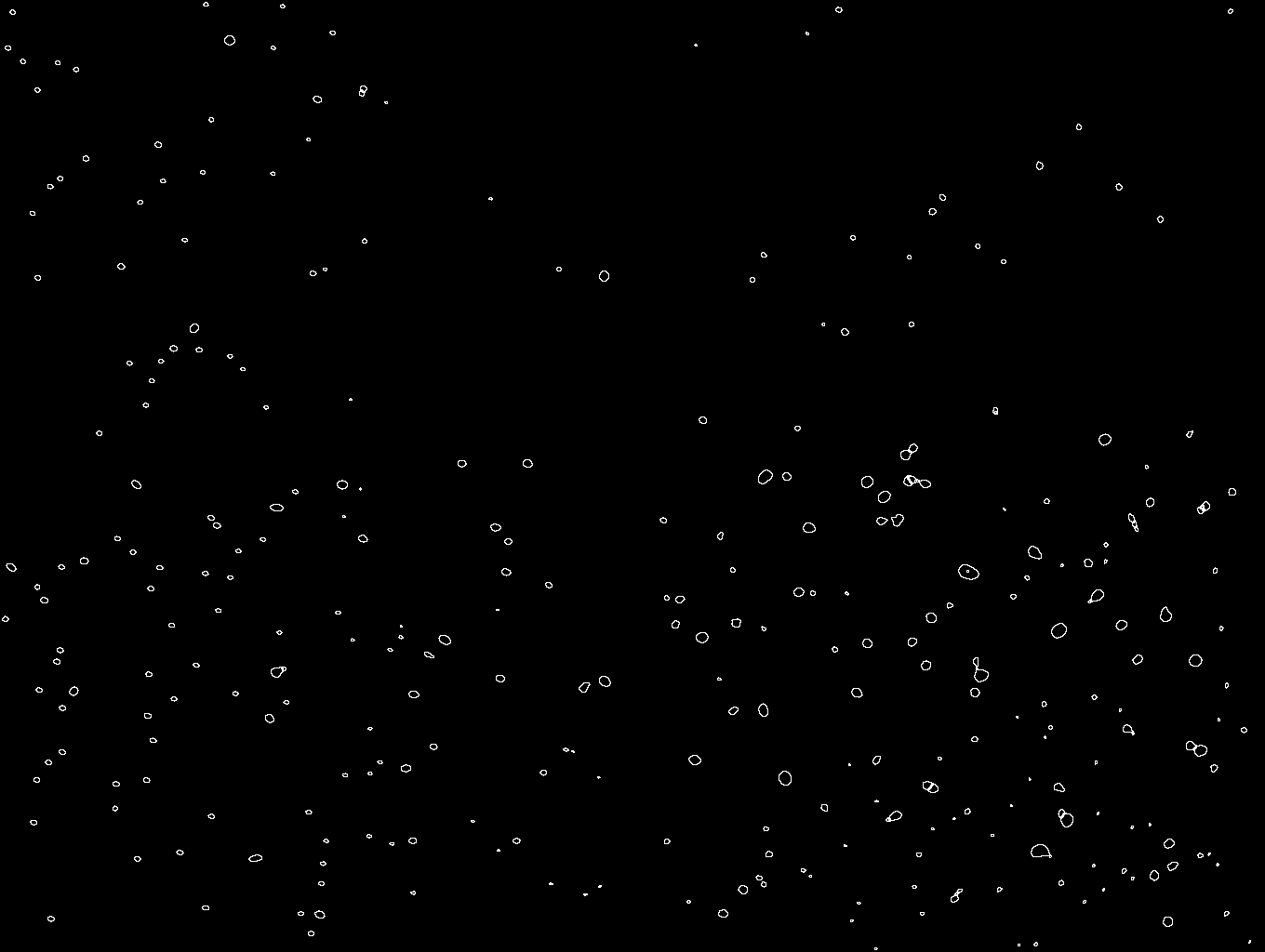

Supplement: Figure 2—figure supplement 2—source data 1. [file elife-38187-fig2-figsupp2-data1.zip › Figure 2 - figure suppl 2 - source data/Exp4/Plate A/Segmentation/r04c03f01p01-beads_outline.tif.tif]

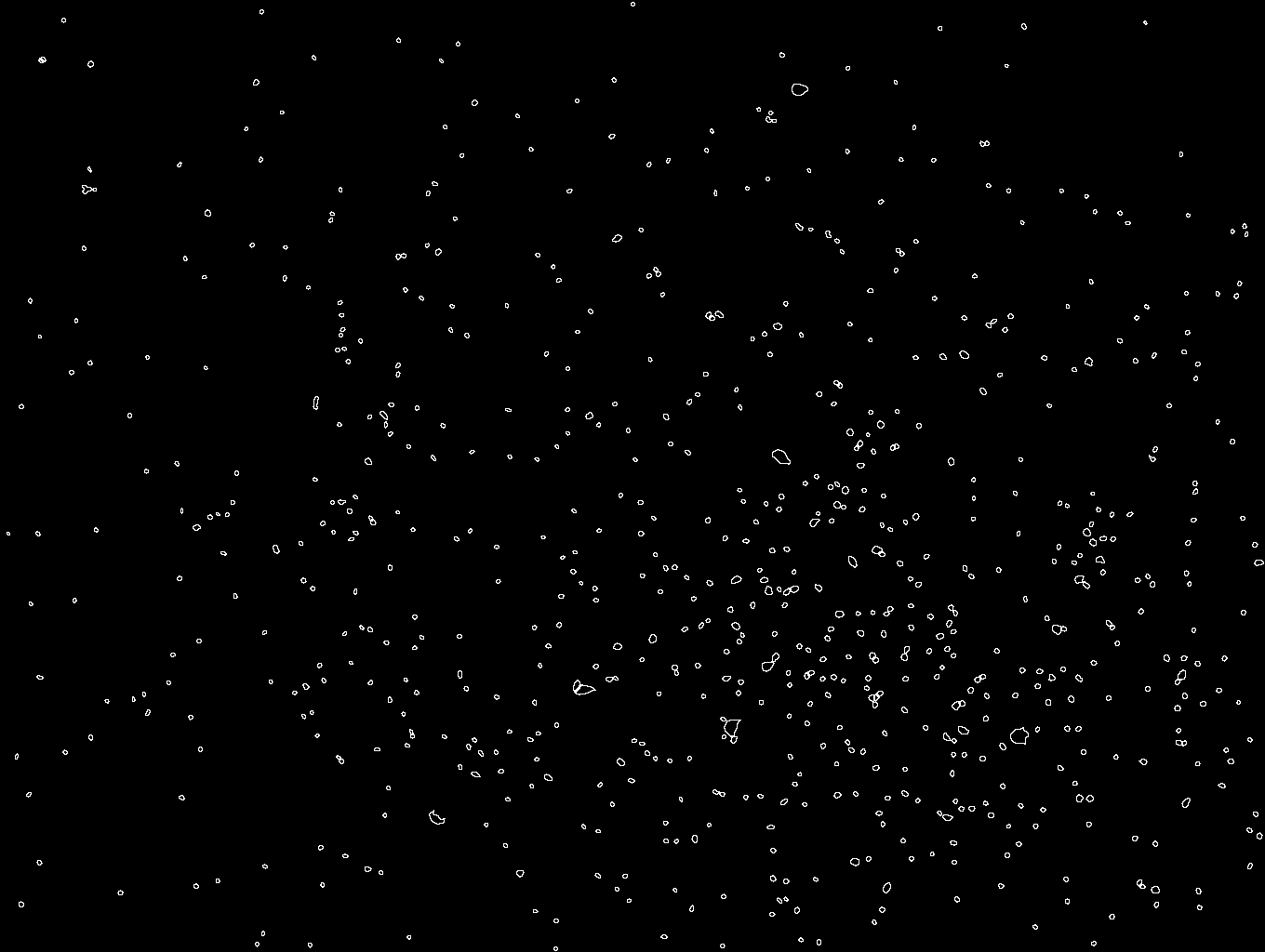

Supplement: Figure 2—figure supplement 2—source data 1. [file elife-38187-fig2-figsupp2-data1.zip › Figure 2 - figure suppl 2 - source data/Exp4/Plate A/Segmentation/r04c03f01p01-cell_outline.tif.tif]

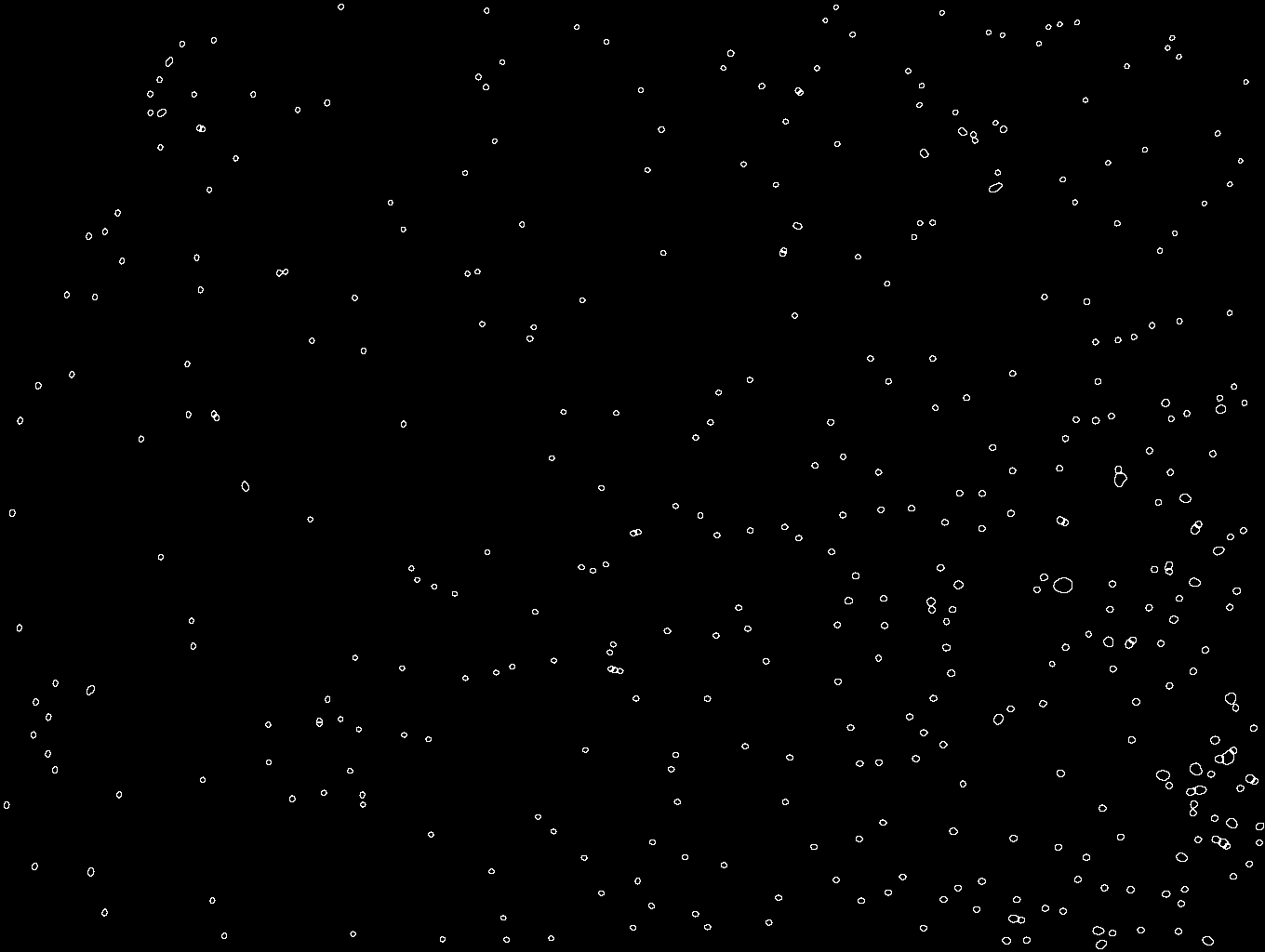

Supplement: Figure 2—figure supplement 2—source data 1. [file elife-38187-fig2-figsupp2-data1.zip › Figure 2 - figure suppl 2 - source data/Exp4/Plate A/Segmentation/r04c07f01p01-beads_outline.tif.tif]

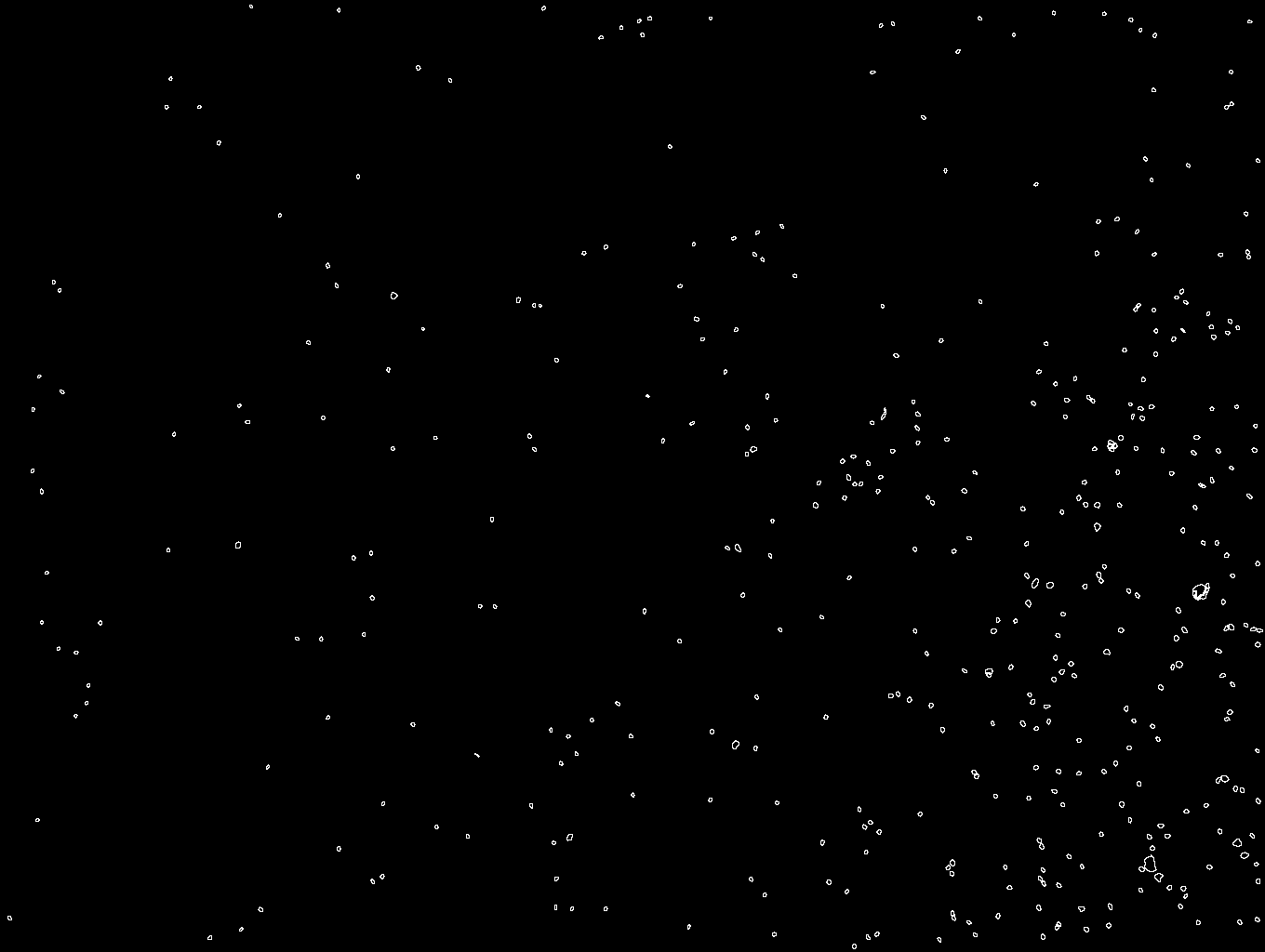

Supplement: Figure 2—figure supplement 2—source data 1. [file elife-38187-fig2-figsupp2-data1.zip › Figure 2 - figure suppl 2 - source data/Exp4/Plate A/Segmentation/r04c07f01p01-cell_outline.tif.tif]

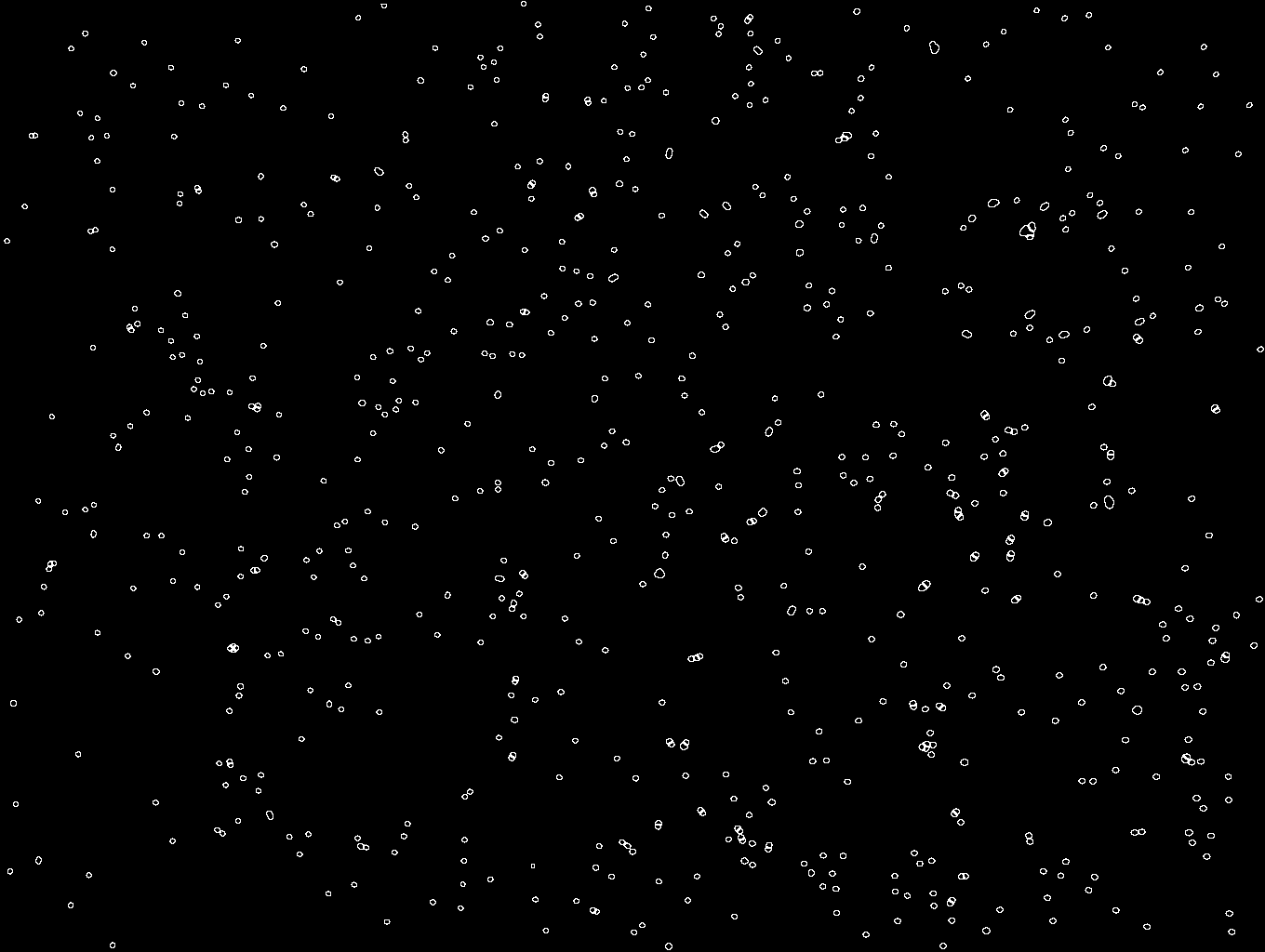

Supplement: Figure 2—figure supplement 2—source data 1. [file elife-38187-fig2-figsupp2-data1.zip › Figure 2 - figure suppl 2 - source data/Exp4/Plate A/Segmentation/r04c08f01p01-beads_outline.tif.tif]

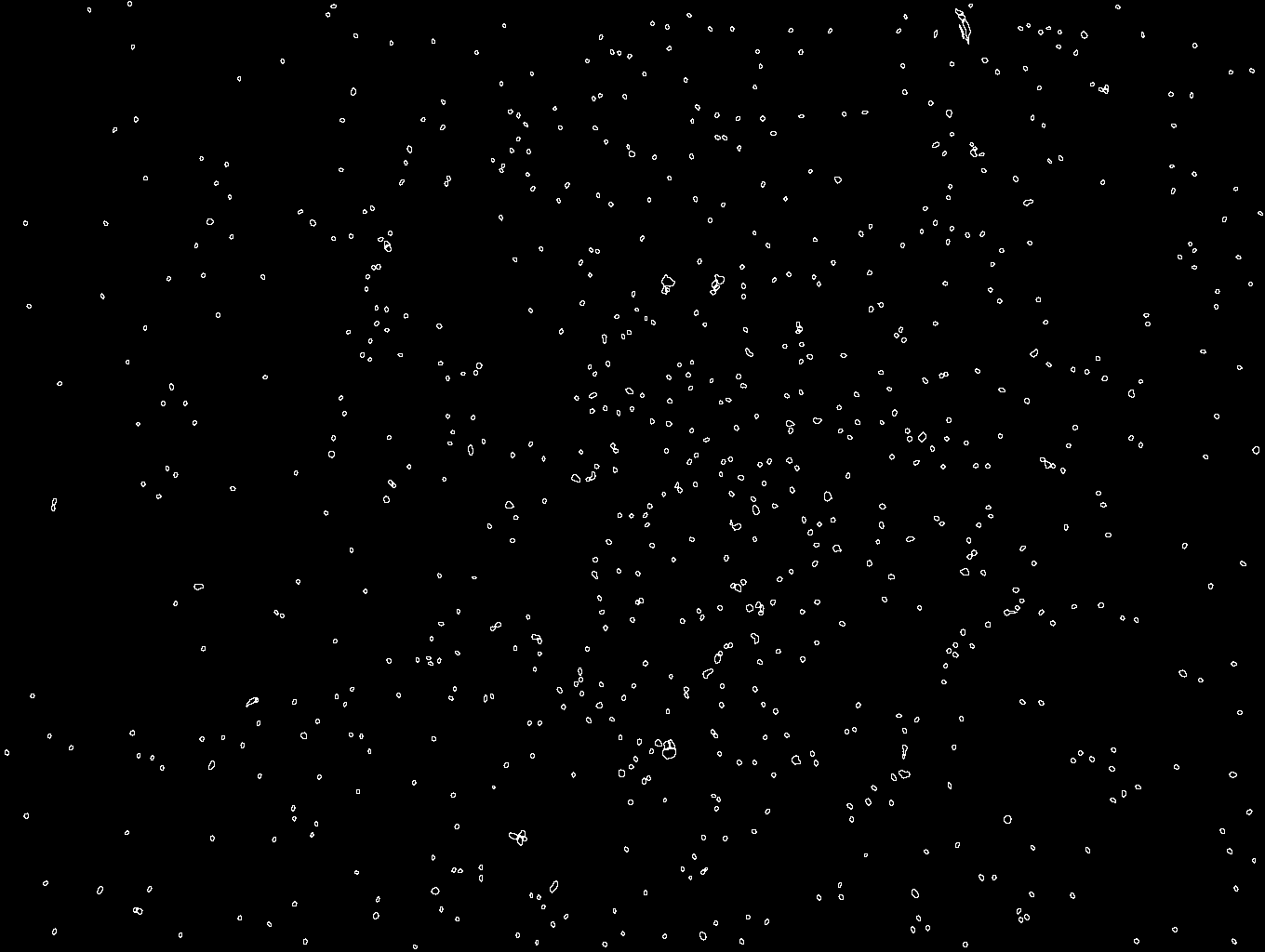

Supplement: Figure 2—figure supplement 2—source data 1. [file elife-38187-fig2-figsupp2-data1.zip › Figure 2 - figure suppl 2 - source data/Exp4/Plate A/Segmentation/r04c08f01p01-cell_outline.tif.tif]

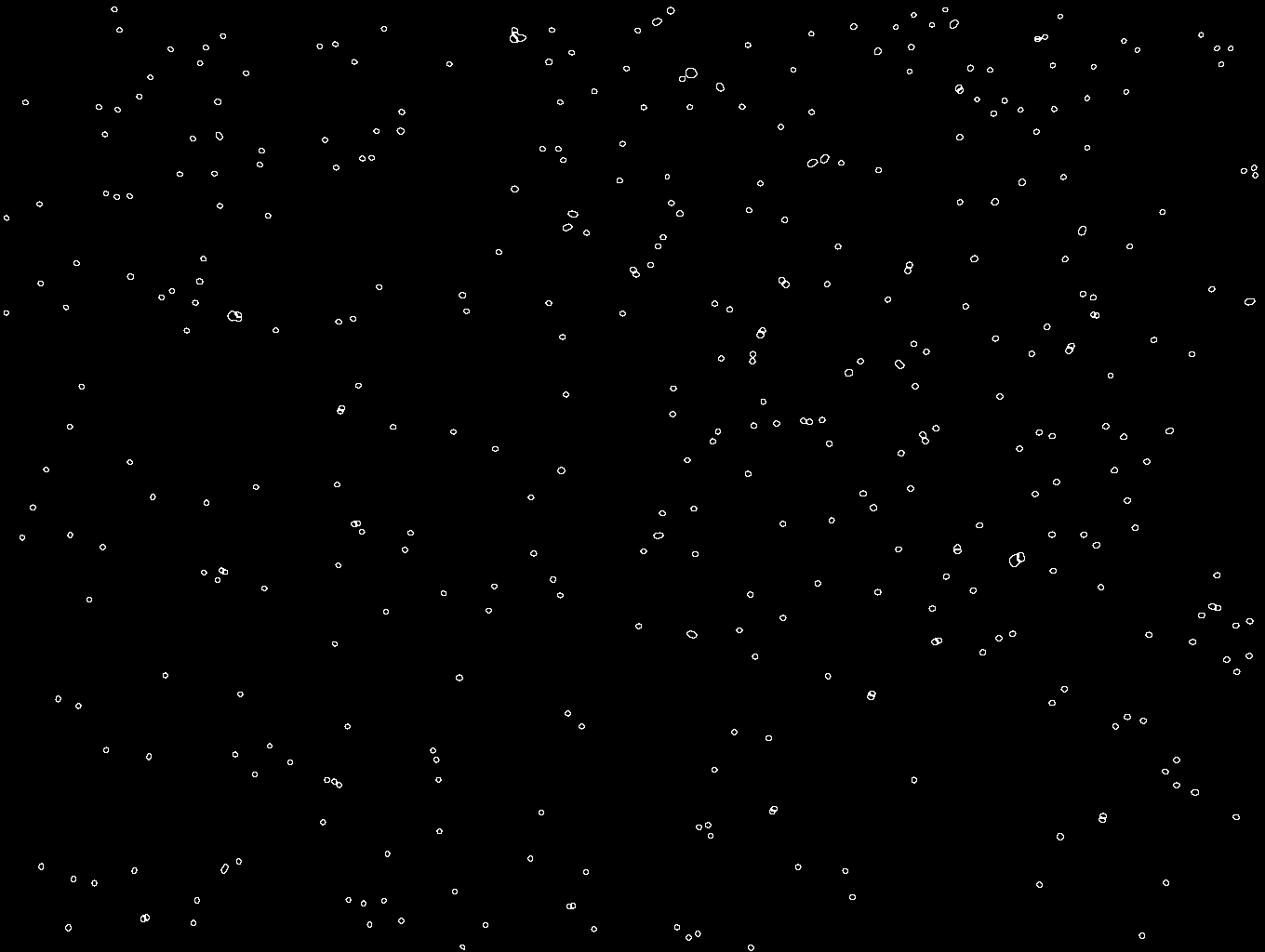

Supplement: Figure 2—figure supplement 2—source data 1. [file elife-38187-fig2-figsupp2-data1.zip › Figure 2 - figure suppl 2 - source data/Exp4/Plate A/Segmentation/r04c08f16p01-beads_outline.tif.tif]

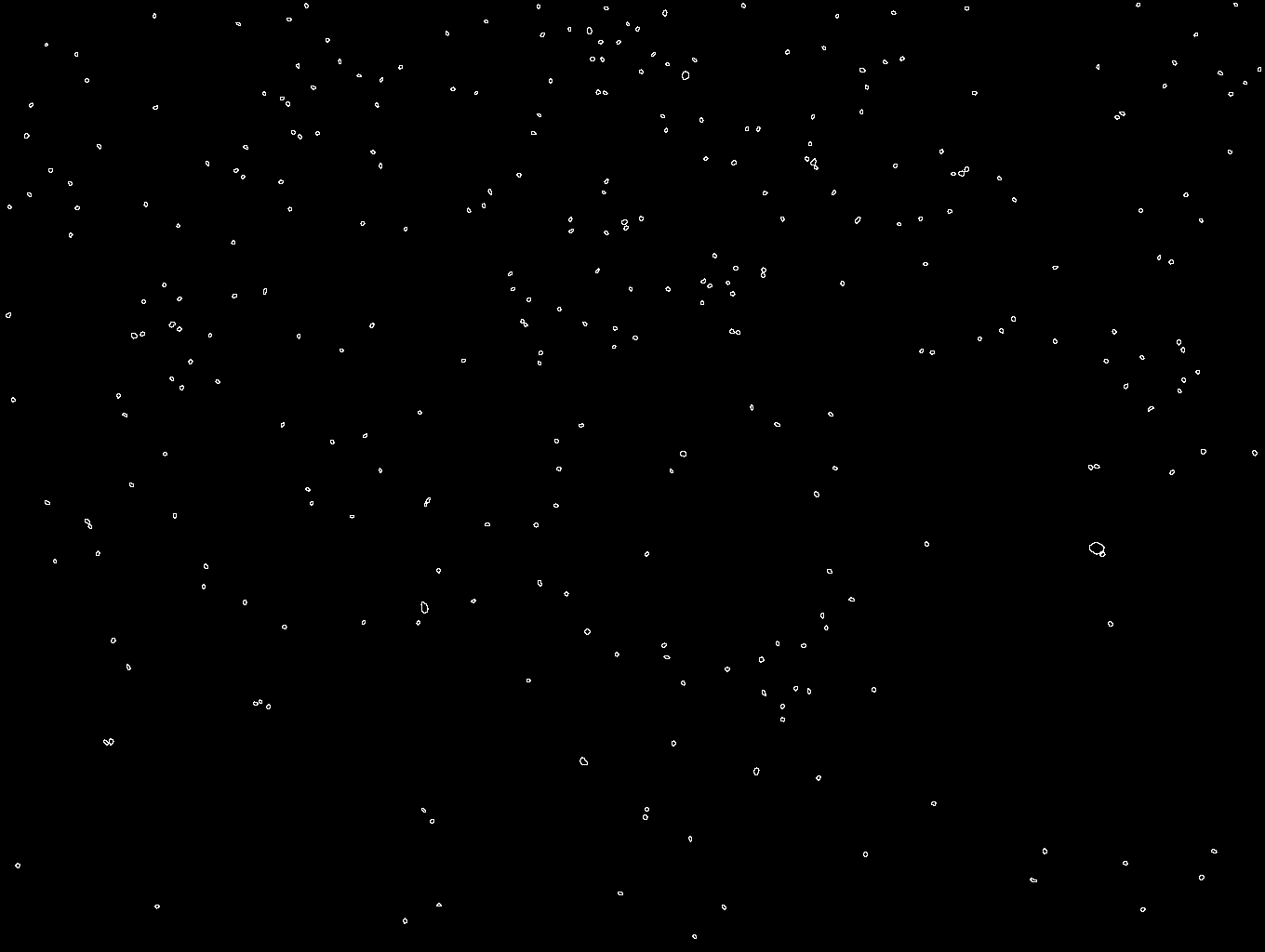

Supplement: Figure 2—figure supplement 2—source data 1. [file elife-38187-fig2-figsupp2-data1.zip › Figure 2 - figure suppl 2 - source data/Exp4/Plate A/Segmentation/r04c08f16p01-cell_outline.tif.tif]

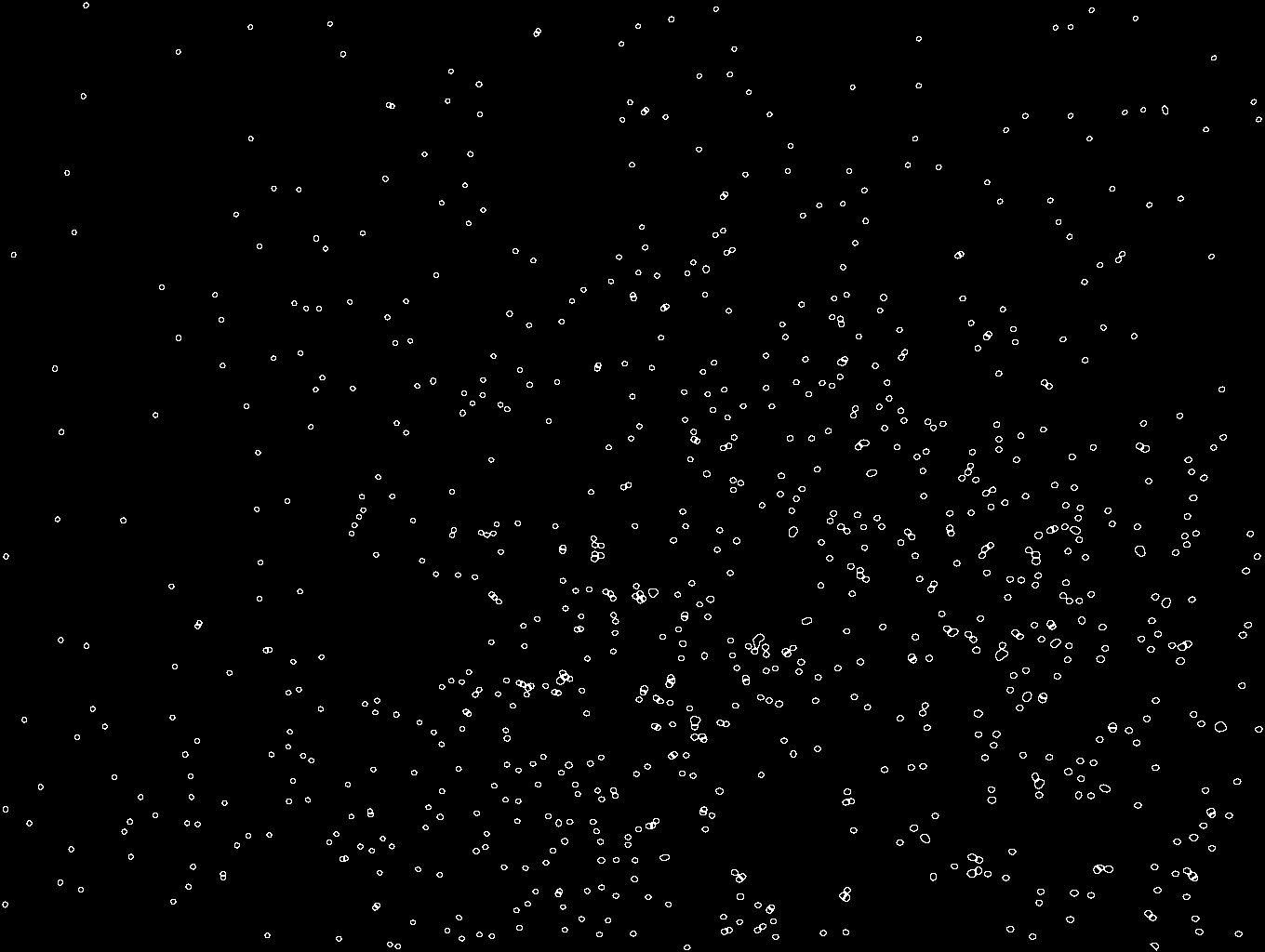

Supplement: Figure 2—figure supplement 2—source data 1. [file elife-38187-fig2-figsupp2-data1.zip › Figure 2 - figure suppl 2 - source data/Exp4/Plate A/Segmentation/r05c05f01p01-beads_outline.tif.tif]

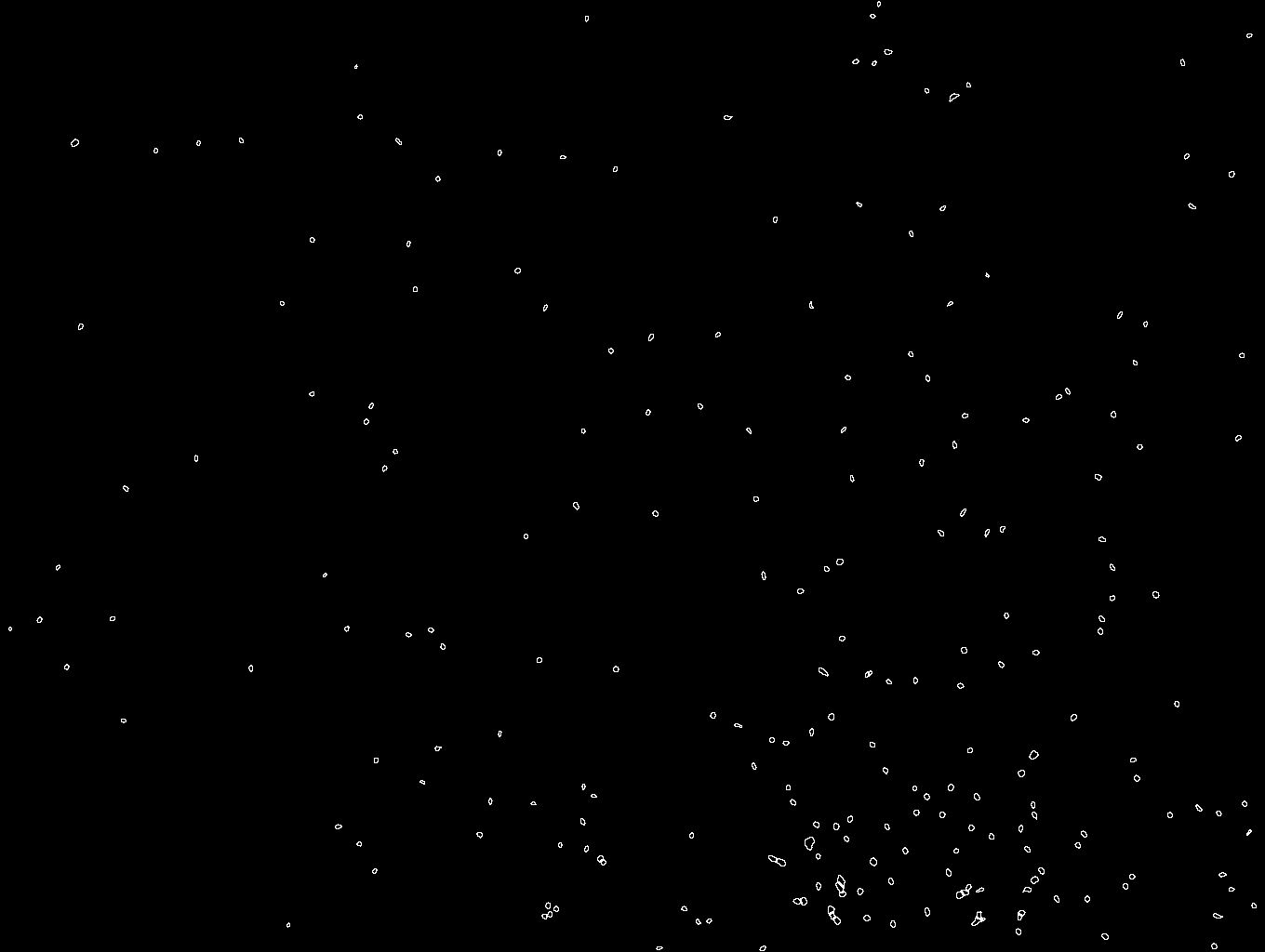

Supplement: Figure 2—figure supplement 2—source data 1. [file elife-38187-fig2-figsupp2-data1.zip › Figure 2 - figure suppl 2 - source data/Exp4/Plate A/Segmentation/r05c05f01p01-cell_outline.tif.tif]

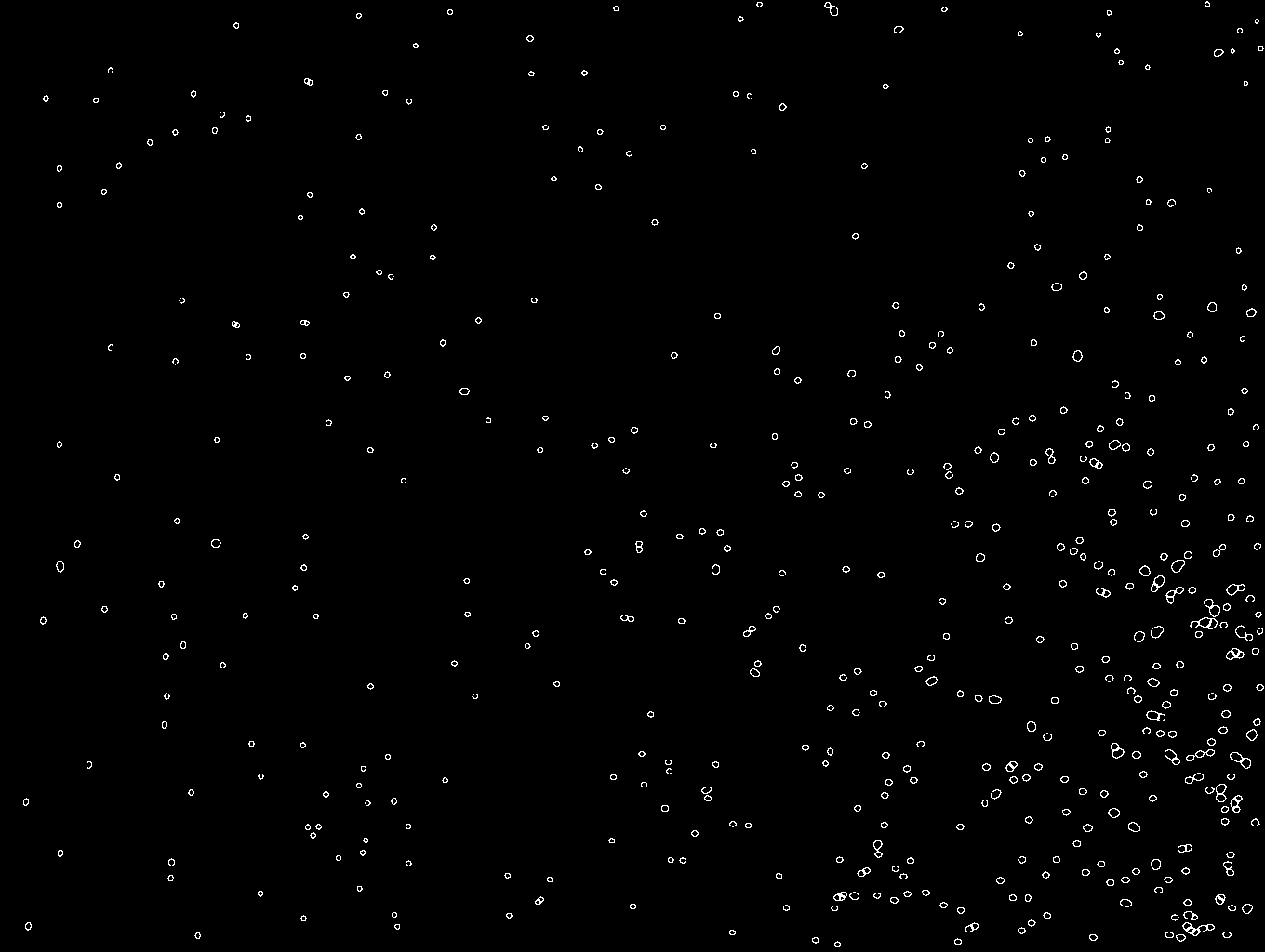

Supplement: Figure 2—figure supplement 2—source data 1. [file elife-38187-fig2-figsupp2-data1.zip › Figure 2 - figure suppl 2 - source data/Exp4/Plate A/Segmentation/r06c04f01p01-beads_outline.tif.tif]

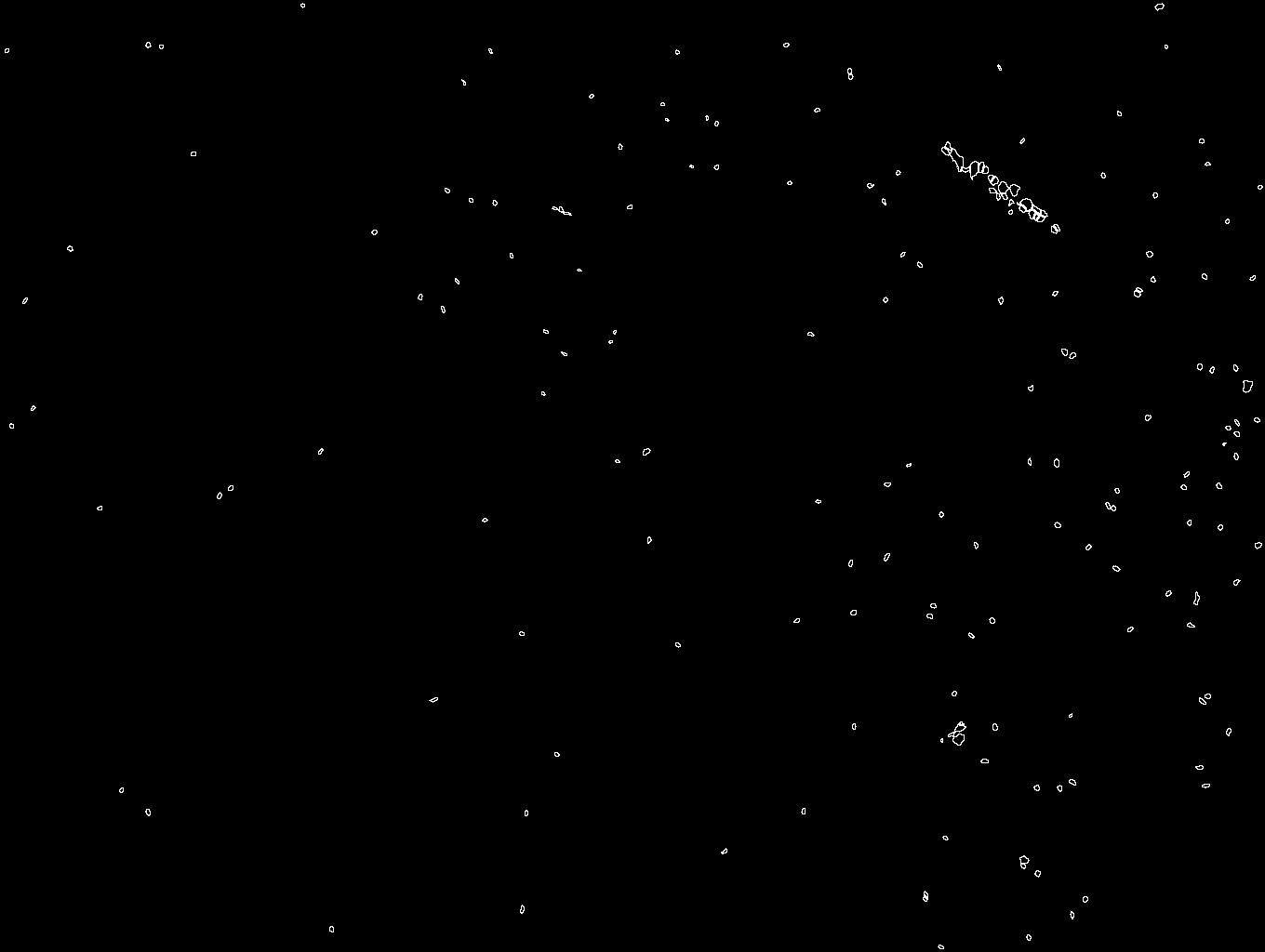

Supplement: Figure 2—figure supplement 2—source data 1. [file elife-38187-fig2-figsupp2-data1.zip › Figure 2 - figure suppl 2 - source data/Exp4/Plate A/Segmentation/r06c04f01p01-cell_outline.tif.tif]

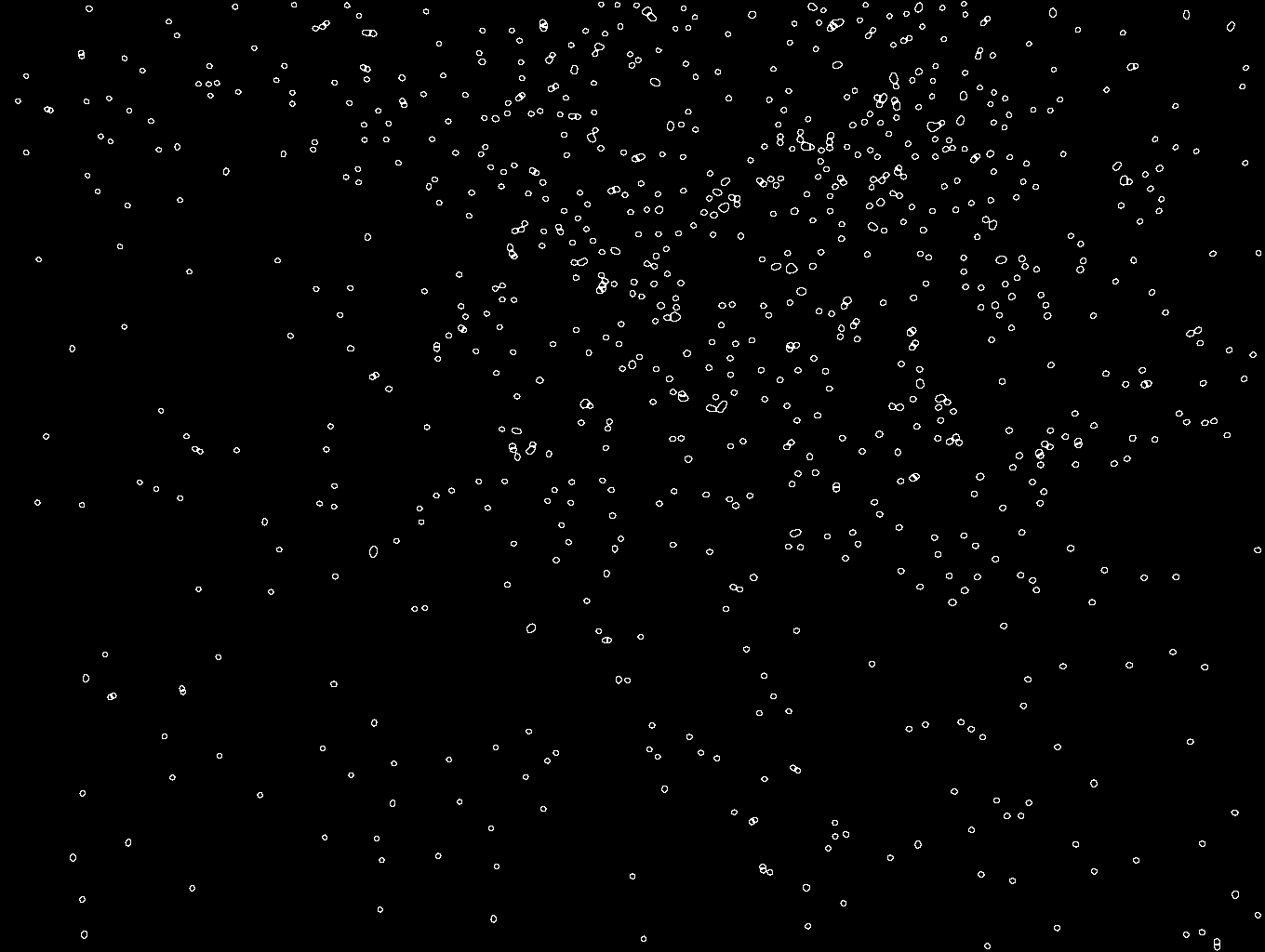

Supplement: Figure 2—figure supplement 2—source data 1. [file elife-38187-fig2-figsupp2-data1.zip › Figure 2 - figure suppl 2 - source data/Exp4/Plate A/Segmentation/r06c06f01p01-beads_outline.tif.tif]

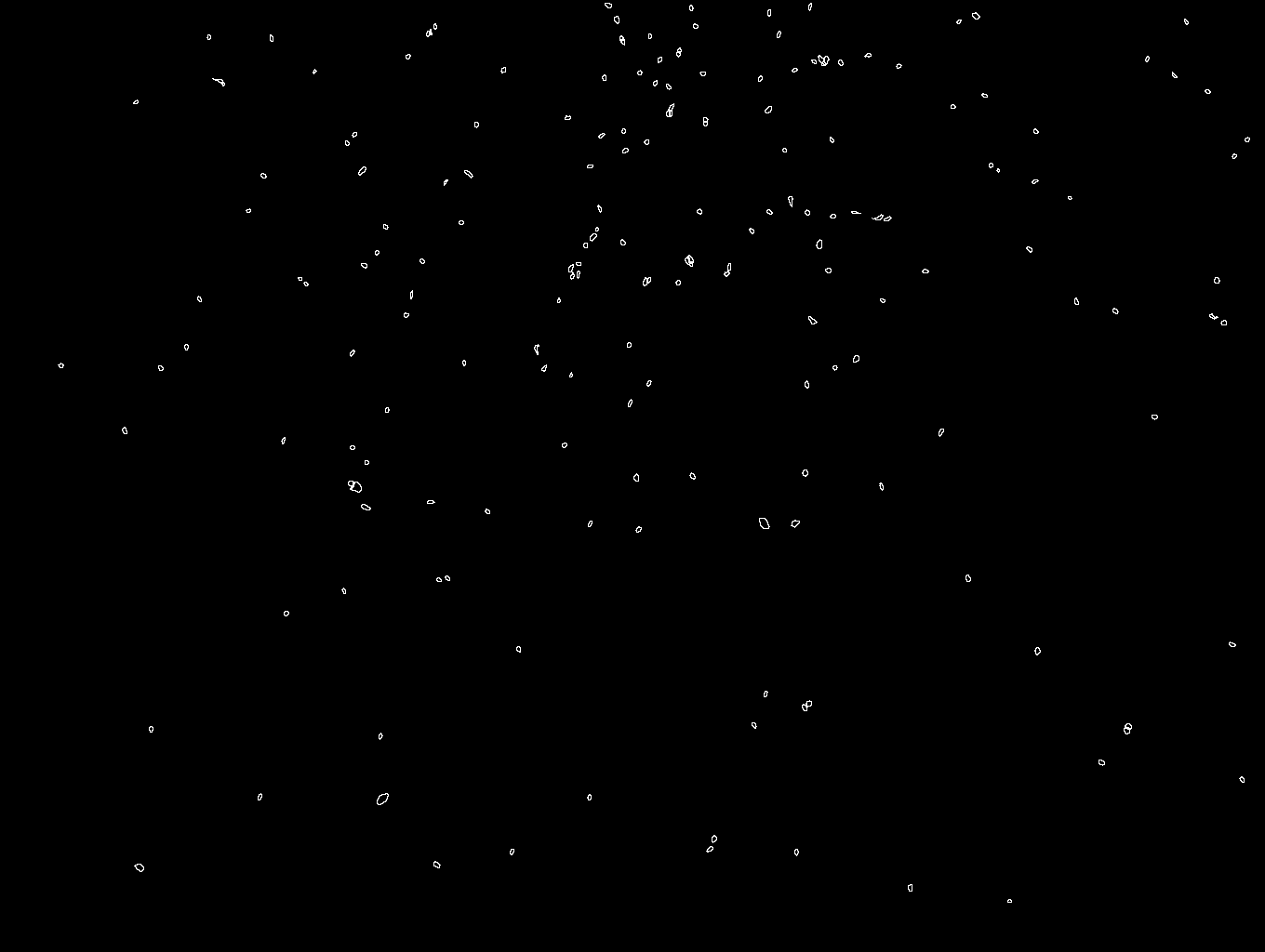

Supplement: Figure 2—figure supplement 2—source data 1. [file elife-38187-fig2-figsupp2-data1.zip › Figure 2 - figure suppl 2 - source data/Exp4/Plate A/Segmentation/r06c06f01p01-cell_outline.tif.tif]

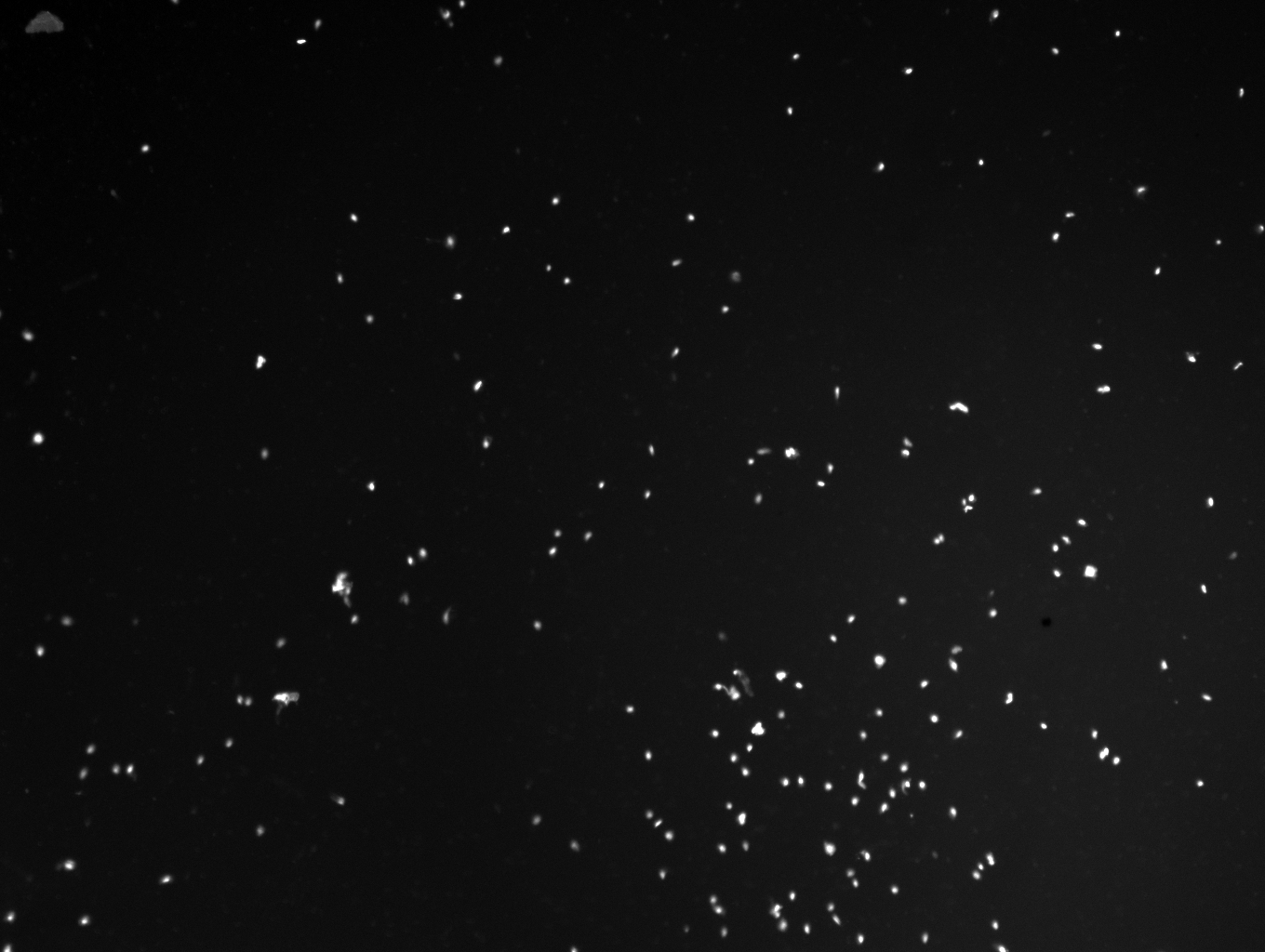

Supplement: Figure 2—figure supplement 2—source data 1. [file elife-38187-fig2-figsupp2-data1.zip › Figure 2 - figure suppl 2 - source data/Exp4/Plate B/r02c02f01p01-ch1sk1fk1fl1.tiff]

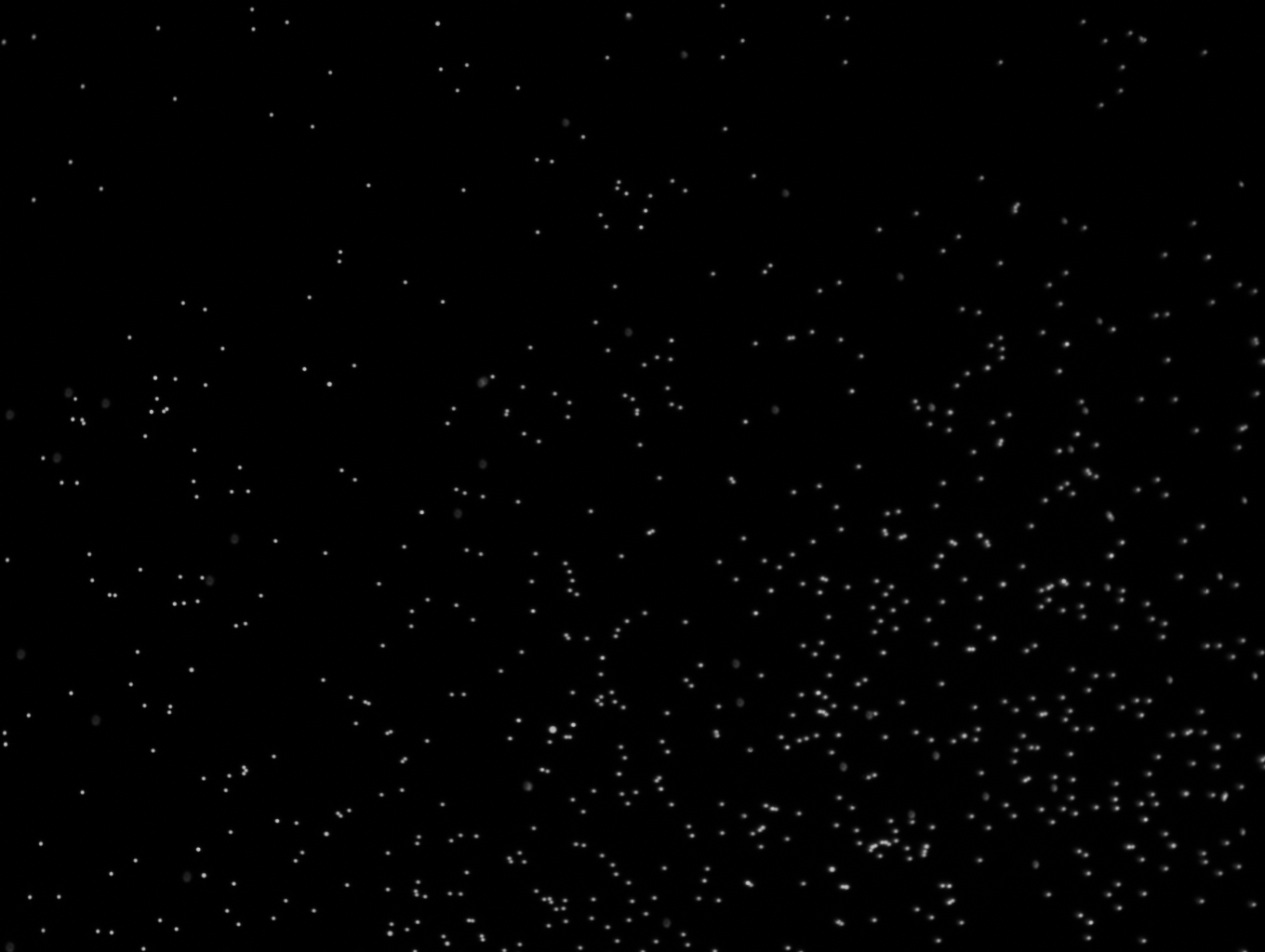

Supplement: Figure 2—figure supplement 2—source data 1. [file elife-38187-fig2-figsupp2-data1.zip › Figure 2 - figure suppl 2 - source data/Exp4/Plate B/r02c02f01p01-ch2sk1fk1fl1.tiff]

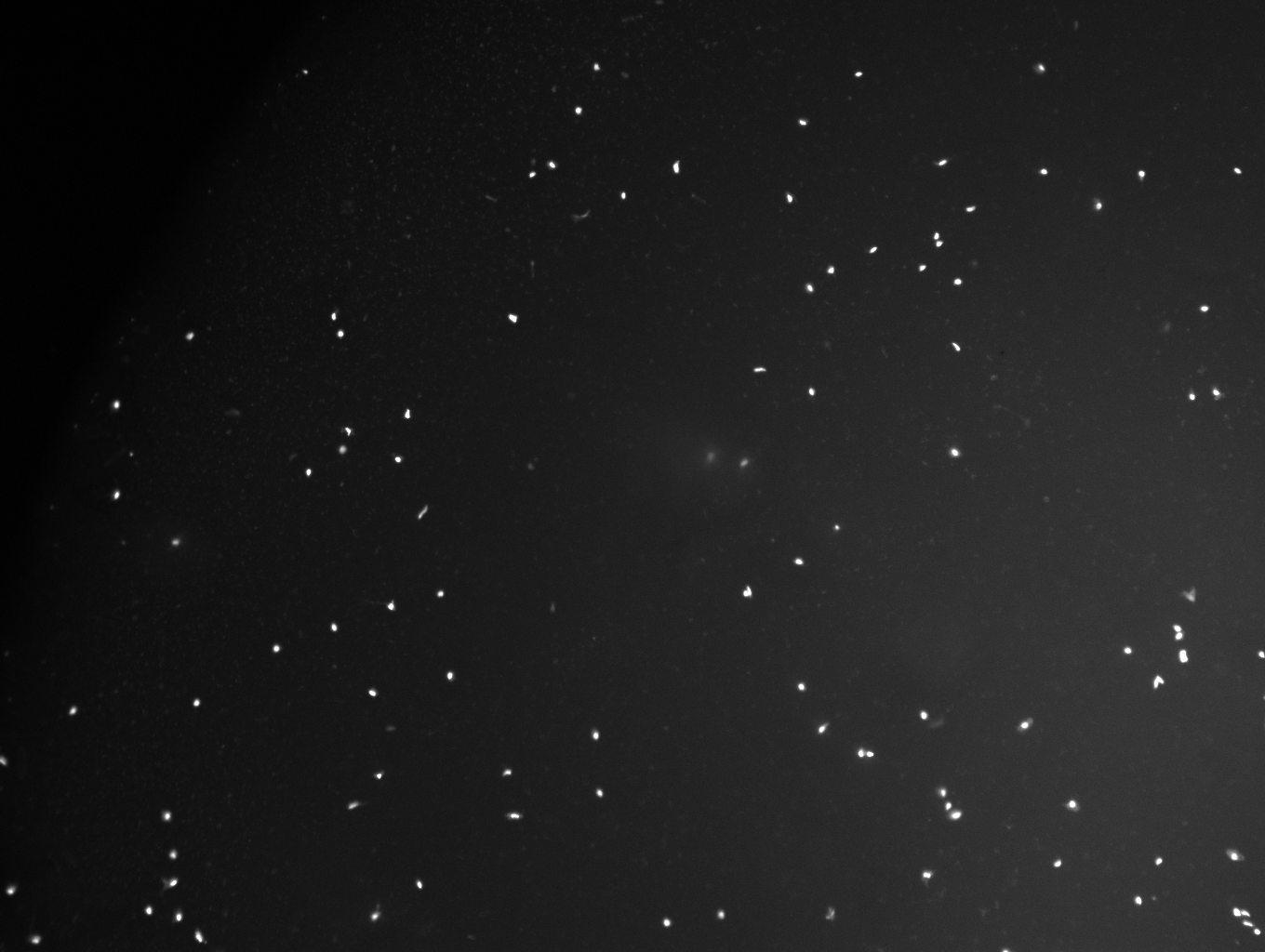

Supplement: Figure 2—figure supplement 2—source data 1. [file elife-38187-fig2-figsupp2-data1.zip › Figure 2 - figure suppl 2 - source data/Exp4/Plate B/r02c08f01p01-ch1sk1fk1fl1.tiff]

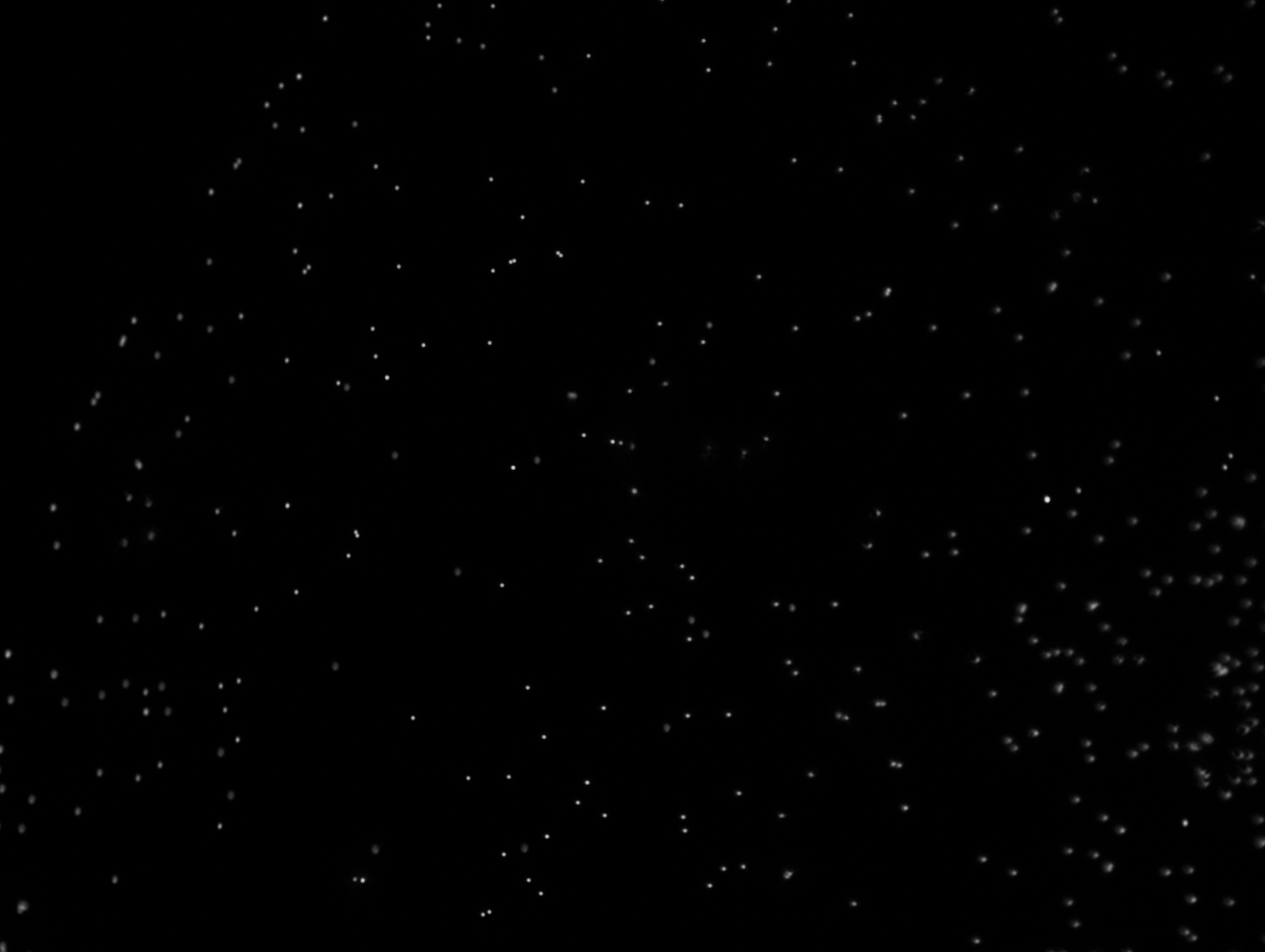

Supplement: Figure 2—figure supplement 2—source data 1. [file elife-38187-fig2-figsupp2-data1.zip › Figure 2 - figure suppl 2 - source data/Exp4/Plate B/r02c08f01p01-ch2sk1fk1fl1.tiff]

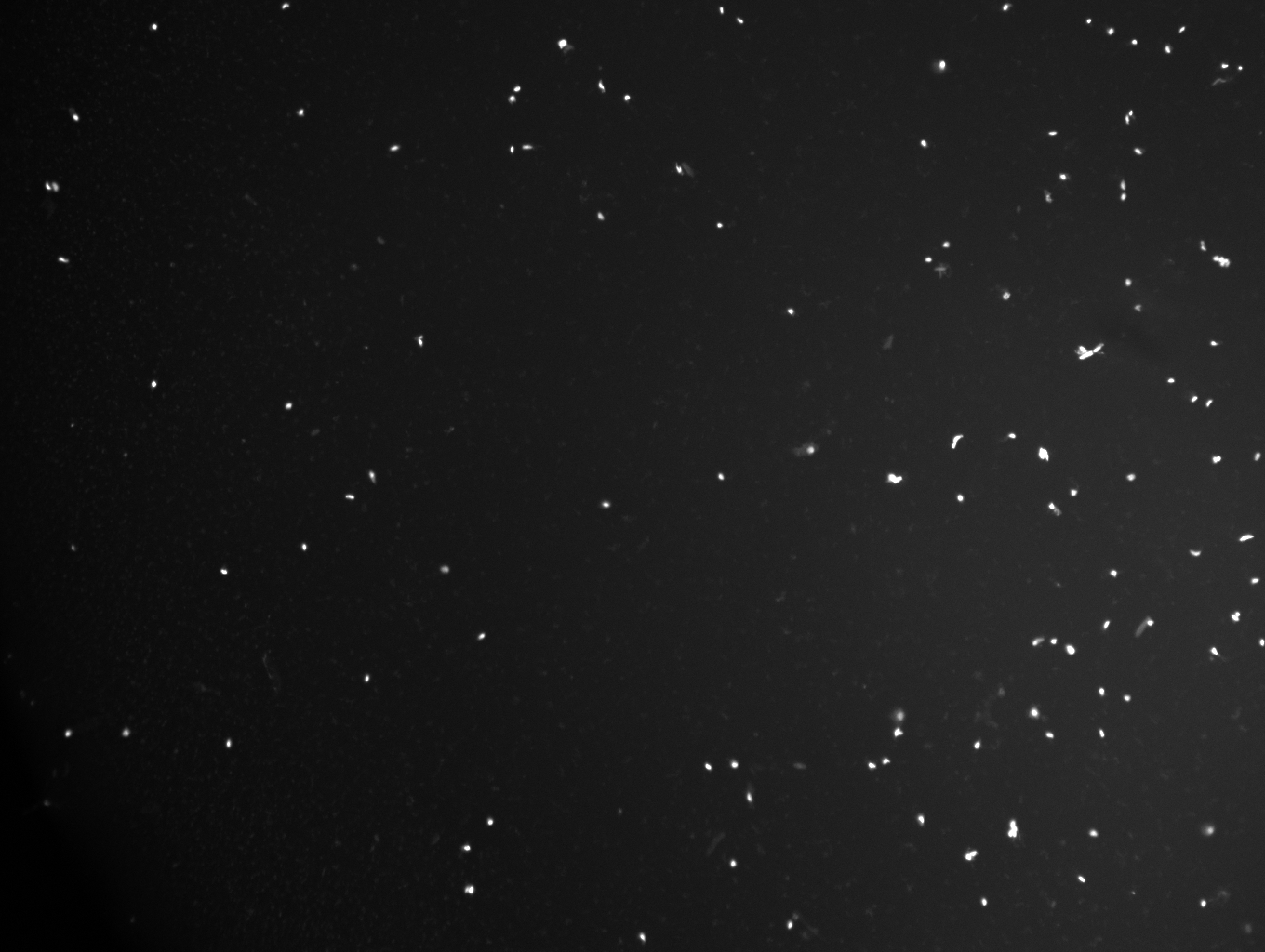

Supplement: Figure 2—figure supplement 2—source data 1. [file elife-38187-fig2-figsupp2-data1.zip › Figure 2 - figure suppl 2 - source data/Exp4/Plate B/r02c11f01p01-ch1sk1fk1fl1.tiff]

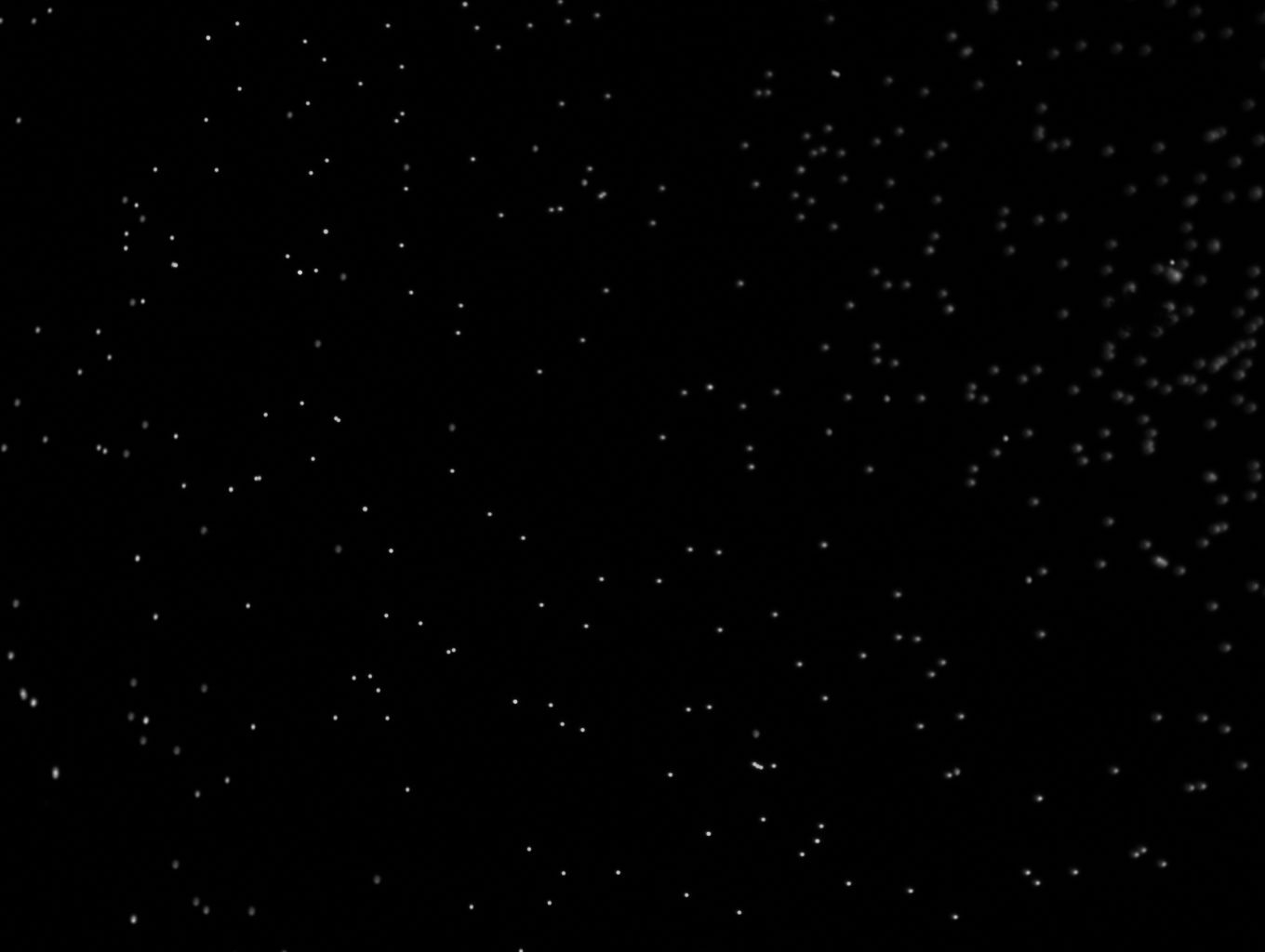

Supplement: Figure 2—figure supplement 2—source data 1. [file elife-38187-fig2-figsupp2-data1.zip › Figure 2 - figure suppl 2 - source data/Exp4/Plate B/r02c11f01p01-ch2sk1fk1fl1.tiff]

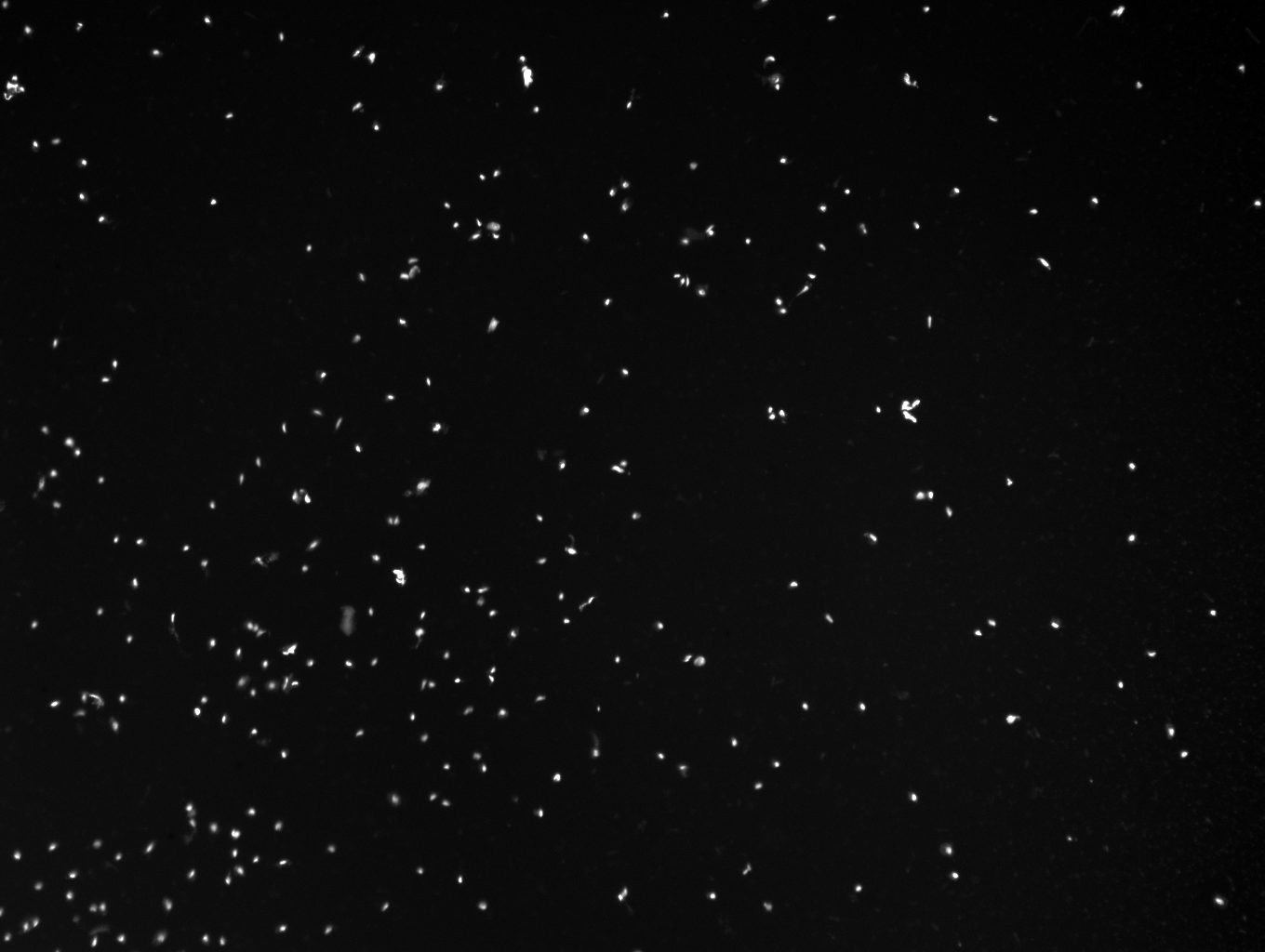

Supplement: Figure 2—figure supplement 2—source data 1. [file elife-38187-fig2-figsupp2-data1.zip › Figure 2 - figure suppl 2 - source data/Exp4/Plate B/r03c02f01p01-ch1sk1fk1fl1.tiff]

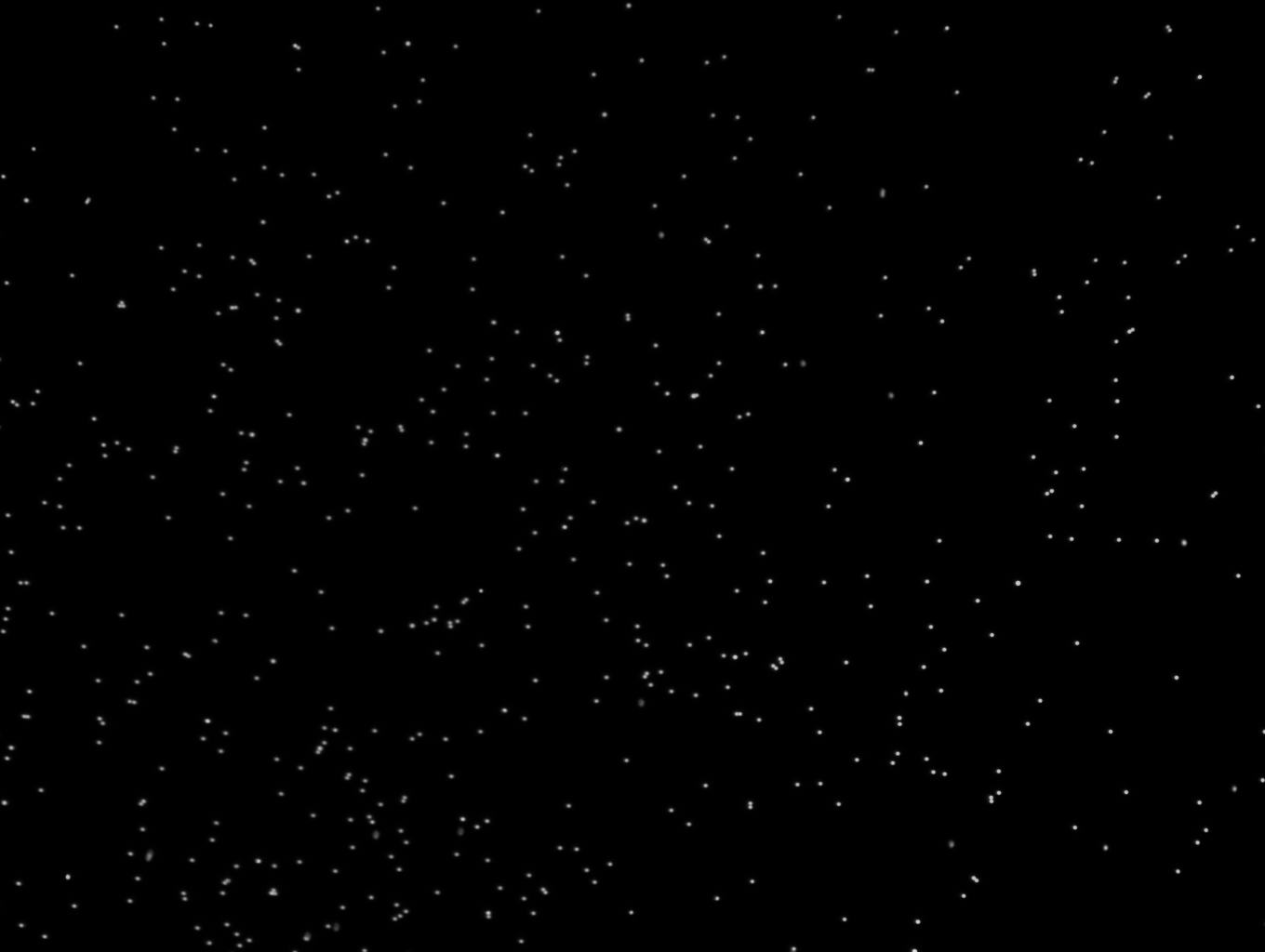

Supplement: Figure 2—figure supplement 2—source data 1. [file elife-38187-fig2-figsupp2-data1.zip › Figure 2 - figure suppl 2 - source data/Exp4/Plate B/r03c02f01p01-ch2sk1fk1fl1.tiff]

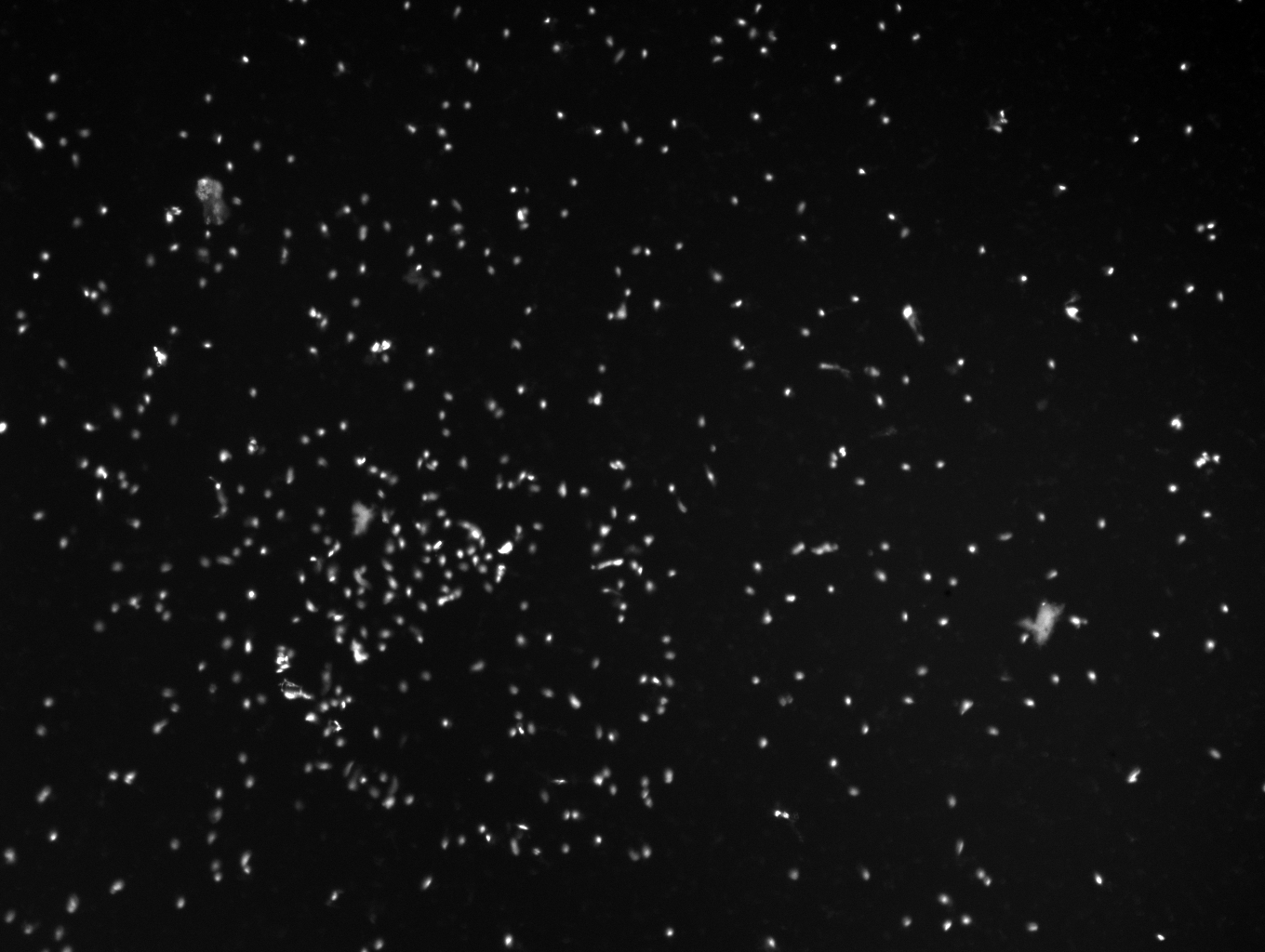

Supplement: Figure 2—figure supplement 2—source data 1. [file elife-38187-fig2-figsupp2-data1.zip › Figure 2 - figure suppl 2 - source data/Exp4/Plate B/r03c04f01p01-ch1sk1fk1fl1.tiff]

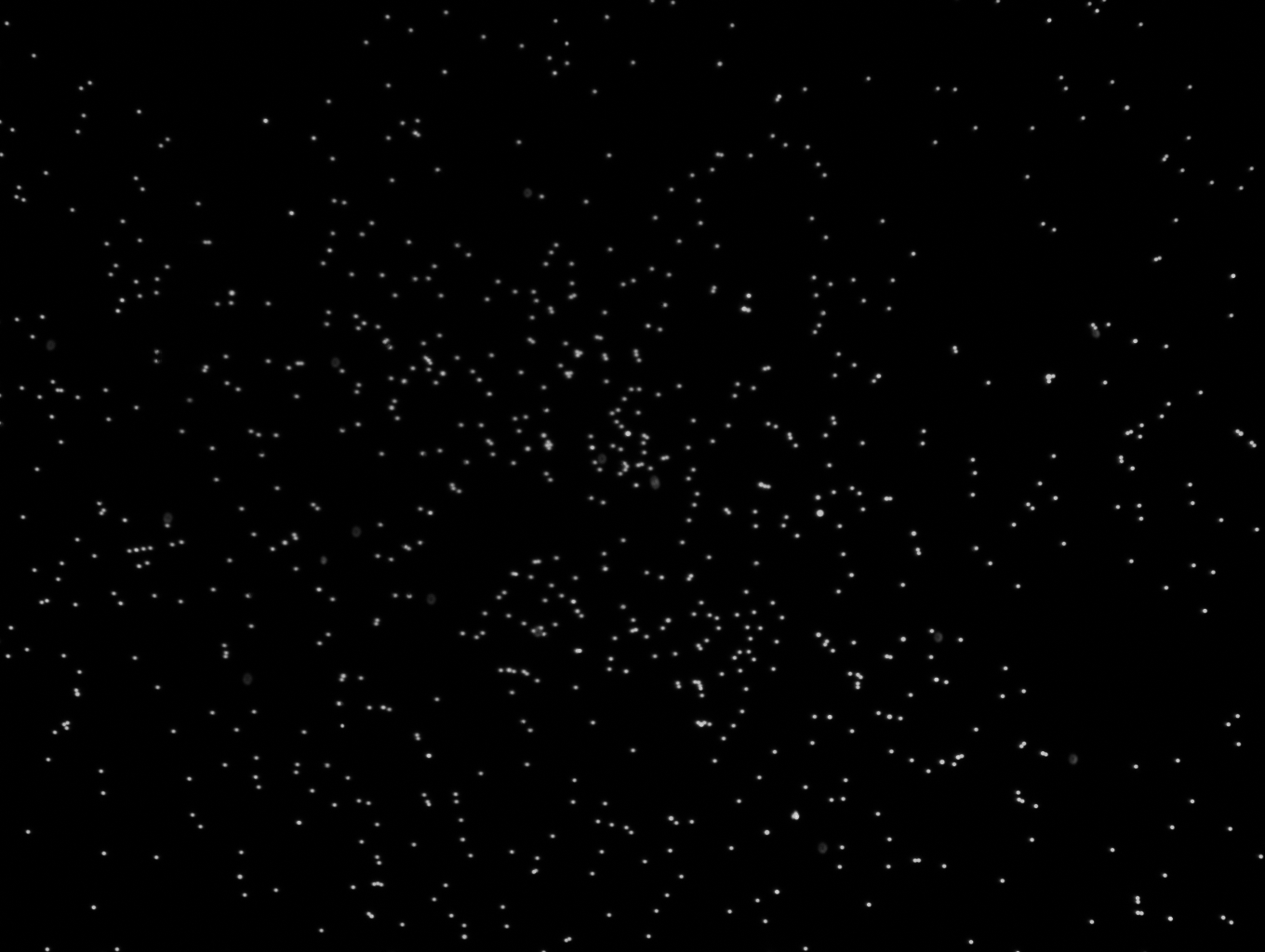

Supplement: Figure 2—figure supplement 2—source data 1. [file elife-38187-fig2-figsupp2-data1.zip › Figure 2 - figure suppl 2 - source data/Exp4/Plate B/r03c04f01p01-ch2sk1fk1fl1.tiff]

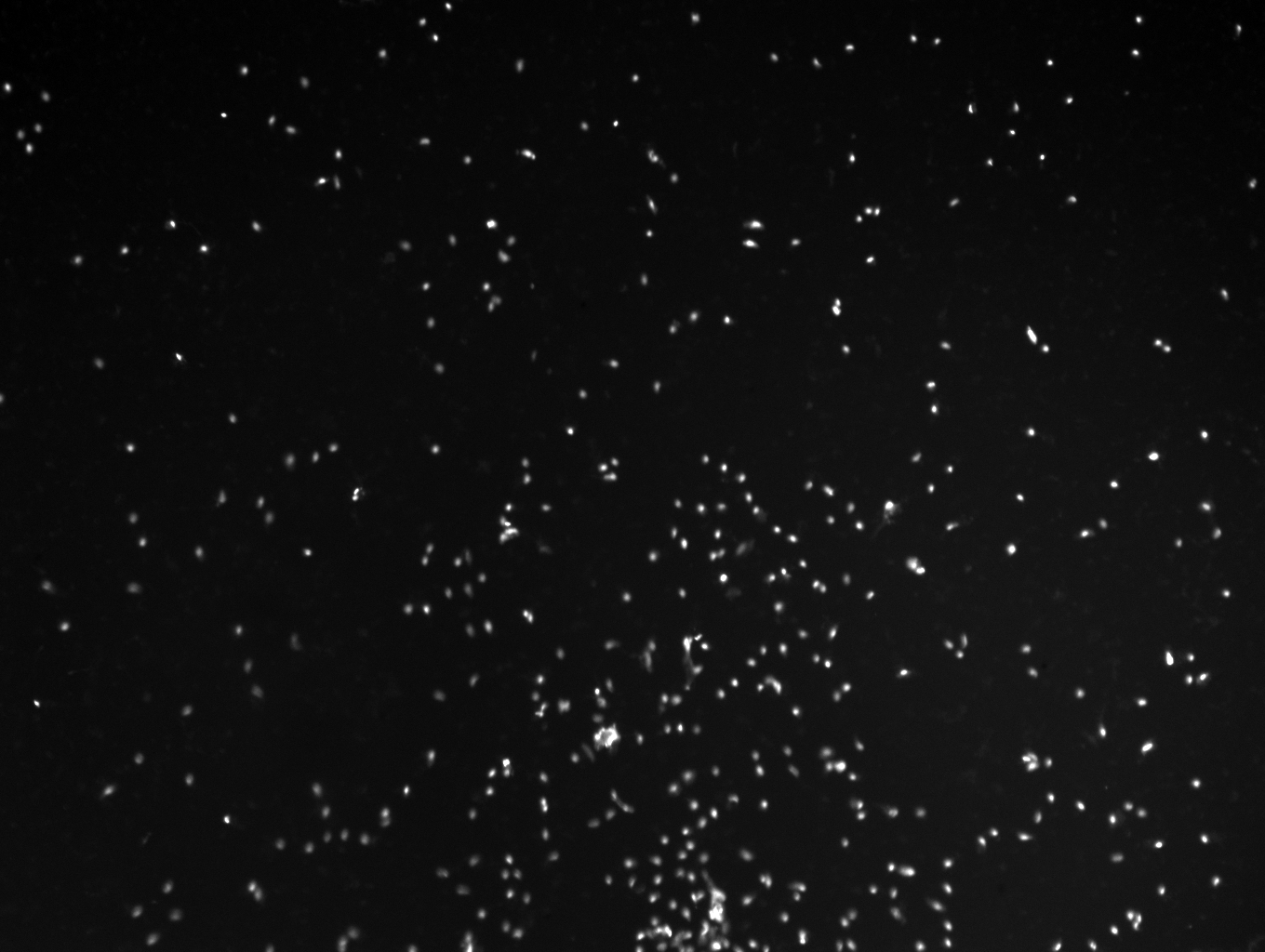

Supplement: Figure 2—figure supplement 2—source data 1. [file elife-38187-fig2-figsupp2-data1.zip › Figure 2 - figure suppl 2 - source data/Exp4/Plate B/r03c07f01p01-ch1sk1fk1fl1.tiff]

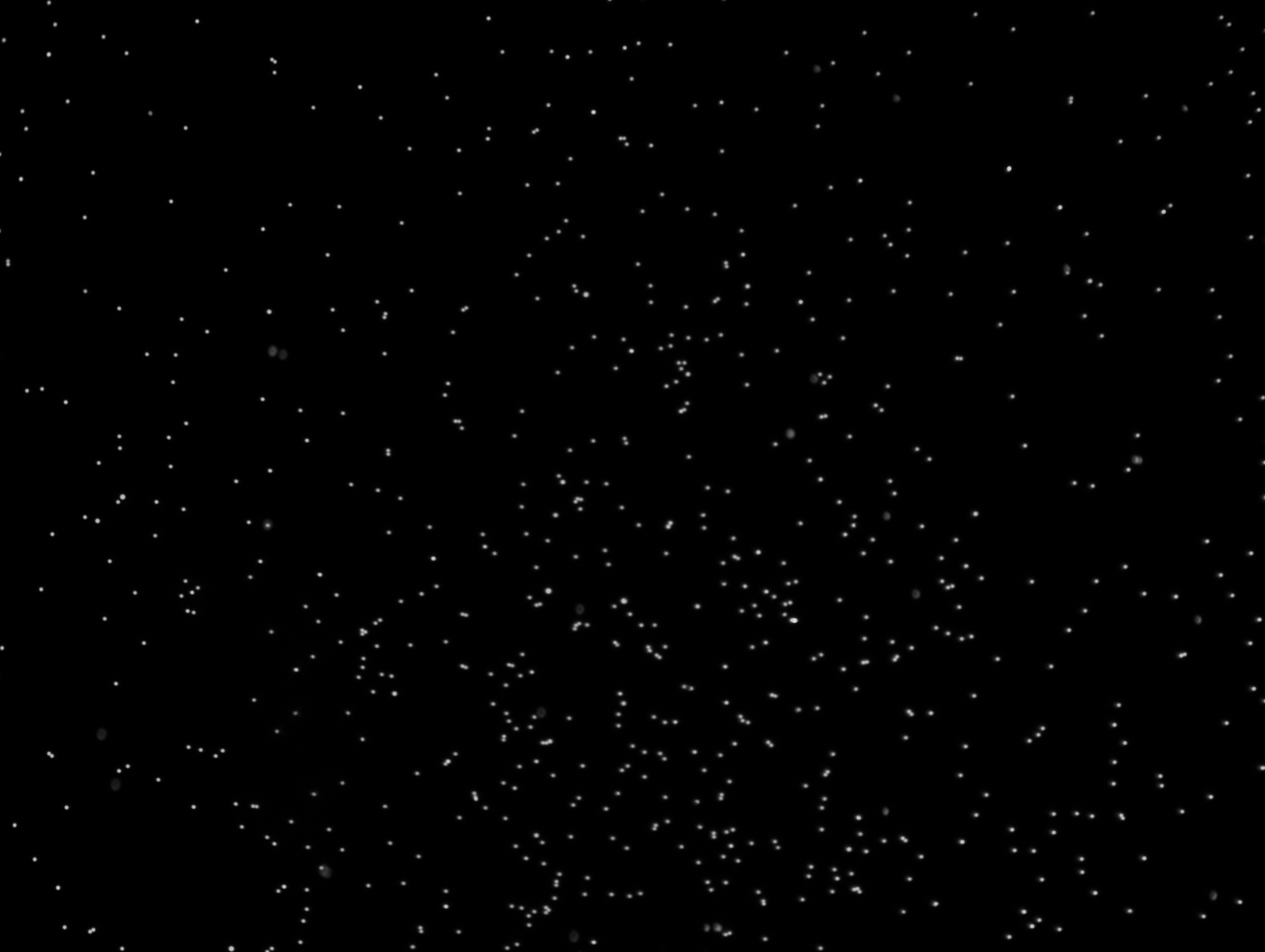

Supplement: Figure 2—figure supplement 2—source data 1. [file elife-38187-fig2-figsupp2-data1.zip › Figure 2 - figure suppl 2 - source data/Exp4/Plate B/r03c07f01p01-ch2sk1fk1fl1.tiff]

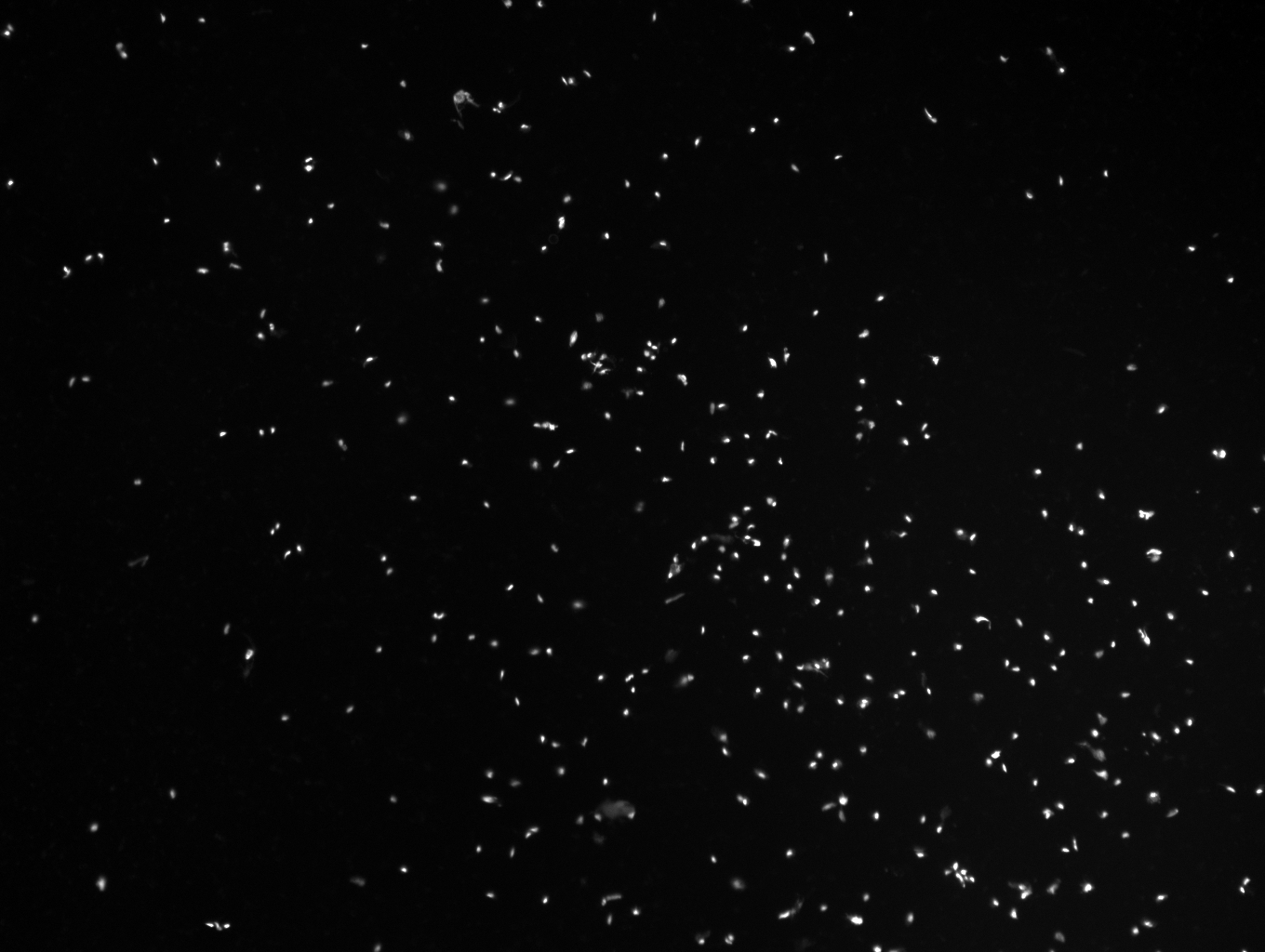

Supplement: Figure 2—figure supplement 2—source data 1. [file elife-38187-fig2-figsupp2-data1.zip › Figure 2 - figure suppl 2 - source data/Exp4/Plate B/r03c08f01p01-ch1sk1fk1fl1.tiff]

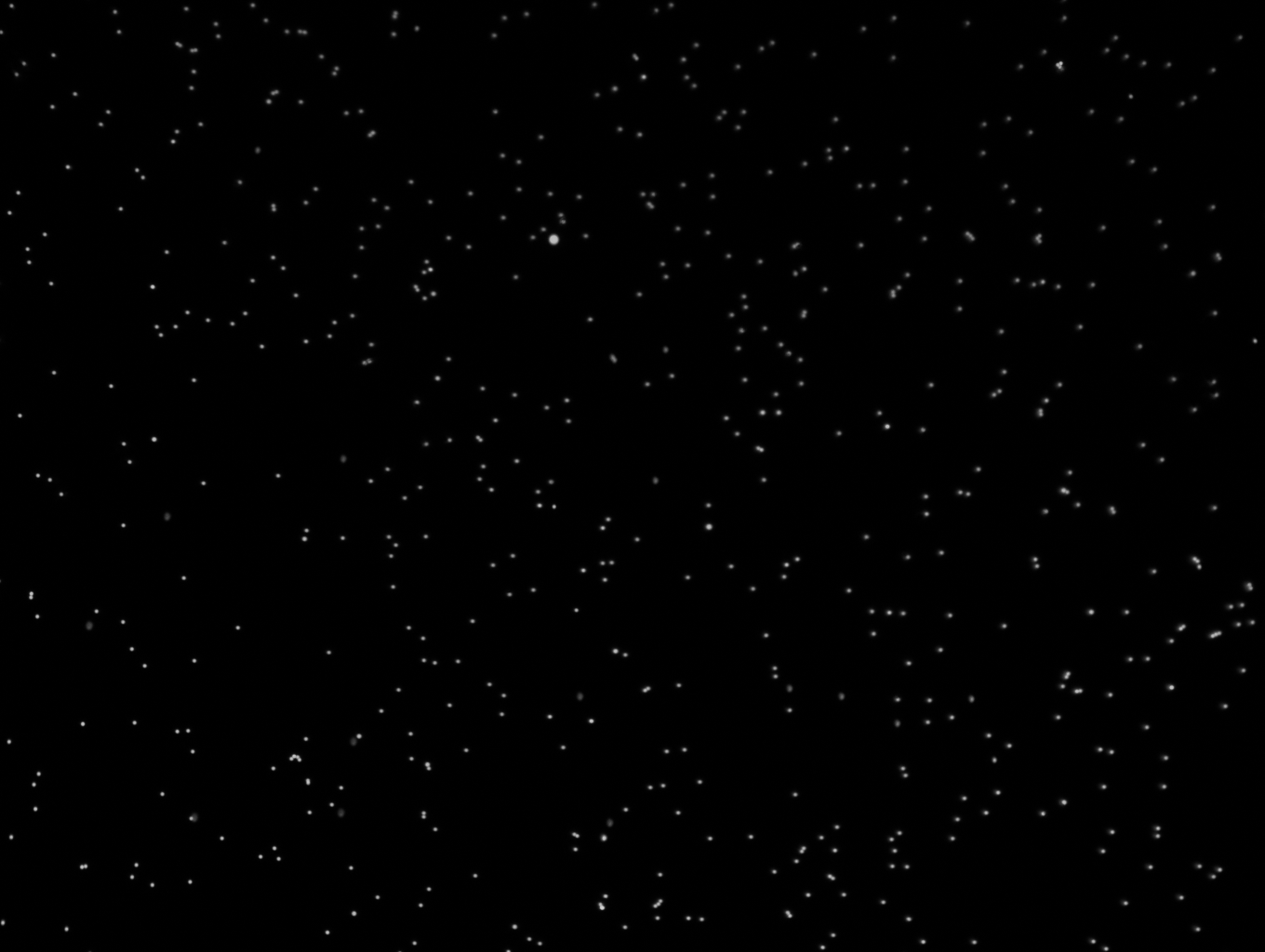

Supplement: Figure 2—figure supplement 2—source data 1. [file elife-38187-fig2-figsupp2-data1.zip › Figure 2 - figure suppl 2 - source data/Exp4/Plate B/r03c08f01p01-ch2sk1fk1fl1.tiff]

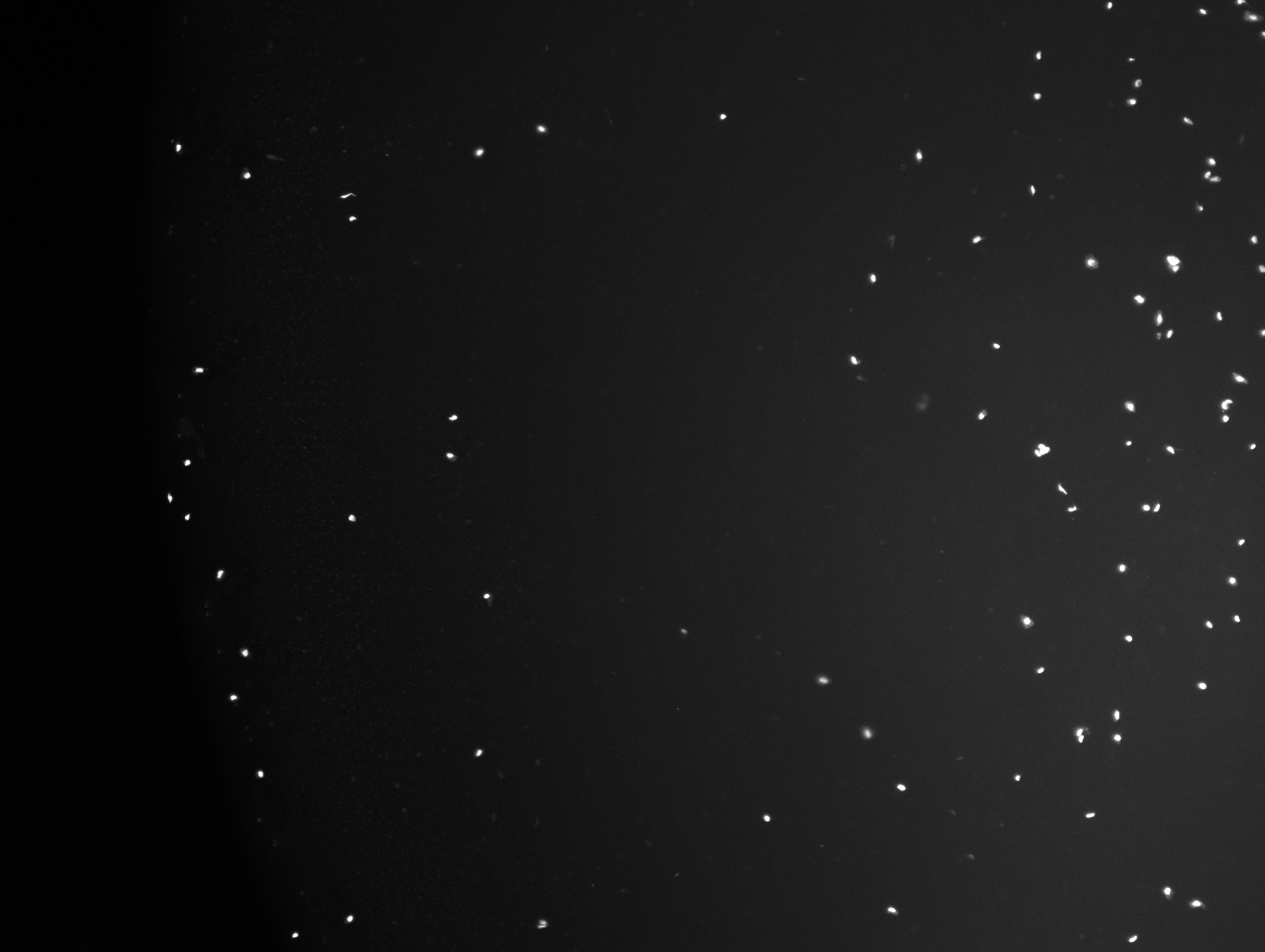

Supplement: Figure 2—figure supplement 2—source data 1. [file elife-38187-fig2-figsupp2-data1.zip › Figure 2 - figure suppl 2 - source data/Exp4/Plate B/r04c02f01p01-ch1sk1fk1fl1.tiff]

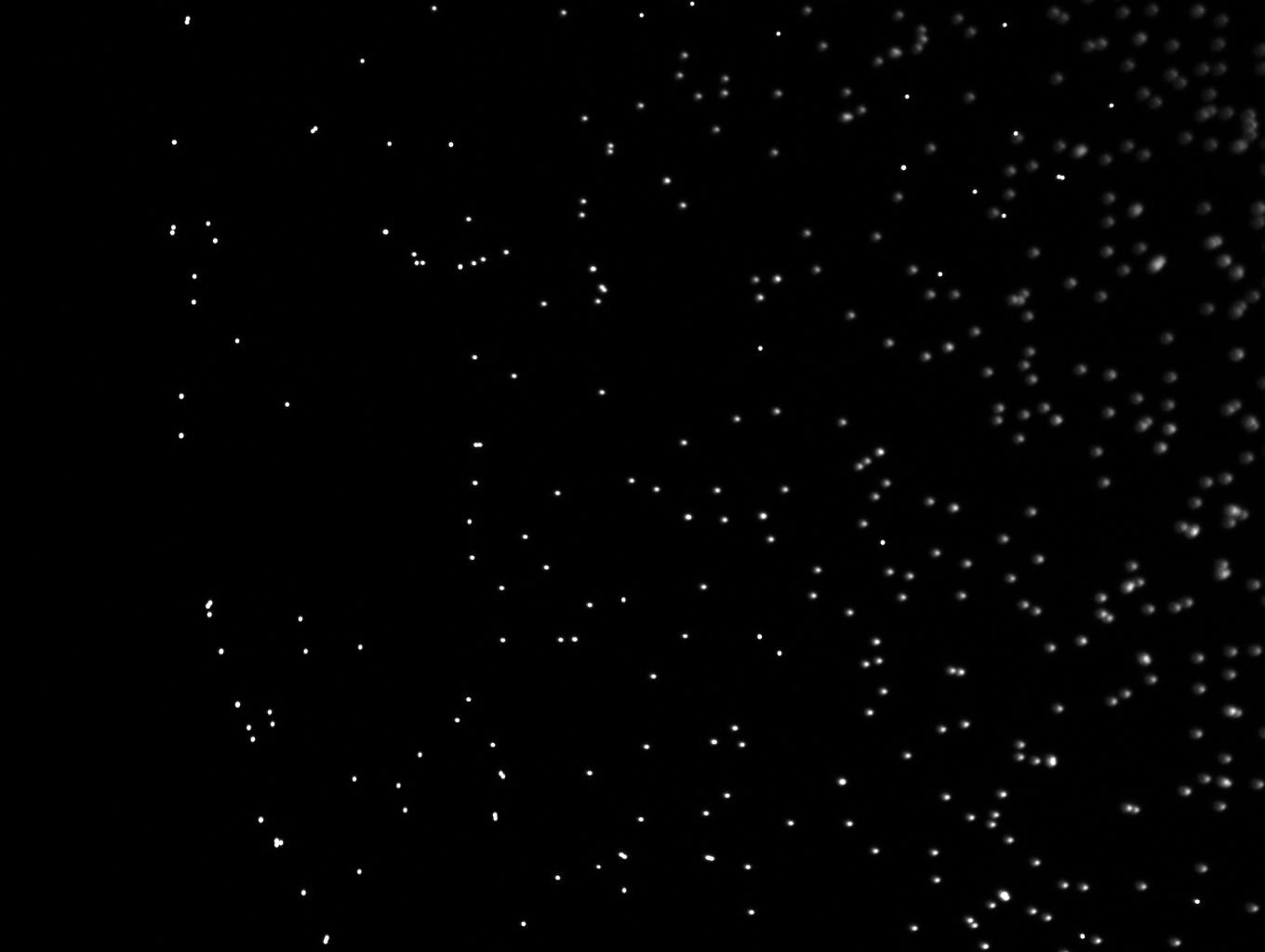

Supplement: Figure 2—figure supplement 2—source data 1. [file elife-38187-fig2-figsupp2-data1.zip › Figure 2 - figure suppl 2 - source data/Exp4/Plate B/r04c02f01p01-ch2sk1fk1fl1.tiff]

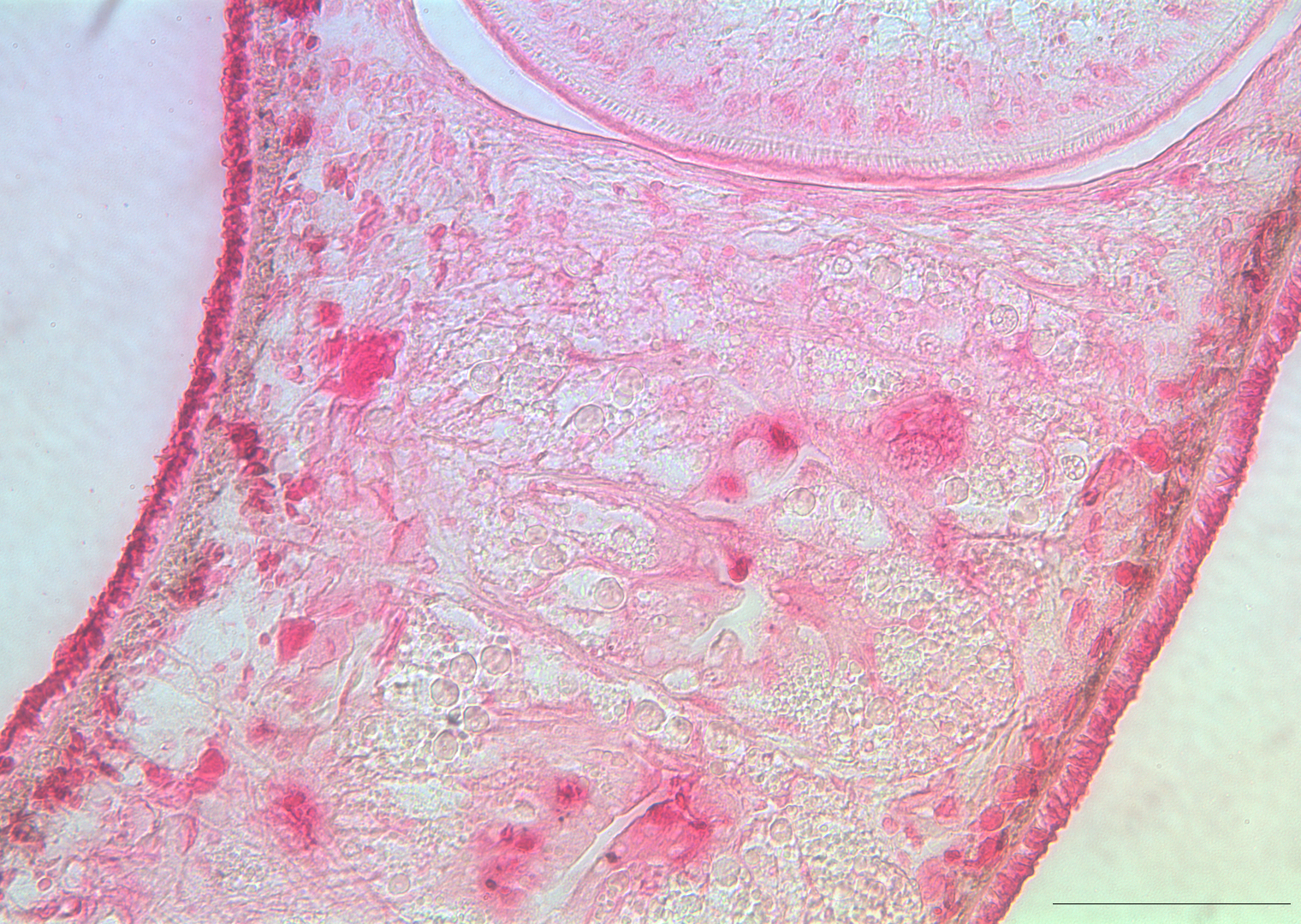

Supplement: Figure 4—source data 1. [file elife-38187-fig4-data1.zip › FIgure 4 - source data 1/Raw Images Glycogen/Large_AG_10x.tif]

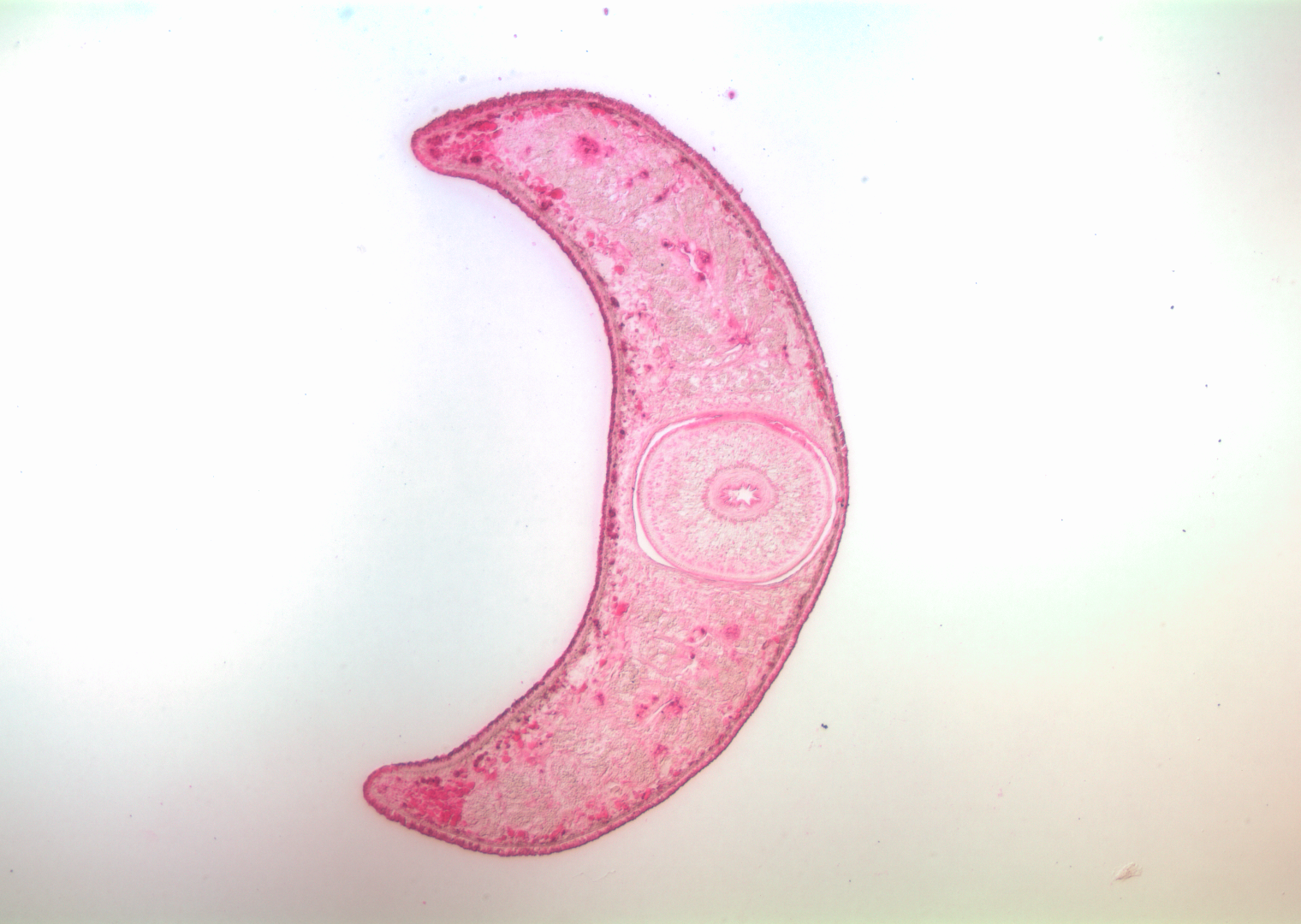

Supplement: Figure 4—source data 1. [file elife-38187-fig4-data1.zip › FIgure 4 - source data 1/Raw Images Glycogen/Large_AG_5x.tif]
