# Supplementary figures and images for: Body size-dependent energy storage causes Kleiber’s law scaling of the metabolic rate in planarians (part 3 of 3)
Source: eLife. 2019 Jan 4;8:e38187. doi: 10.7554/eLife.38187 (PMC6320072; doi:10.7554/eLife.38187)

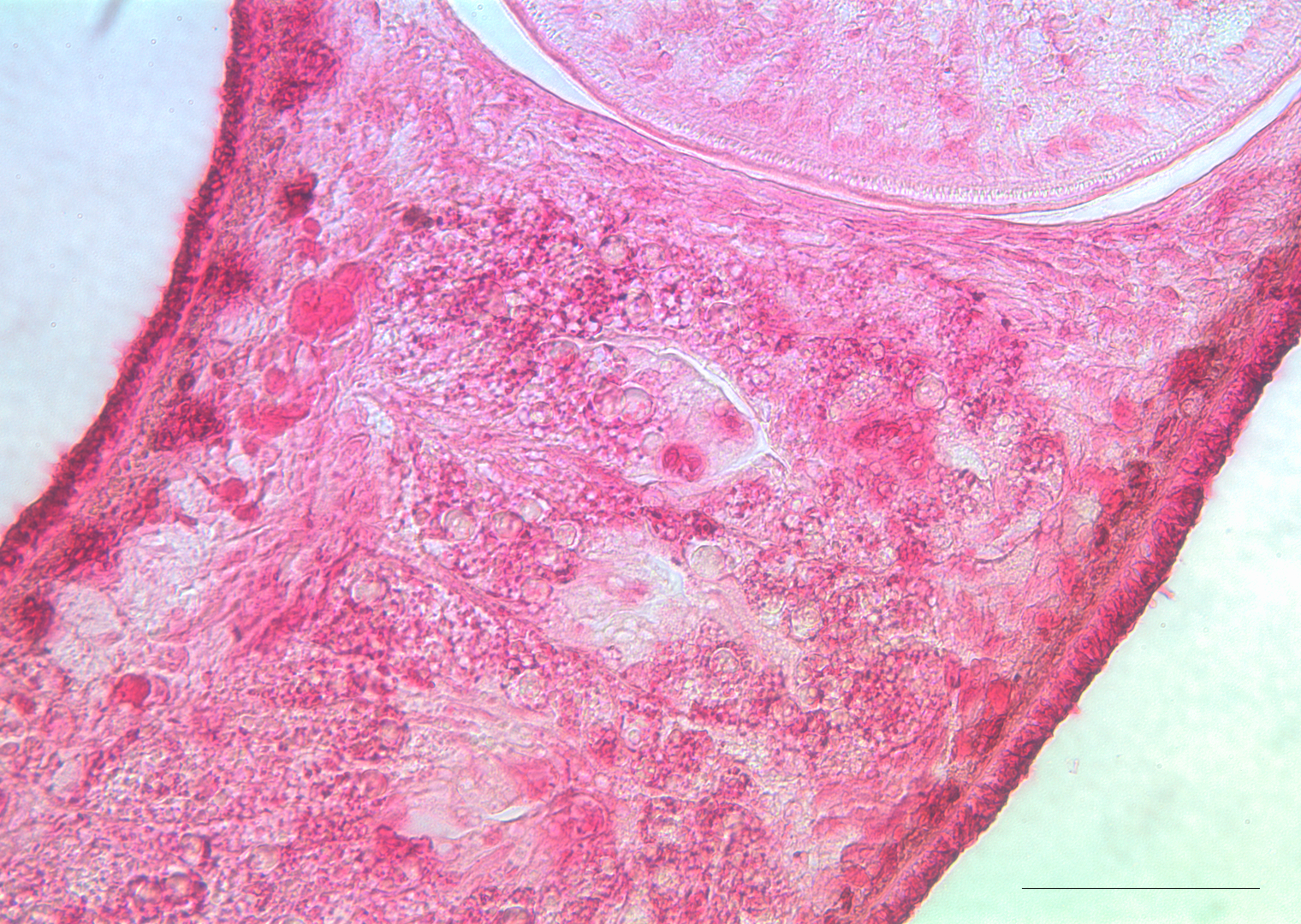

Supplement: Figure 4—source data 1. [file elife-38187-fig4-data1.zip › FIgure 4 - source data 1/Raw Images Glycogen/Large_noAG_10x.tif]

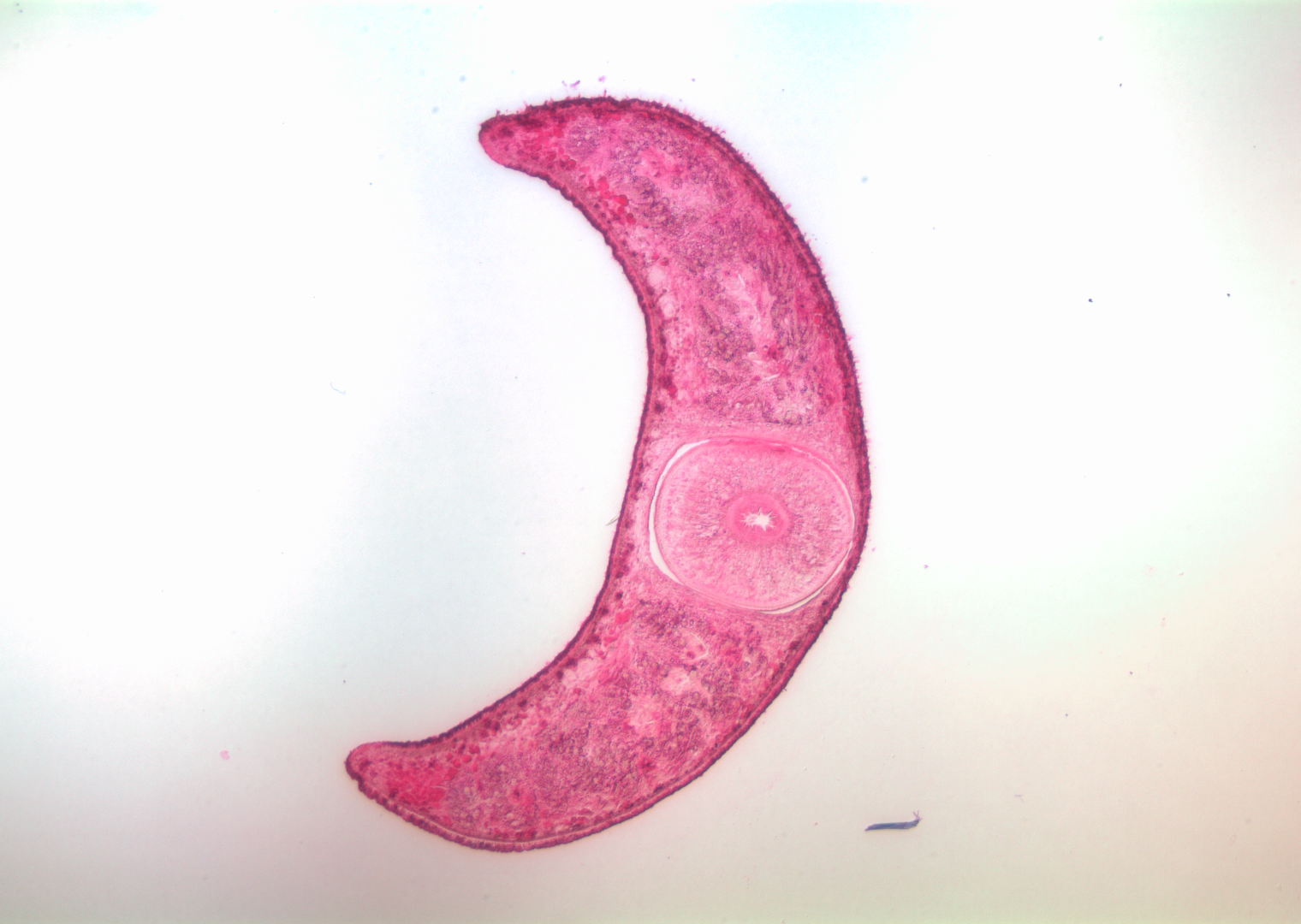

Supplement: Figure 4—source data 1. [file elife-38187-fig4-data1.zip › FIgure 4 - source data 1/Raw Images Glycogen/Large_noAG_5x.tif]

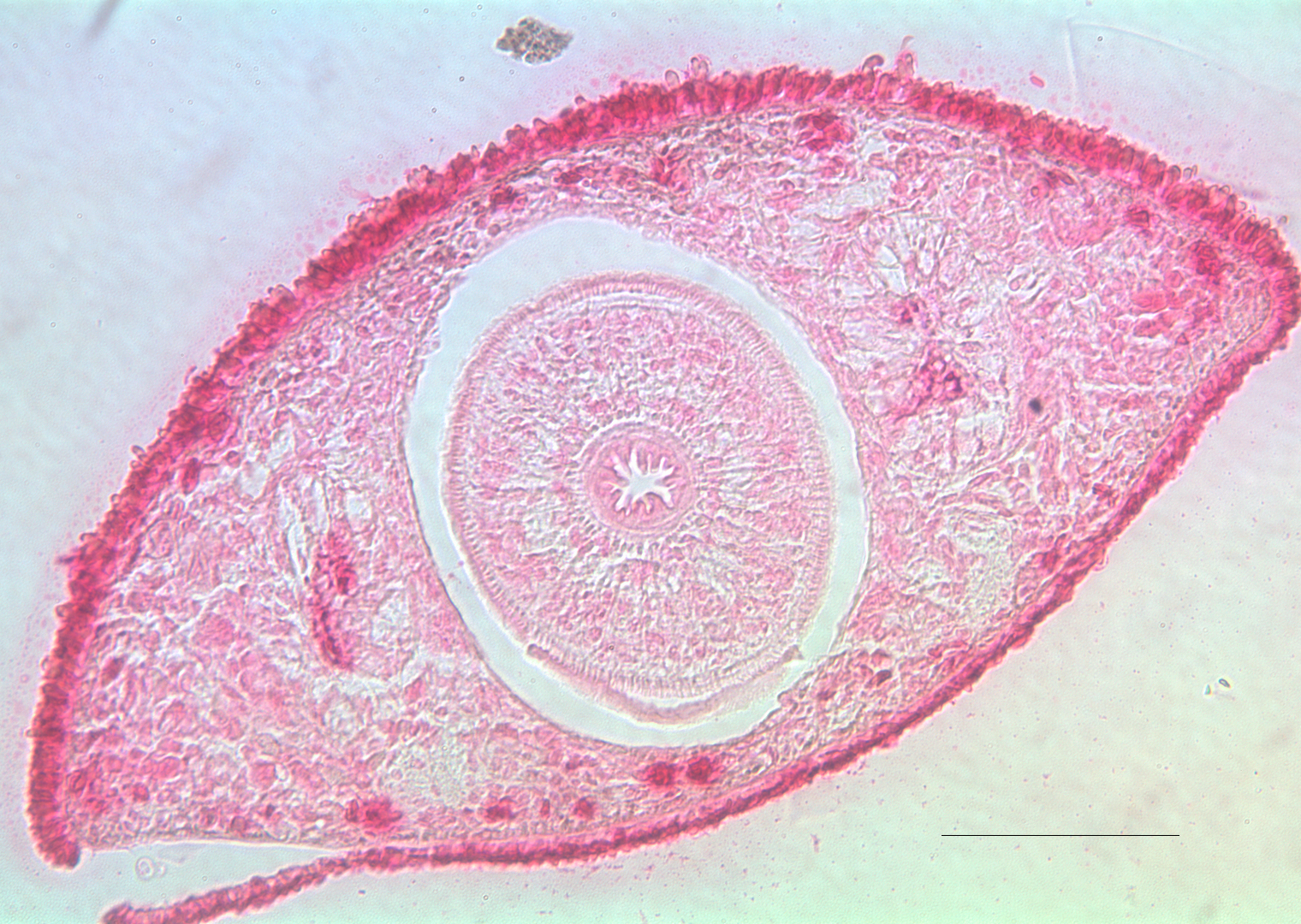

Supplement: Figure 4—source data 1. [file elife-38187-fig4-data1.zip › FIgure 4 - source data 1/Raw Images Glycogen/Small_AG_10x.tif]

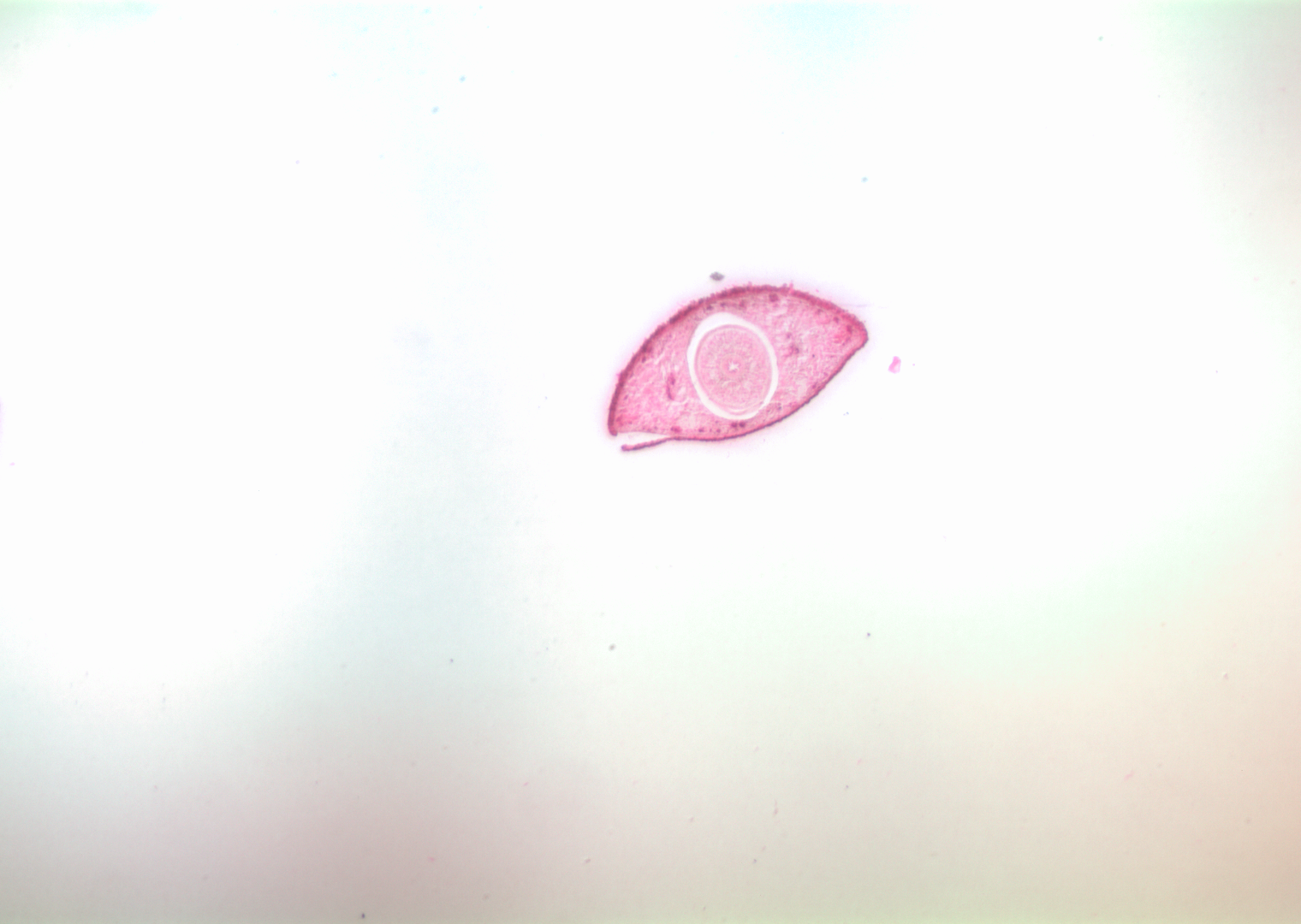

Supplement: Figure 4—source data 1. [file elife-38187-fig4-data1.zip › FIgure 4 - source data 1/Raw Images Glycogen/Small_AG_5x.tif]

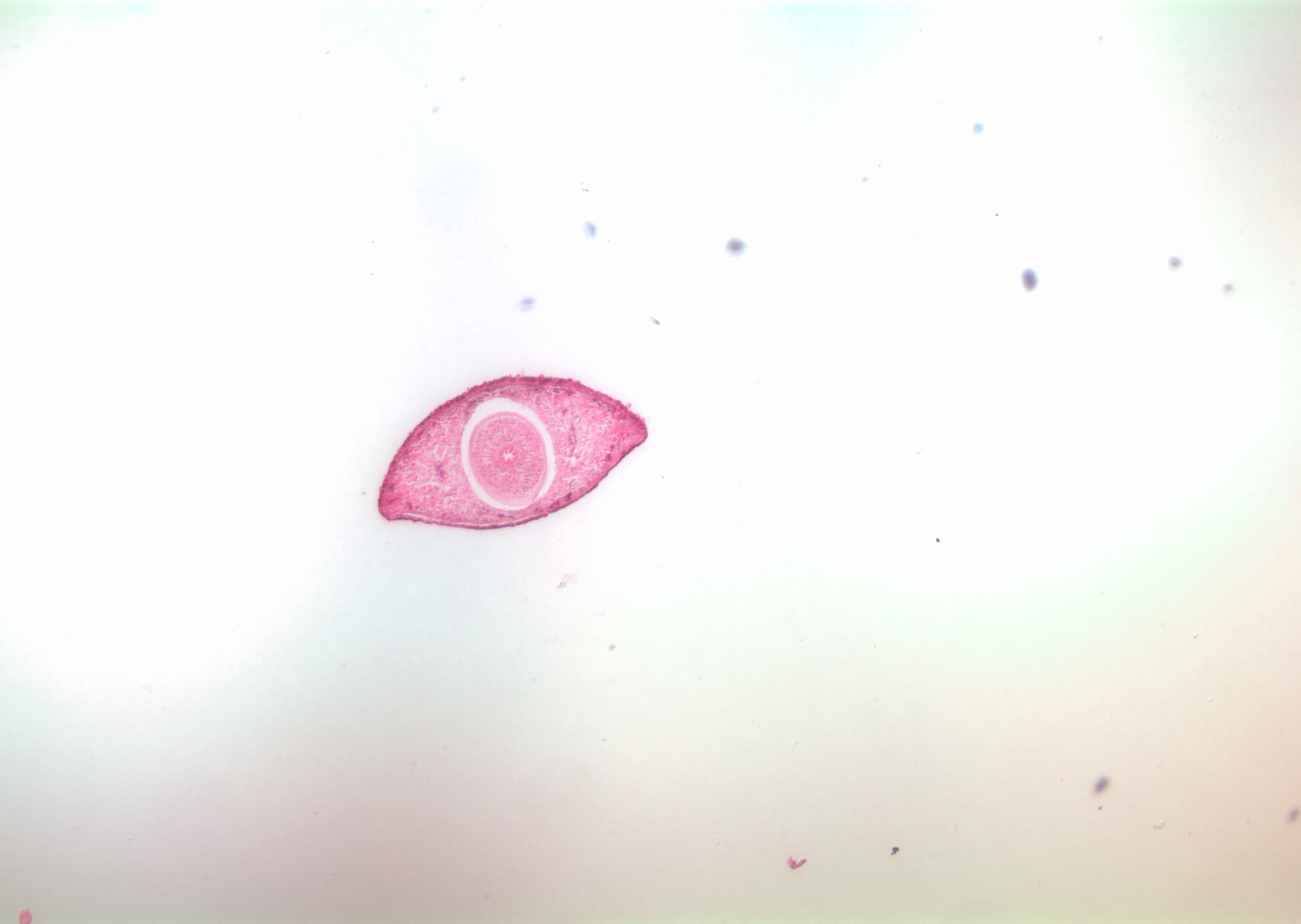

Supplement: Figure 4—source data 1. [file elife-38187-fig4-data1.zip › FIgure 4 - source data 1/Raw Images Glycogen/Small_noAG_5X.tif]

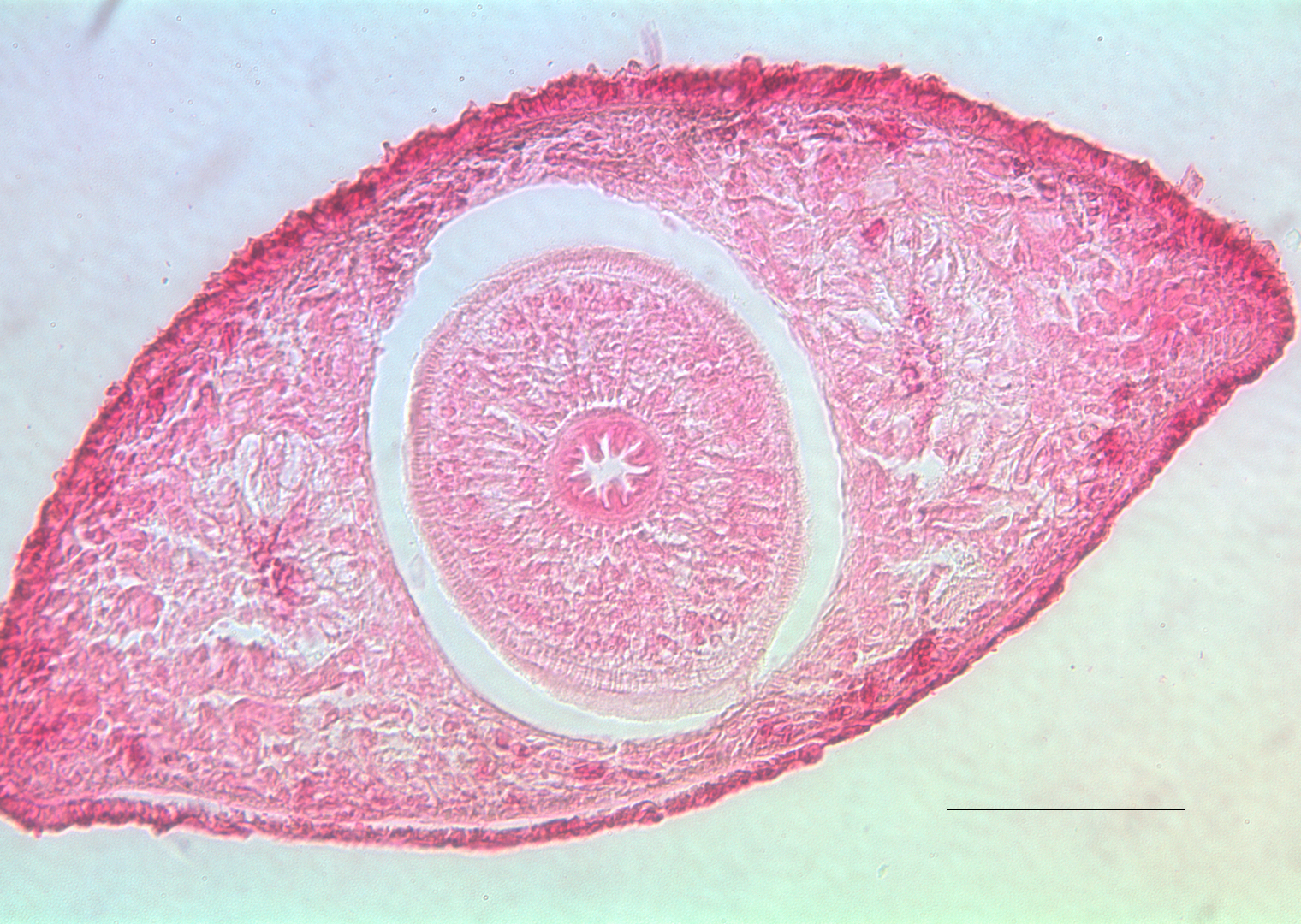

Supplement: Figure 4—source data 1. [file elife-38187-fig4-data1.zip › FIgure 4 - source data 1/Raw Images Glycogen/Small_noAS_10x.tif]

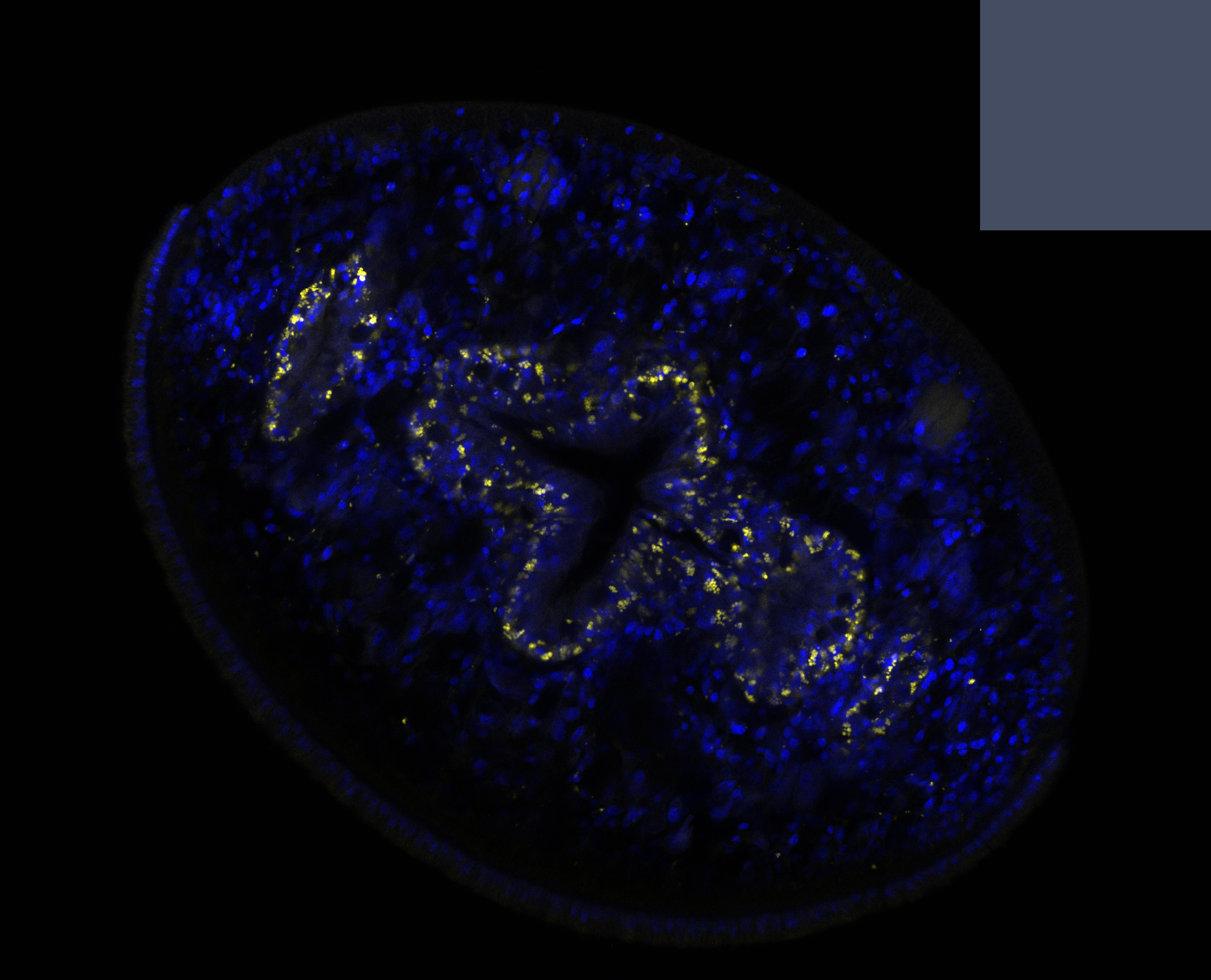

Supplement: Figure 4—source data 1. [file elife-38187-fig4-data1.zip › FIgure 4 - source data 1/Raw Images Lipid droplets/Small_stack.tif]
